# The phylogeny of pholcid spiders: a critical evaluation of relationships suggested by molecular data (Araneae, Pholcidae)

**DOI:** 10.3897/zookeys.789.22781

**Published:** 2018-10-10

**Authors:** Bernhard A. Huber, Jonas Eberle, Dimitar Dimitrov

**Affiliations:** 1 Alexander Koenig Research Museum of Zoology, Adenauerallee 160, 53113 Bonn, Germany Alexander Koenig Research Museum of Zoology Bonn Germany; 2 Center for Macroecology, Evolution and Climate, Natural History Museum of Denmark, University of Copenhagen, Copenhagen, Denmark University of Copenhagen Copenhagen Denmark; 3 Natural History Museum, University of Oslo, PO Box 1172 Blindern, NO-0318 Oslo, Norway University of Oslo Oslo Norway; 4 Current address: Department of Natural History, University Museum of Bergen, University of Bergen, PO Box 7800, NO-5020 Bergen, Norway University of Bergen Bergen Norway

**Keywords:** Biogeography, phylogeny, systematics, taxonomy

## Abstract

With almost 600 species, the latest molecular phylogeny of pholcid spiders (Eberle et al. 2018, BMC Evolutionary Biology) more than triples the largest previously available molecular phylogeny of the family. At the level of genera, the coverage is high (86%, i.e., 75 of the 87 named genera), and at the level of subfamilies it is complete. The present paper is an effort to critically evaluate the implications of this phylogeny for pholcid systematics. The analyses largely support the division of Pholcidae into five subfamilies: Ninetinae, Arteminae, Modisiminae, Smeringopinae, and Pholcinae. Their compositions are largely unchanged except that *Chisosa* Huber, 2000 is moved from Ninetinae to Arteminae. The positions of *Artema* Walckenaer, 1837 and *Priscula* Simon, 1893 in this system remain dubious. Relationships among subfamilies remain weakly supported, except for the sister group relationship between Smeringopinae and Pholcinae. Several major clades within subfamilies are separated from each other along geographical boundaries; for example within Modisiminae a South American clade and a Central + North American + Caribbean clade, and within Smeringopinae a Sub-Saharan clade and a clade ranging from the Mediterranean to Central Asia. Central + North American + Caribbean clades in both Ninetinae and Modisiminae may originate from South American ancestors.

Many taxonomic changes are suggested by the data, some of which are formally implemented herein. Two new genera result from the splitting of *Calapnita* Simon, 1892 and *Panjange* Deeleman-Reinhold & Deeleman, 1983, respectively: *Nipisa* Huber, **gen. n.**; and *Apokayana* Huber, **gen. n.** Nine new genera result from splitting of *Pholcus*: *Cantikus* Huber, **gen. n.**; *Kelabita* Huber, **gen. n.**; *Kintaqa* Huber, **gen. n.**; *Muruta* Huber, **gen. n.**; *Meraha* Huber, **gen. n.**; *Paiwana* Huber, **gen. n.**; *Pribumia* Huber, **gen. n.**; *Teranga* Huber, **gen. n.**; and *Tissahamia* Huber, **gen. n.** Two genera are newly synonymized: *Platnicknia* Özdikmen & Demir, 2009 is synonymized with *Modisimus* Simon, 1893; *Sihala* Huber, 2011 is synonymized with *Pholcus* Walckenaer, 1805. *Pholcusagadir* Huber, 2011 is moved to *Micropholcus* Deeleman-Reinhold & Prinsen, 1987, resulting in the new combination *Micropholcusagadir* (Huber, 2011).

## Introduction

Pholcidae is among the most species-rich spider families (World Spider Catalog 2018) and includes some of the spiders best known to the general public due to their occurrence in houses all over the world. Large amounts of morphological, taxonomic, behavioural, and biogeographic data on pholcids have been gathered and published over the last decades (http://www.pholcidae.de). Pholcidae is emerging as an ecologically highly diverse family that includes representatives with exceptional morphology and behaviour (e.g., asymmetric genitalia, ocular area modifications; highly regular webs; wrapping of prey with sticky silk; Huber and Nuñeza 2015, Huber et al. 2016c, Deeleman-Reinhold 1986a, Huber 2005b, Japyassú and Macagnan 2004), and that in some parts of the world is either extremely abundant (e.g., in East African forests; Sørensen et al. 2002) or has extreme levels of endemism (e.g., in Brazil’s Atlantic Forest; Huber and Rheims 2011, Huber 2015, 2016, 2018). However, convincing evolutionary interpretations are often impeded by insufficient phylogenetic resolution and by large gaps in the taxon sampling. The most recent molecular phylogeny of Pholcidae (Eberle et al. 2018) is undoubtedly a major step forward. Under the assumption that a good sample of taxa is possibly more important than an increase of characters/genes (cf. Graybeal 1998, Heath et al. 2008) we more than tripled the number of species as compared to the previous phylogeny of Dimitrov et al. (2013); many genera and major species groups were included for the first time. As far as the percentage of named genera included is concerned (86%), this is probably the most comprehensive molecular phylogeny of any major spider family so far. Despite this substantial increase in taxon sampling which has greatly improved our understanding of pholcid relationships, our tree remains a mosaic of ‘good’ and ‘bad’ parts: some nodes receive high support, others receive low or essentially no support. Revealingly, some support values changed dramatically among preliminary analyses of the present data. For example, unexpected clades with maximum support but contradicting any other evidence (e.g., morphology) suggested the existence of paralog sequences. In other cases, doubts persist but we were not able to identify problems with the molecular data. The idea of the present paper is thus to complement the primary phylogenetic data in Eberle et al. (2018) with a detailed account of arachnological implications and to look not only at but also beyond support values; we compare the molecular phylogeny with phylogenies derived from cladistic analysis of morphological characters and other information, and distinguish between clades that we consider a solid basis for further work and clades that we consider in need of further phylogenetic research.

## Material and methods

The trees presented here are derived from mitochondrial and nuclear gene sequences (12S, 16S, 18S, 28S, CO1, H3) gathered from 597 species of Pholcidae plus 32 outgroup species representing nine entelegyne and ten non-entelegyne families. For detailed specimen data, primers, lab protocols, alignment and tree inference algorithms, see Eberle et al. (2018). The present evaluation is based on four trees resulting from maximum likelihood analyses of data sets with varying degrees of missing data and unstable taxa, using two algorithms (RAxML, Stamatakis 2014; IQ-TREE, Nguyen et al. 2015). For the complete set of taxa, RAxML found the tree with the highest likelihood while the tree inferred with IQ-TREE was in better concordance with the known morphological evidence as suggested by cladistic analyses of morphological data and by qualitative character assessment (detailed in the respective sections below). Further trees were inferred with RAxML based on a reduced data set without rogue taxa (RogueNaRok, Wilkinson 1996, Sanderson and Shaffer 2002, Aberer et al. 2013) and on a “4+ genes” data set, including only those taxa for which four or more of the six target genes were available.

We calculated three types of branch support values for all trees: standard bootstrapping (SBS), rapid bootstrapping (RBS; Stamatakis et al. 2008), and Shimodaira–Hasegawa-like approximate likelihood ratio test (SH-like aLRT; Guindon et al. 2010). Terminal taxa are composed of a consistent string of five variables: (1) unique specimen code; (2) genus name, either scientific name, unique code, or “Gen. n.” for putatively new genera; (3) species name, either scientific name or unique code; (4) code for vial containing the specimen; (5) x's and o's, to respectively clarify the presence or absence of loci in the following order: 12S, 16S, 18S, 28S, CO1, H3.

We chose the tree from the IQ-TREE analysis for illustration and annotation because it appears more congruent with morphology. For the sake of clarity, only the RBS support is shown here; it may reflect true support most accurately (Anisimova et al. 2011). The same tree with all support values but without additional annotations is available as Supplementary file, together with the trees derived from RAxML from the complete and the two reduced, i.e., RogueNaRok, and “4+ genes” data sets.

**Figure 1.**
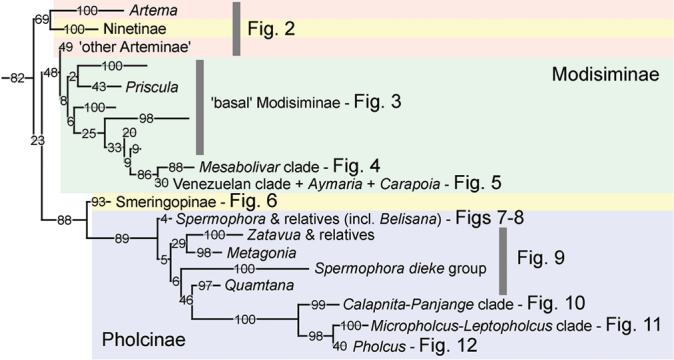
Backbone of the pholcid tree shown in Figs 2–12, derived from IQ-TREE analysis of the complete dataset.

To avoid overloading the text with numbers, we generally refer to the RBS support as follows: “low” (<70), “modest” (70–79), “reasonable” (80–94), “high” (95–99), or “full” (100) support. Even though the resolution of pholcid phylogeny has improved dramatically since 2011, the formal classification (Huber 2011b) into five subfamilies is not changed (Figure 1). Between the taxonomic levels of subfamilies and species we prefer to use informal names rather than tribes, subtribes, etc. Such unranked and formally unnamed taxa are less likely to burden future work as long as several major groups are still weakly supported and likely to change in composition or to be entirely rejected. The word “clade” is used like monophylum; thus, a clade can consist of subclades and those subclades are clades that again can consist of subclades. In general, colours in the phylogenies have no meaning beyond supporting the visual recognition of clades. The only exception is with *Belisana* Thorell, 1898, where litter and leaf-dwelling representatives are marked with different colours. Genus and species counts include the formal taxonomic changes herein. All measurements are in mm.

## Systematic accounts

### Subfamily Ninetinae

Taxon classificationAnimaliaAraneaePholcidae

Simon, 1890

Figure 2

Ninetinae Simon, 1890: 95. Type genus Ninetis Simon, 1890, by monotypy. Huber 2011b: 212.

#### Remarks.

Ninetinae are small to tiny ground-dwelling spiders that are largely restricted to arid environments (Huber and Brescovit 2003; BA Huber, unpublished data). With only 31 described extant species, the subfamily is by far the smallest of the five currently recognized subfamilies in Pholcidae. Ninetinae seem to be diverse in the New World (ten named genera + about four unnamed genera; BA Huber, unpublished data) where they represent the most southern (Argentina) and most northern (Canada) autochthonous pholcid records worldwide. Only two genera (*Ninetis* Simon, 1890 and one unnamed; BA Huber, unpublished data) are known from the Old World.

Their short legs make them superficially strikingly different from ‘typical’ long-legged pholcids. This distinctness was recognized as early as 1893, when Eugène Simon classified the only ninetine species available to him in a separate subfamily “Ninetidinae”, as opposed to all other pholcids classified in Pholcinae (Simon 1893). Subsequent morphological and molecular phylogenies have partly supported this view (Huber 2000, Dimitrov et al. 2013) but never convincingly with strong support.

**Figure 2.**
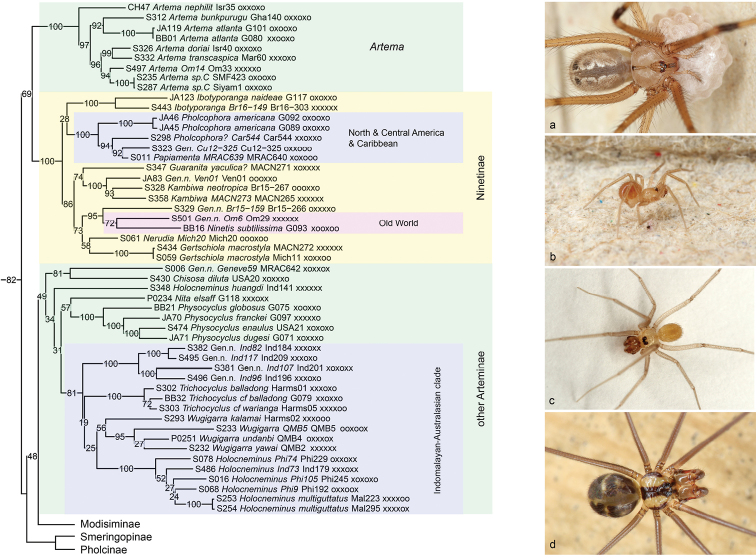
Ninetinae and Arteminae**a***Artema* sp. n. “Om14” (Oman) **b** Gen. n. (Ninetinae) sp. n. “Om6” (Oman) **c***Chisosadiluta* (USA) **d** Gen. n. (Arteminae) sp. n. “Ind82” (Sulawesi).

Our present analyses include 15 species representing eight of the eleven described genera, originating from both the New World and the Old World (Figure 2). A sister-group relationship between Ninetinae and all other pholcids is not supported by our analyses. Instead, all four analyses put Ninetinae as sister to *Artema* Walckenaer, 1837, and this clade is in turn sister to all other pholcids. For reasons discussed below (under Arteminae), we consider this relationship between *Artema* and Ninetinae dubious. The conclusion here is that Ninetinae are ‘basal’, either with *Artema* or without, but in any case the external relationships of Ninetinae remain unsatisfactorily resolved and need further study.

The monophyly of the subfamily receives high to full support in all analyses but the composition is slightly different from previous concepts: the North American *Chisosa* Huber, 2000, originally thought to be a representative of Ninetinae (Huber 2000), is moved to Arteminae. This move is also supported by male genitalic characters (massive palpal femur; procursus with dorsal apophysis and ventral pocket) and by somatic characters (exposed tarsal organ; reduction of epiandrous spigots; Huber 2000). Another genus that was previously (Huber and El Hennawy 2007, Huber 2011b) thought to be a member of Ninetinae is *Nita* Huber & El Hennawy, 2007. As already suggested in a previous analysis (Dimitrov et al. 2013), *Nita* is not a member of Ninetinae but of Arteminae.

The internal relationships of Ninetinae suggested by the molecular data are difficult to evaluate: they are mostly neither supported nor contradicted by morphological data. Two details are remarkable because they suggest that South America may not only be the most diverse region as far as Ninetinae are concerned but also the ancestral region of the subfamily. First, the analyses fully support a monophyletic North and Central American/Caribbean clade (*Pholcophora* Banks, 1896; *Papiamenta* Huber, 2000; and unidentified taxa from Cuba and Puerto Rico; “clade 2e” in Huber 2011b) that is either nested among South American ancestors or is sister to the South American *Ibotyporanga* Mello-Leitão, 1944 (with reasonable support in the 4+ genes tree only). Based on its geographic distribution, we predict that the Mexican *Tolteca* is also a member of this clade. Second, the two Old World genera (*Ninetis* and an undescribed genus from Oman) are also sister taxa (with low to modest support) and in all analyses (except for the 4+ genes analysis where *Ninetis* is missing) nested among South American taxa.

### Subfamily Arteminae

Taxon classificationAnimaliaAraneaePholcidae

Simon, 1893

Figure 2

Artemeae Simon, 1893: 463. Type genus Artema Walckenaer, 1837, by monotypy.
Arteminae Simon; Huber 2011b: 212.

#### Remarks.

All our analyses exclude the name-giving genus *Artema* from the clade containing all other Arteminae and invariably place *Artema* as sister to Ninetinae (Figure 2), formally precluding the use of the name Arteminae for this clade. We do not propose a new subfamily name for this clade but treat it as ‘other Arteminae’ because we consider the position of *Artema* dubious. *Artema* shares with ‘other Arteminae’ a unique pair of structures on the procursus: a ventral pocket and a dorsal apophysis. These structures are associated with asymmetric palp insertion in both species studied with respect to this detail [*Physocyclusglobosus* (Taczanowski, 1874), *Artemanephilit* Aharon et al., 2017: Huber and Eberhard 1997, Aharon et al. 2017]. The structures are present in all Arteminae, even in taxa that were previously thought to be representatives of other subfamilies, such as *Chisosa* and *Nita* (previously in Ninetinae; see above), and *Wugigarra* Huber, 2001 (previously in Modisiminae; see below) (Huber 2000, 2001, Huber and El Hennawy 2007). By contrast, these structures are apparently absent in all other Pholcidae. Curiously and unexplainable to us, previous molecular analyses have supported a position of *Artema* among ‘other Arteminae’ (Astrin et al. 2007: fig. 1, Dimitrov et al. 2013).

Some of the 99 currently known species of Arteminae are relatively large spiders with long, strong legs and high globose abdomens. The genus *Artema*, in particular, includes probably the largest pholcids in terms of body mass (Aharon et al. 2017). However, tiny species that were previously assigned to Ninetinae partly because of their size (*Chisosa*, *Nita*) are now included in Arteminae, and their ‘basal’ position in the cladogram suggests that ancestral Arteminae may in fact have been tiny. Just like Ninetinae, Arteminae often occur in rather dry regions, sometimes even in deserts like the Australian *Trichocyclus* Simon, 1908. They have a wide distribution, but are apparently absent from Sub-Saharan Africa and from South America (except for “Geneve59”, a tiny undescribed species representing a new undescribed genus on Curaçao and Aruba).

The monophyly of ‘other Arteminae’ is supported in all our analysis, even though with low support (possibly because of the dubious position of *Artema*, see above). Similar to our previous analysis (i.e. except for the position of *Artema*; Dimitrov et al. 2013), ‘other Arteminae’ is sister to Modisiminae, with variable support (reasonable support only in the RogueNaRok tree; in other trees, bootstrap support is low but SH values range from 82 to 99). This sister group relationship is weakly supported by morphology: ‘other Arteminae’ and Modisiminae lack epiandrous spigots. However, epiandrous spigots have been lost several times convergently in Pholcidae (Huber 2000, BA Huber, unpubl. data).

Internal relationships in ‘other Arteminae’ are partly resolved with reasonable support. The data suggest a large Indomalayan-Australasian clade, including the genera *Trichocyclus* and *Wugigarra* (Australia), *Holocneminus* Berland, 1942 (SE Asia and Pacific; excluding the misplaced and highly isolated *H.huangdi* Tong & Li, 2009), and a new undescribed genus (without any described species; ranging from Eastern Indonesia to the Pacific). Sister to this clade is either the New World genus *Physocyclus* Simon, 1893 alone or *Physocyclus* together with the Middle-Eastern monotypic *Nita*. However, support values for any of these options are low and morphological data do not favour (nor contradict) any of them. Finally, the ‘basal’ branches, i.e., those leading to the taxa outside the Indomalayan-Australasian clade and *Physocyclus* (and *Nita* in the case of the IQ-TREE analysis) lead to a group of North American and Caribbean taxa (the North American genus *Chisosa* being sister to a tiny undescribed species representing a new undescribed genus on Curaçao and Aruba: “Geneve59”), and to the SE-Asian *Holocneminushuangdi*, an isolated species that appears misplaced also by morphological criteria (A Valdez-Mondragón, pers. comm., Nov. 2015).

### Subfamily Modisiminae

Taxon classificationAnimaliaAraneaePholcidae

Simon, 1893

Figs 3 , 4 , 5

Modisimeae Simon, 1893: 484. Type genus Modisimus Simon, 1893, by subsequent designation (Huber 2011b).
Modisiminae Simon; Huber 2011b: 216.

#### Remarks.

Modisiminae are the typical pholcids of the humid Neotropics, where they occupy a wide variety of microhabitats from leaf litter to high among the vegetation. This ecological variability is paralleled by a wide range of body forms, from tiny ground-dwelling forms (e.g., Gertsch 1982, Huber and Rheims 2011) to some of the largest pholcids with leg spans of over 15 cm (e.g., Huber and Astrin 2009, Huber 2015, 2018). With currently 480 species in 24 genera, Modisiminae is one of the two large subfamilies of Pholcidae, with several species-rich genera (e.g., *Anopsicus* Chamberlin & Ivie, 1938; *Psilochorus* Simon, 1893; *Modisimus* Simon, 1893; *Mesabolivar* González-Sponga, 1998; *Carapoia* González-Sponga, 1998) and many undescribed species.

All previous analyses have supported this group (Huber 2000, 2001, Bruvo Mađarić et al. 2005, Dimitrov et al. 2013), even though with minor differences in composition. The equivalent ‘New World clade’ in Huber (2001) still included the Australian *Wugigarra*, a genus that has since been moved to Arteminae (Dimitrov et al. 2013). As a result, Modisiminae is now considered to be restricted to the New World.

**Figure 3.**
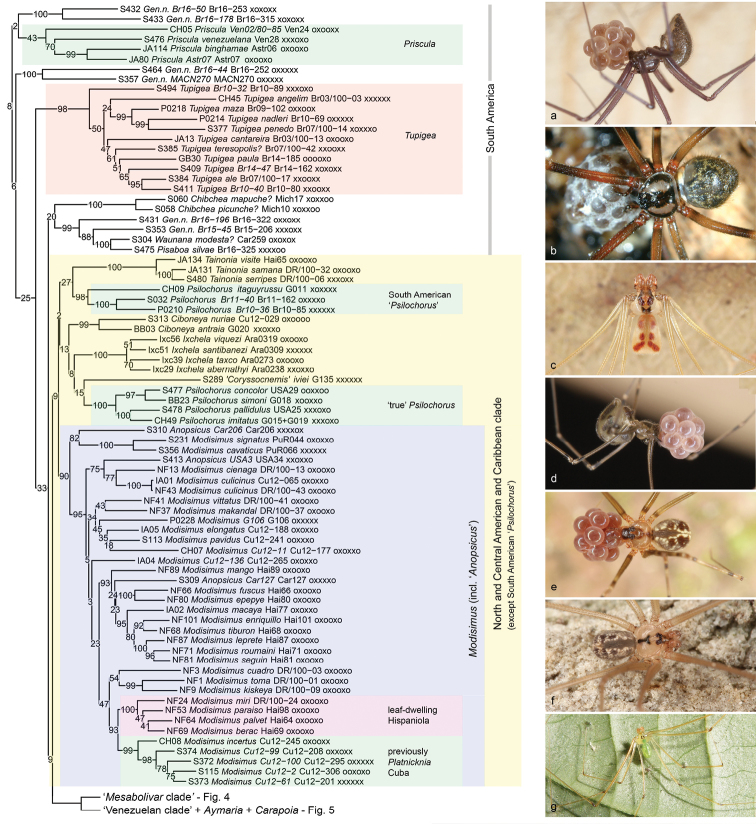
‘Basal’ Modisiminae **a** Gen. n., sp. n. “Br16-50” (Brazil) **b***Prisculaandinensis*? (Venezuela) **c** Gen. n., sp. n. “Br16-196” (Brazil) **d***Tupigea* sp. n. “Br14-47” (Brazil) **e***Pisaboasilvae* (Brazil) **f***Psilochorusimitatus* (USA) **g***Modisimusincertus* (Cuba).

Our analyses all recover Modisiminae, but with very low support values. This is possibly due to the mysterious Andean genus *Priscula* Simon, 1893 (Figure 3) that is either included in Modisiminae (IQ-TREE) or not (RAxML). The position of *Priscula* has always been considered problematic. Simon (1893) created a separate taxon “Prisculeae” for this genus; Brignoli (1981) synonymized it with *Physocyclus*; the first morphological cladistic analysis (Huber 2000) supported the position of *Priscula* near *Physocyclus* but this result was explicitly doubted (Huber 2000: 129). In the molecular analysis of Dimitrov et al. (2013)*Priscula* was excluded because the positions of the included species varied dramatically among different types of analyses. Morphologically, *Priscula* differs from (other) Modisiminae by the presence of ALS piriform gland spigots and by the absence of a retrolateral apophysis on the male palpal coxa (Huber 2000), i.e., it has retained plesiomorphic characters. A sister-group relationship between *Priscula* and other Modisiminae appears thus plausible from a morphological point of view.

Despite the low support values, we thus consider Modisiminae (including *Priscula* or not) a likely monophyletic group. Several morphological characters support Modisiminae (incl. *Priscula*): an exposed tarsal organ; the reduction of epiandrous spigots (shared with ‘other Arteminae’; see above); and a large distance between ALE and PME (Huber 2000). As indicated above (section Arteminae) our data weakly support a sister-group relationship between ‘other Arteminae’ and Modisiminae.

Within Modisiminae, many support values are extremely low, and the suggested relationships are thus unreliable (Figure 3). In addition, taxon sampling is very uneven, with some genera well represented (e.g., *Carapoia*, *Mesabolivar*, *Modisimus*), and others poorly represented or entirely missing (see below). However, several results are consistent among analyses and noteworthy for various reasons: they suggest groups that appear feasible in terms of biogeography; they suggest interesting evolutionary scenarios; and they suggest formal taxonomic changes, some of which have been suggested before based on morphology.

Apart from *Priscula*, the ‘basal’ branches within Modisiminae lead to small South American unnamed taxa (Figure 3). In particular, the two species “Br16-44” and “MACN270” are both tiny, with body lengths of 0.9 and 1.3 mm, respectively. Other ‘basal’ branches lead to an unnamed Amazonian genus (“Br16-178” and “Br16-50”; body lengths: 1.5–1.8 mm) and the Atlantic Forest genus *Tupigea* Huber, 2000 (body lengths: 1.3–1.9 mm; Huber 2000, Huber and Rheims 2011). This suggests a similar evolutionary scenario as proposed for ‘other Arteminae’ above, i.e., that ancestral Modisiminae may have been small ground-dwelling species. *Priscula* is once again the disturbing factor in this scenario: all known representatives of *Priscula* are medium-size to large spiders (Huber 2000), possibly surpassed (as far as body mass is concerned) by *Artema* only. In both Arteminae and Modisiminae, the emerging picture is one of medium-sized forms missing or disappearing early, large forms experiencing little subsequent changes in body shape and poor subsequent speciation (*Artema*: currently eight species; *Priscula*: currently 17 species), and small forms diversifying dramatically in size, shape, and numbers (‘other Arteminae’: currently 91 species; Modisiminae without *Priscula*: currently 463 species).

The next branch (Figure 3; *Chibchea* Huber, 2000 to *Pisaboa* Huber, 2000) includes several South American genera, some of them diverse but poorly represented in our analyses (e.g., *Chibchea*). The close relationship between *Pisaboa* and *Waunana* Huber, 2000 was already suggested in the original descriptions of these genera (Huber 2000), even though based on highly homoplastic characters (vertical hairs on male leg tibiae in high density; shape of apophysis on male palpal femur). A close relationship of these two genera with *Chibchea* either receives very low support (IQ-TREE, RAxML) or is not recovered (RogueNaRok); it is neither supported nor contradicted by morphology. Clearly, this clade needs a much denser sampling and the addition of missing taxa that are possibly related (e.g., *Pomboa*).

The next clade (Figure 3) includes all North and Central American and Caribbean taxa, suggesting that the ancestor of this clade arrived in the region from South America. This scenario was explicitly rejected by Dimitrov et al. (2013) based on the supposed age of the group (~120–170 Ma). However, our upcoming analysis has not been able to confirm this age (Eberle et al. 2018; we were not able to calculate convincing absolute ages from the data). The clade is recovered in most analyses (it is paraphyletic in the 4+ genes tree) but always with low support (only SH values are reasonable to high). The only geographic outlier in this clade is South American ‘*Psilochorus*’. North American (‘true’) *Psilochorus* and South American ‘*Psilochorus*’ each receive high to full support but are never resolved as sister taxa. Whether South American ‘*Psilochorus*’ are ancestral within this large clade or represent a case of back-colonization is currently impossible to say; the internal nodes in this clade have partly too low support to favour a particular scenario. The inclusion of the Central American *Ixchela* Huber, 2000 in this clade fits the geographic pattern and contradicts a previous speculation (in Huber 2000) that *Ixchela* might be close to the South American genus *Aymaria* Huber, 2000. In much the same way, the only Central American representative of *Coryssocnemis* Simon, 1893 included in our analyses is placed in this group, far away from ‘true’ South American *Coryssocnemis* (the polyphyly of *Coryssocnemis* has long been suspected: Gertsch 1971, Brignoli 1981, Huber 1998, 2000). The Cuban endemic genus *Platnicknia* Özdikmen & Demir, 2009 is deeply nested within the large genus *Modisimus*. It is resolved as sister to a distinctive group of Hispaniolan leaf-dwelling representatives of *Modisimus* (the “leaf-dwelling species group” in Huber et al. 2010) and synonymized below. Finally, the large genus *Anopsicus* (63 described species) is poorly represented in our analyses. The three species included are all undescribed, do not group together, and are nested among *Modisimus*. Since neither the type species of *Anopsicus* is included nor is a potential close relative (or at least another species from Yucatán), the monophyly and position of *Anopsicus* both remain dubious.

**Figure 4.**
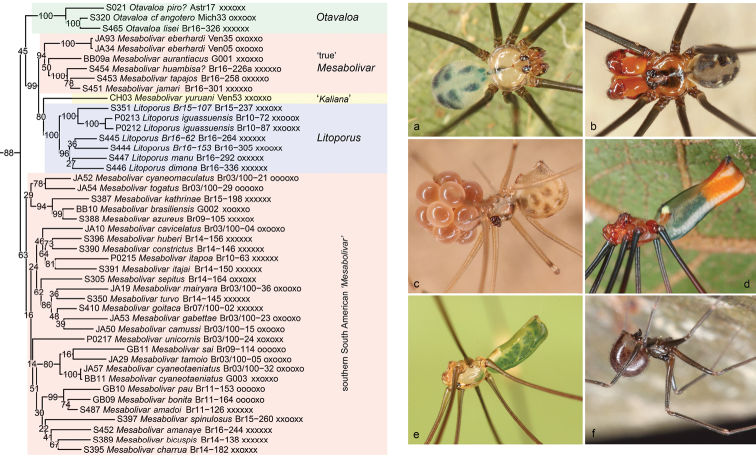
*Mesabolivar* clade **a***Otavaloalisei* (Brazil) **b***Mesabolivarmaraba* (Brazil) **c***Litoporus* sp. n. “Br16-153” (Brazil) **d***Mesabolivarcyaneotaeniatus* (Brazil) **e***Mesabolivarkathrinae* (Brazil) **f***Mesabolivarsaci* (Brazil).

**Figure 5.**
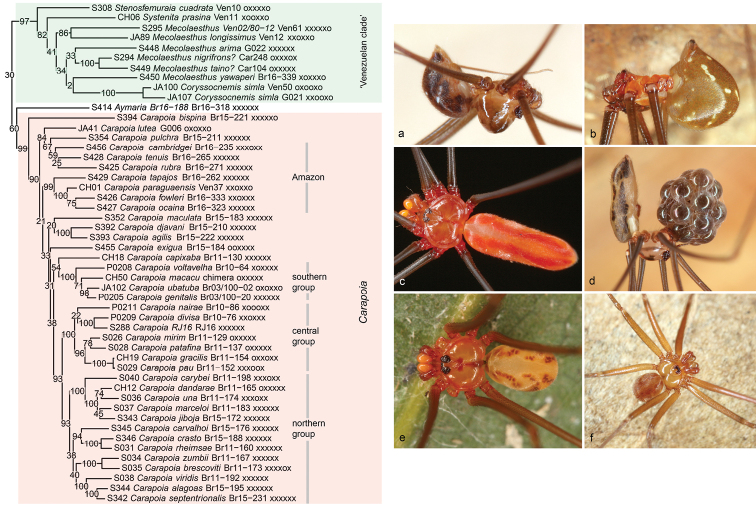
Venezuelan clade + *Aymaria* + *Carapoia***a***Mecolaesthusyawaperi* (Brazil) **b***Aymaria* sp. n. “Br16-188” (Brazil) **c***Carapoiarubra* (Brazil) **d***Carapoiakaxinawa* (Brazil) **e***Carapoiapulchra* (Brazil) **f***Carapoiaagilis* (Brazil).

Sister to the previous North and Central American and Caribbean clade is another large, entirely South American clade (Figure 3, bottom). The sister-group relationship is very poorly supported, but the monophyly of the South American clade has modest (4+ genes) to reasonable (RogueNaRok) support. It is divided into three subclades with reasonable to full support plus the genus *Aymaria* that is represented by a single species and whose position within this clade is not convincingly resolved. The first subclade included is here informally called the ‘*Mesabolivar* clade’ (Figure 4); the second subclade is largely Venezuelan and thus called ‘Venezuelan clade’ (Figure 5); the third subclade is the genus *Carapoia* (Figure 5).

Within the ‘*Mesabolivar* clade’ (Figure 4), our analyses suggest two specific relationships that are likely to have drastic taxonomic consequences. First, *Litoporus* Simon, 1893 is nested among ‘true’ northern South American *Mesabolivar*. This has been suggested before (Dimitrov et al. 2013), but that previous analysis included a single species of *Litoporus* whose generic identity was uncertain (Huber et al. 2013). The present analyses include several unambiguous (Amazonian) representatives of *Litoporus*. Our data support the monophyly of *Litoporus* (full support) but also its position within *Mesabolivar* (reasonable to high support). Second, *Mesabolivar* is composed of two sub-clades: ‘true’ northern South American *Mesabolivar*, and southern South American (largely Atlantic Forest) ‘*Mesabolivar*’. The southern sub-clade includes the monotypic genus *Teuia* Huber, 2000 (synonymized with *Mesabolivar* in Huber 2018; the type species of *Teuia* is not included but a putatively closely related species: *M.sepitus*). Potential formal taxonomic changes are discussed in the Taxonomy section below. The close relationship between *Otavaloa* Huber, 2000 and *Mesabolivar* is neither supported nor contradicted by morphological data.

The ‘Venezuelan clade’ (Figure 5) receives high to full support and is composed of several genera that are either known from Venezuela only (*Systenita* Simon, 1893, *Stenosfemuraia* González-Sponga, 1998), from Venezuela and Trinidad and Tobago (*Coryssocnemis*), or from Venezuela plus neighboring countries (*Mecolaesthus* Simon, 1893). A close relationship among these genera had been suspected before based on morphology (Huber 2000), and molecular data have always supported this (Bruvo-Mađarić et al. 2005: 28S data and combined analysis; Dimitrov et al. 2013). Our data suggest that *Coryssocnemis* may be nested within *Mecolaesthus*, but our taxon sampling is weak, the topology is unstable (*Systenita* is either nested within *Mecolaesthus* or not), and several internal nodes in the clade have low support. Formally, *Coryssocnemis* still includes several obviously misplaced species: several Central American species (see above), and several Atlantic Forest (Brazilian) species whose identity is probably impossible to resolve (poor descriptions, lost types; see Huber 2000, 2018).

The third subclade in the South American clade is *Carapoia* (Figure 5). Unlike *Mesabolivar* it is monophyletic and apparently less problematic, but just as *Mesabolivar*, the genus has become very difficult to diagnose, mainly because of ‘untypical’ species added to the genus based in large part on the present molecular data (Huber 2018). Both for *Mesabolivar* and *Carapoia* our analyses suggest several species groups that are also supported by morphological data. For a detailed discussion of these groups, see Huber (2018).

### Subfamily Smeringopinae

Taxon classificationAnimaliaAraneaePholcidae

Simon, 1893

Figure 6

Smeringopodeae Simon, 1893: 474. Type genus Smeringopus Simon, 1890, by subsequent designation (Huber 2011b).
Smeringopinae Simon; Huber 2011b: 217.

#### Remarks.

Smeringopinae is a relatively homogeneous subfamily (with respect to body shapes, colour, webs, and microhabitats), and in this sense similar to Ninetinae and Arteminae but very unlike Modisiminae and Pholcinae. Most of the 125 known species of Smeringopinae are medium-size to large, have long legs, elongated to cylindrical abdomens, and all have eight eyes. Another similarity to Ninetinae and Arteminae is that Smeringopinae are often found in rather arid regions. The most obvious exception is the largely humid tropical genus *Smeringopina* Kraus, 1957, which is also the genus with the smallest and largest representatives in the subfamily (with body lengths ranging from 2.5–10 mm) and with the widest range of microhabitats used (leaf litter to large sheltered spaces) (Huber 2013). The original distribution of the subfamily is Africa, the Mediterranean, and the Middle East. Three species have attained much wider distributions, resulting from human-mediated dispersal (Huber 2011b).

As in previous molecular analyses (Bruvo-Mađarić et al. 2005, Astrin et al. 2007, Dimitrov et al. 2013), Smeringopinae is sister to Pholcinae (Figure 1) with reasonable to high support. This relationship is also supported by morphology: the two taxa share tarsus IV comb-hairs spread over the entire length of the tarsus (Huber and Fleckenstein 2008).

The monophyly of Smeringopinae receives reasonable to high support in all our analyses. Previous molecular analyses have partly supported Smeringopinae, but also suggested rather obscure relationships [e.g., the position of *Holocnemuspluchei* (Scopoli, 1763) among Ninetinae in Astrin et al. 2007]. *Holocnemuspluchei* was included in preliminary analyses of the present data but its position was drastically unstable, so we decided to exclude it from the final analyses. Smeringopinae monophyly is rather weakly supported by morphology, i.e., by the presence of a large thoracic pit on the carapace (rather than a narrow furrow or an evenly domed carapace; cf. Huber 2011b).

**Figure 6.**
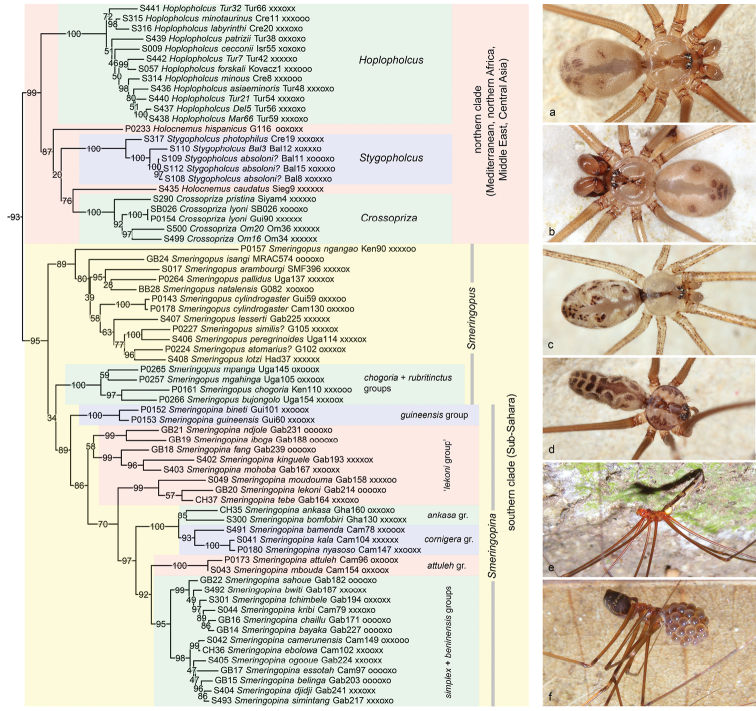
Smeringopinae **a***Hoplopholcus* sp. n. “Mar66” (Turkey) **b***Stygopholcusabsoloni*? (Bosnia and Herzegovina) **c***Crossopriza* sp. n. “Om11” (Oman) **d***Smeringopuspallidus* (Philippines) **e***Smeringopinapulchra* (Ghana) **f***Smeringopinaankasa* (Ghana).

Within Smeringopinae, our data strongly support a basal split between a northern clade (Mediterranean, northern Africa, Middle East, Central Asia) and a southern clade (Sub-Sahara) (Figure 6). This basal split was also recovered in a morphological cladistic analysis (Huber 2012). Within the northern clade, *Hoplopholcus* Kulczynski, 1908 is sister to all other genera and not close to *Stygopholcus* Kratochvil, 1932 as repeatedly claimed by Brignoli (1971, 1976, 1979) but contested by Senglet (1971, 2001). The genera *Hoplopholcus*, *Stygopholcus*, and *Crossopriza* Simon, 1893 all receive full support, but the small Mediterranean genus *Holocnemus* Simon, 1873 (only three described species) continues to be problematic even after the exclusion of *H.pluchei*. The two species of *Holocnemus* included in our analyses never group together, and no morphological synapomorphy is known to suggest their sister-group relationship (in fact, *Holocnemus* has never been revised).

The southern (Sub-Saharan) clade includes *Smeringopus* Simon, 1890 and *Smeringopina*, and is also supported by a unique number of epiandrous spigots (two) (Huber 2012). The paraphyly of *Smeringopus* has been suggested before (Dimitrov et al. 2013), and our larger data set supports this view, but with low support values. Two of the species groups of *Smeringopus* proposed in Huber (2012) appear closer to *Smeringopina* than to other *Smeringopus*: the *chogoria* group and the *rubrotinctus* group. Morphological data do not support this view but they neither strongly contradict it: the two species groups lack the distinctive arrangement of pores on the pore plates (in groups or ‘islands’) and the retrolateral furrow on the male palpal femur present in all other species of *Smeringopus* (Huber 2012). Remarkably, *Smeringopus* and *Smeringopina* are largely separated geographically, with *Smeringopus* being most diverse in southern and eastern Africa, and *Smeringopina* in western and central Africa (Huber 2012, 2013). The *chogoria* and *rubrotinctus* groups are geographically restricted to an area where Central Africa (the Guineo-Congolian center of endemism) meets East Africa (Huber 2012). Other than that, our sampling in *Smeringopus* is not dense enough to test the species groups proposed in Huber (2012). Remarkably, though, the isolated ‘basal’ position of *S.ngangao* Huber, 2012 is supported by the present analyses.

Our analyses include 30 of the 44 described species of *Smeringopina* (68%), and all species groups proposed in Huber (2013) except two monotypic ‘groups’ (*S.fon* Huber, 2013; *S.ngungu* Huber, 2013). Even though for some species only one gene (CO1) was sequenced, our analyses support several species groups and deeper relationships proposed previously (Huber 2013), based on cladistic analysis of morphological characters. Morphology placed the West African *guineensis* group as sister to all other *Smeringopina*; all our analyses support both the monophyly of the *guineensis* group and its sister-group relationship with all other congeners. The next two branches are composed of representatives of the *lekoni* group, which is thus here considered paraphyletic rather than monophyletic. The *ankasa* and *cornigera* groups are both supported, as is their sister group relationship to each other. The *attuleh* group is supported, but not as sister to the *ankasa* + *cornigera* groups but as sister to the following group. The last clade is composed of representatives of the *simplex* and *beninensis* groups, but the clear dichotomy in the molecular trees is not equivalent to these groups. Instead, the *simplex* group includes all ‘basal’ representatives originally assigned to the *beninensis* group; the *beninensis* group includes only those species that have a light transversal element ventrally on the abdomen (character 9 in Huber 2013, which is thus less homoplastic than previously thought).

### Subfamily Pholcinae

Taxon classificationAnimaliaAraneaePholcidae

C.L. Koch, 1850

**Figure 7.**
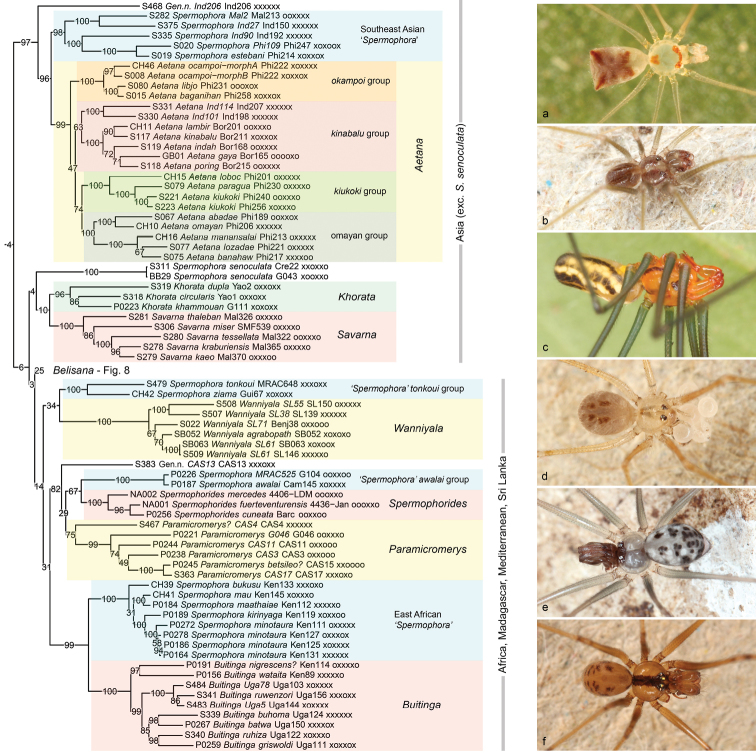
Pholcinae ‘group 1’ (*Spermophora* and relatives) **a** Gen. n., sp. n. “Ind206” (Halmahera); b‚ *Spermophora* sp. n. “Ind27” (Sumatra) **c***Aetanabaganihan* (Philippines) **d***Spermophorasenoculata* (Turkey) **e***Savarnatessellata* (Thailand) **f***Wanniyalaagrabopath* (Sri Lanka).

**Figure 8.**
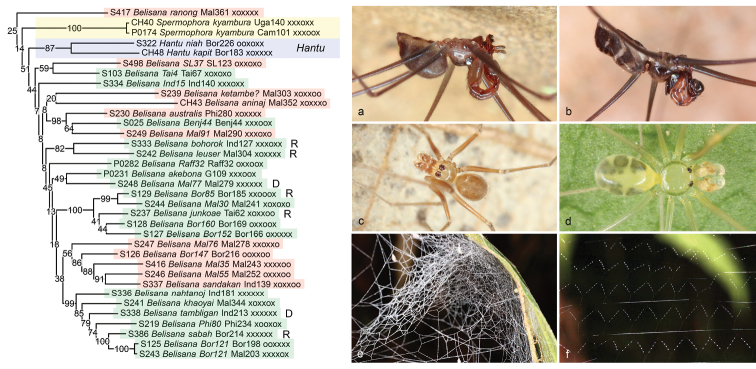
*Belisana* and *Hantu*. For *Belisana*, the background colours signify microhabitat: red = ground; green = leaf. D, domed web; R, highly regular ‘curtain’ web. Photos **a***Hantuniah* (Sarawak) **b***Hantukapit* (Sarawak) **c***Belisanasandakan* (Sumatra) **d***Belisanasabah* (Sabah) **e** domed web of *Belisana* sp. n. “Mal77” (Malaysia) **f** regular ‘curtain’ web of *Belisanabohorok* (Sarawak).

**Figure 9.**
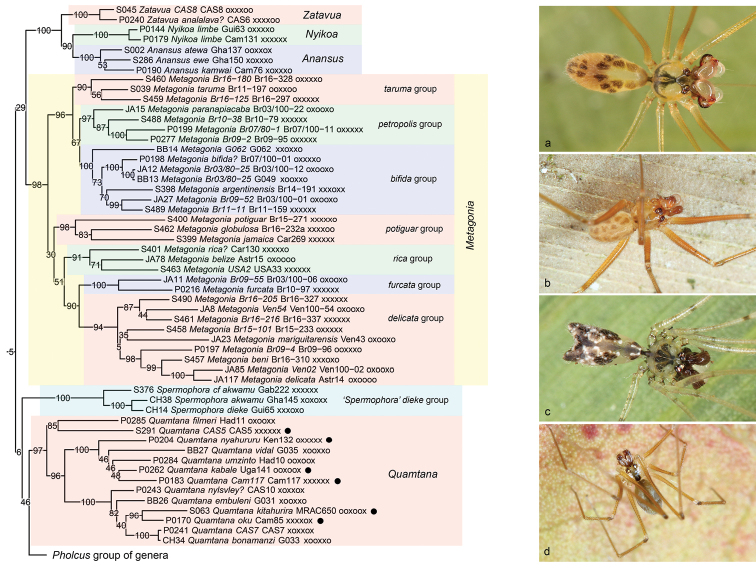
Pholcinae ‘group 2’ (*Zatavua* and relatives, *Metagonia*), and *Quamtana* (marked: non-South African species). Photos **a***Metagoniataruma* (Brazil) **b***Metagonia* sp. n. “Br07-1” (Brazil) **c***Metagoniabifida*? (Brazil) **d***Quamtana* sp. n. (cf. *mabusai*) (Germany).

**Figure 10.**
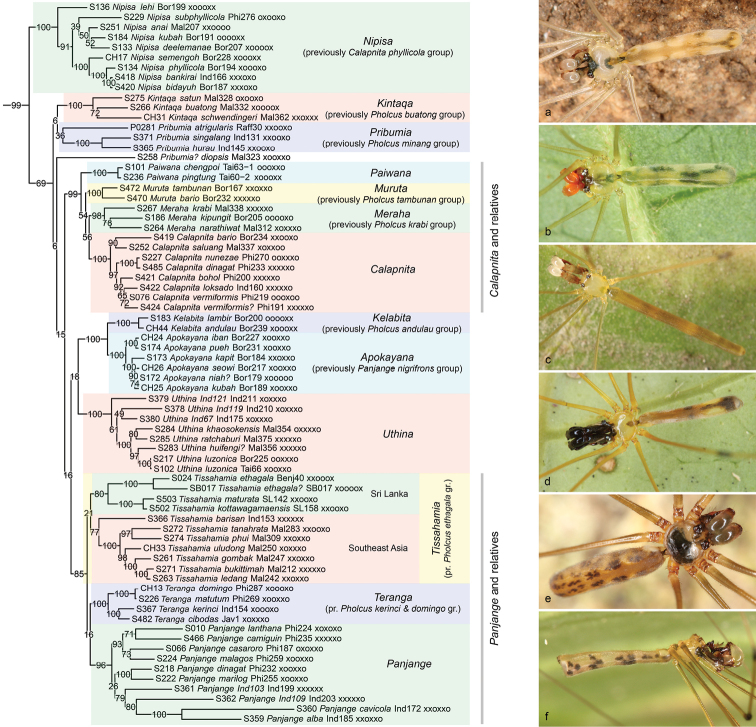
*Calapnita-Panjange* clade **a***Kintaqasatun* (Malaysia) **b***Merahanarathiwat* (Thailand) **c***Calapnitavermiformis* (Philippines) **d***Apokayanakapit* (Sarawak) **e***Uthina* sp. n. “Ind121” (Indonesia) **f***Panjangecasaroro* (Philippines).

**Figure 11.**
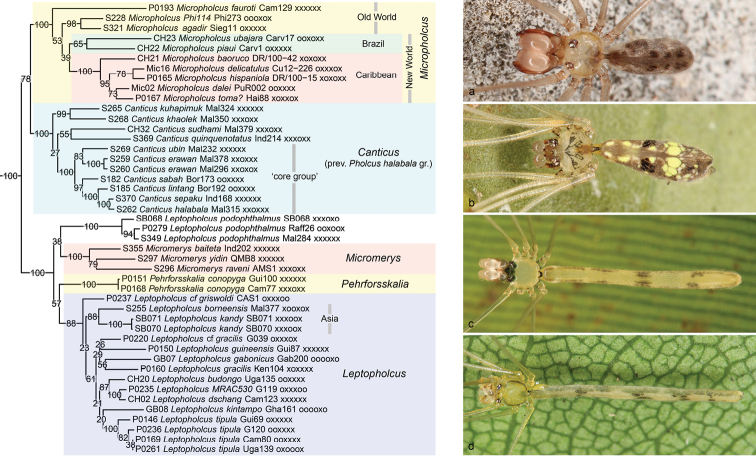
*Micropholcus*-*Leptopholcus* clade **a***Micropholcus* sp. n. “Br15-152” (Brazil) **b***Canticussepaku* (East Kalimantan) **c***Micromerysbaiteta* (West Papua) **d***Leptopholcusborneensis* (Singapore).

**Figure 12.**
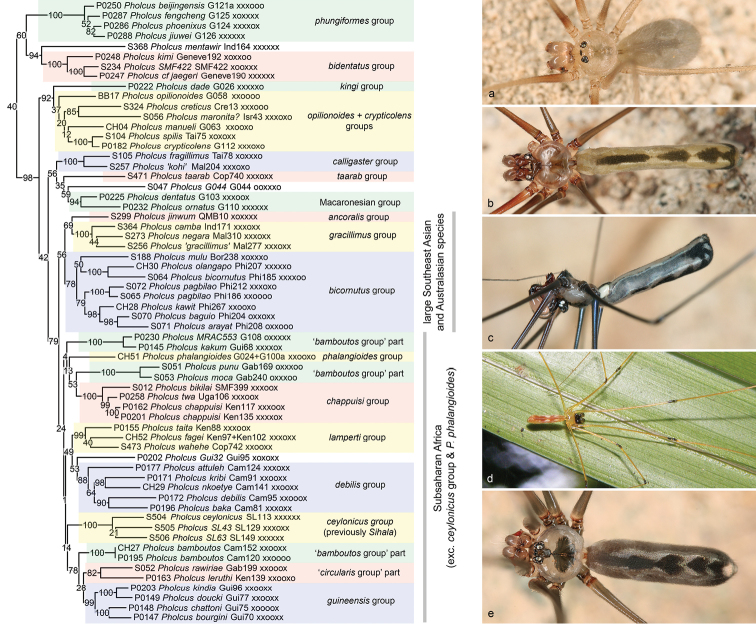
*Pholcus***a***P.creticus* (Crete) **b***P.camba* (Sulawesi) **c***P.mulu* (Sarawak) **d***P.baka* (Gabon) **e***P.* sp. n. “SL43” (Sri Lanka).

Figs 7 , 8 , 9 , 10 , 11 , 12

Pholcidae CL Koch, 1850: 31. Type genus Pholcus Walckenaer, 1805, by monotypy.
Pholcinae CL Koch; Simon 1893: 461; Huber 2011b: 218.

#### Remarks.

Pholcinae resemble Modisiminae in several respects. Their highest diversity is in the humid tropics and subtropics, and a large variety of body forms reflect adaptations to different microhabitats. With currently 922 species in 26 genera, Pholcinae is also similar to Modisiminae in diversity. In contrast to Modisiminae, Pholcinae is largely restricted to the Old World, with the notable exception of the New World endemic genus *Metagonia* Simon, 1893 and a few possibly relict species in *Pholcus* and *Micropholcus* (Huber 2011a, Huber et al. 2014). While only a single species of Modisiminae has followed humans around the globe [*Modisimusculicinus* (Simon, 1893)] and one further species has spread widely in Europe and neighboring regions [*Psilochorussimoni* (Berland, 1911)], several synanthropic species in Pholcinae have attained worldwide distributions or extended their ranges to another continent [most notably *Pholcusphalangioides* (Fuesslin, 1775); *Spermophorasenoculata* (Dugès, 1836); *Micropholcusfauroti* (Simon, 1887); *Pholcusmanueli* Gertsch, 1937].

The sister-group relationship between Pholcinae and Smeringopinae is well established (see above). The same is true for the monophyly of Pholcinae. All our analyses support this subfamily (reasonable to high support), and morphological data have also supported this group (presence of male lateral proximal cheliceral apophyses, Huber 1995, 2000; tarsus IV comb hairs in a single row, Huber and Fleckenstein 2008).

Even though Pholcinae are well represented in our analyses (317 of 597 species, i.e., 53%) internal relationships in this subfamily continue to be problematic. Several ‘basal’ nodes are poorly supported (Figure 1); in part the topology is highly sensitive to different algorithms of analysis; and some details appear dubious from the perspective of morphology. However, many details are strongly supported by morphology, including some deep nodes (e.g., the *Pholcus* group of genera); and some nodes, even though weakly supported or in conflict with morphology, provide reasonable and testable predictions for further research (e.g., the polyphyly of *Spermophora* Hentz, 1841; the close relationship of certain Sri Lankan taxa with African rather than Southeast Asian taxa; the monophyly of African *Pholcus*).

The subfamily is here divided into three operational groups, more for the sake of convenience than as a reflection of the support values they receive. Actually, support is low for all of them, but much of this division is consistent among different analyses and may well reflect real major groups. ‘Group 1’ (Figs 7, 8) is entirely composed of small six-eyed taxa, and is roughly equivalent to what was originally subsumed under the name *Spermophora*. ‘Group 2’ (Figure 9 part) is also entirely composed of six-eyed taxa and is remarkable because it places the exclusively New World genus *Metagonia* close to African and Madagascan taxa. ‘Group 3’ (Figure 9 part, Figs 10–12) includes the fully supported *Pholcus* group of genera as proposed previously (Huber 2011a) and its sister genus *Quamtana* Huber, 2003, a sister-group relationship that has also been proposed before (Huber 2003c). In the tree shown here (and in the RogueNaRok tree), the ‘*Spermophora*’ *dieke* group has an isolated position outside of the three operational groups. In the other trees, it is part of ‘group 1’.

### Pholcinae ‘group 1’

Taxon classificationAnimaliaAraneaePholcidae

Figs 7 , 8

#### Remarks.

This group includes some genera named long ago, like *Spermophora*, *Belisana*, and *Paramicromerys* Millot, 1946. Most other genera were described relatively recently and resulted either from splitting of *Spermophora* (e.g., *Spermophorides* Wunderlich, 1992; *Buitinga* Huber, 2003; *Savarna* Huber, 2005; *Khorata* Huber, 2005) or from the discovery and description of new species (*Aetana* Huber, 2005; *Wanniyala* Huber & Benjamin, 2005; *Hantu* Huber, 2016).

A Southeast Asian clade that is consistently resolved with high to full support but variably placed either inside ‘group 1’ (IQ-TREE, RogueNaRok) or outside of the three operational groups as an isolated fourth group (4+ genes, RAxML) is composed of *Aetana*, Southeast Asian ‘*Spermophora*’, and an undescribed new genus from Indonesia (“Ind206”). Morphological data have suggested a close relationship of *Aetana* with *Savarna*, *Khorata*, and *Hantu* (Huber et al. 2015). The positions of those three genera in our molecular trees are all unstable and problematic (see below). Thus, we consider it premature to conclude that the morphological data were misleading, and suggest that the positions of *Savarna*, *Khorata*, and *Hantu* need further analysis. A similar problem occurs with Southeast Asian ‘*Spermophora*’. The monophyly of the five species included receives reasonable to high support, but this group does not seem to be close to the type species *S.senoculata.* However, the position of *S.senoculata* varies strongly among analyses, and the idea that Southeast Asian taxa are in fact congeneric with *S.senoculata* (Huber 2005a) should not yet be discarded based on the present molecular data.

In *Aetana*, our analyses include 16 of 18 (89%) described species plus two undescribed species. The monophyly of the genus is highly to fully supported even though morphological support appeared weak (Huber et al. 2015). All four species groups proposed after cladistic analysis of morphological characters (Huber et al. 2015) are supported, but with different relationships among each other. Most of these relationships among species groups receive low support, but the *kiukoki* group is resolved as sister of the *omayan* group (with modest support) and this is in conflict with the results from morphology (Huber et al. 2015). The two unnamed subgroups within the *kinabalu* group and within the *omayan* group, respectively, proposed in Huber et al. (2015) are all recovered (with modest to full support).

The next clade within ‘group 1’ (Figure 7) includes three taxa whose position varies strongly among different analyses (see above): the type species of *Spermophora*, *S.senoculata*, and the Southeast Asian genera *Khorata* and *Savarna*. *Spermophorasenoculata* is alternatively resolved as sister to the African ‘*Spermophora*’ *akwamu* group (RAxML) or to the African ‘*Spermophora*’ *kyambura* Huber & Warui, 2012 (4+ genes, RogueNaRok). Its sister group is essentially unknown. As indicated above, a close relationship with Southeast Asian ‘*Spermophora*’, even though never recovered by our analyses, should not be definitely discarded. *Khorata* and *Savarna* are sister taxa in some analyses (low support; IQ-TREE, RogueNaRok), but wide apart in others. The former result is considered more plausible for two reasons: (1) morphology supports a close relationship between *Khorata* and *Savarna* (Huber et al. 2015); (2) the alternative topology (4+ genes, RAxML) places the Southeast Asian *Savarna* as sister to an East African clade.

The large Asian genus *Belisana* (Figure 8) is well represented in our analyses (30 species) but seems to suffer from rogue taxa, paralogs, and/or other unidentified problems. Only the RogueNaRok tree resolves a monophyletic *Belisana*. In other analysis, either *Hantu* (RAxML) or *Hantu* and ‘*Spermophora*’ *kyambura* are nested within *Belisana* (IQ-TREE). A close relationship between *Belisana* and *Hantu* (that is also suggested in the RogueNaRok tree) is strongly contradicted by morphology: several characters support a close relationship between *Hantu*, *Khorata*, and *Savarna* (Huber et al. 2015). We have no explanation for the position of *Hantu* in our trees. Intriguingly, *H.niah* Huber, 2016 (but not *H.kapit* Huber, 2016) was placed in a clade together with *Khorata* and *Savarna* in preliminary analyses of the present data. On the other hand, the African ‘*Spermophora*’ *kyambura* might indeed be close to *Belisana*. In fact, had it been collected in Southeast Asia, it would probably have been assigned to *Belisana*. It was tentatively assigned to *Spermophora* because African ‘*Spermophora*’ were polyphyletic anyway and because the closest known record of *Belisana* was from India, more than 5000 km east. However, the position of ‘*Spermophora*’ *kyambura* varies among analyses and should be considered unresolved.

Our sample of *Belisana* includes numerous representatives from different microhabitats (litter and leaves) and with different types of webs (‘usual’ pholcid domed sheets and highly regular ‘curtain’ webs; Figs 8e–f; see also Deeleman-Reinhold 1986a, Huber 2005b). The present data suggest multiple microhabitat shifts within *Belisana*, but note that many nodes within the genus have very low support values. These low values also impede a proper interpretation of the fact that the two species with a ‘usual’ domed web (marked with D in Figure 8) included in the analyses (*B.* “Mal77”, *B.tambligan* Huber, 2005) are not ‘basal’ but nested among species with highly regular ‘curtain’ webs (marked with R in Figure 8) [confirmed for *B.bohorok* Huber, 2005; *B.leuser* Huber, 2005; *B.* “Bor85”; *B.junkoae* (Irie, 1997); *B.sabah* Huber, 2005; BA Huber, unpubl. data].

Except for the Sri Lankan genus *Wanniyala*, all remaining taxa of Pholcinae ‘group 1’ (Figure 7) are African, Madagascan, and Mediterranean. They are grouped together but with very low support. South African and Madagascan ‘*Spermophora*’ were not available for sequencing and are thus not included in our analyses; we predict they are members of this clade. As mentioned above, some analyses (RAxML, 4+ genes) placed the East Asian genus *Savarna* within this clade; we consider this topology dubious.

A close relationship between the West African ‘*Spermophora*’ *tonkoui* group and *Wanniyala* is suggested in all our analyses, even though with low support (only SH values are consistently at 96–97). This relationship is also supported by morphology: the two taxa share a hinged procursus with a membranous process arising from the proximal part (see Huber and Benjamin 2005: fig. 7, Huber 2003b: fig. 293, Huber and Kwapong 2013: fig. 101).

The following clade (Figure 7) places the Central African ‘*Spermophora*’ *awalai* group as sister to the Macaronesian and Mediterranean genus *Spermophorides*, both together sister to the Madagascan genus *Paramicromerys*, and all together sister to an undescribed Madagascan genus (“CAS13”). Support for these relationships is modest, and the clade is different in composition in the 4+ genes tree (*Spermophorides* is missing from this analysis).

The last clade in Pholcinae ‘group 1’ is highly to fully supported in all analyses and includes the East African genus *Buitinga* and East African ‘*Spermophora*’, each with full support in all analyses. The sister group relationship between these two taxa makes sense geographically but is not evident from morphology.

### Pholcinae ‘group 2’

Taxon classificationAnimaliaAraneaePholcidae

Figure 9

#### Remarks.

This operational group (Figure 9 part, i.e., without the ‘*Spermophora*’ *dieke* group and *Quamtana*) is similar to ‘group 1’ in that it is composed entirely of six-eyed species. It is weakly supported, indicating that the exact placement of its two clades among other Pholcinae remains dubious. The two clades, however, both receive high to full support in all analyses. The first clade unites the African genera *Anansus* Huber, 2007 and *Nyikoa* Huber, 2007 with the Madagascan genus *Zatavua* Huber, 2003. In a cladistic analysis of morphological characters (Huber 2007), the group was also recovered (even though as paraphyletic) when using successive character weighting (but not when using equal character weights). The character supporting this close relationship was the proximal cheliceral apophyses that point backwards in *Anansus* just as in *Nyikoa* and *Zatavua* (Huber 2007). The idea that these genera might be ‘basal’ in Pholcinae, i.e., sister to all other Pholcinae (Huber 2003a, 2007) is not supported by our analyses, but considering the low support values at deeper nodes within the subfamily it is neither strongly contradicted.

The second clade is the New World genus *Metagonia*. The genus is species-rich (currently 85 species) and ranges from Argentina to Mexico. The monophyly of the genus has never been seriously contested, and its position among the otherwise almost exclusively Old World Pholcinae has been strongly supported before, both using morphology (Huber 2000) and molecules (Bruvo-Mađarić et al. 2005, Dimitrov et al. 2013). Our analyses include 30 species of *Metagonia*, and provide for the first time a test of the operational species groups proposed in Huber (2000: 54–55). Even though those groups were not based on formal cladistic analysis (such an analysis has not yet been performed for *Metagonia*) but on overall and specific similarities, all of them appear mostly or entirely congruent with the present analyses (the only exception is the single aberrant *M.globulosa*). They are listed here in the sequence in which they appear in Figure 9, with newly proposed informal names. (1) *taruma* group (group “3” in Huber 2000); a South American group that is here resolved as monophyletic and not as a paraphyletic ‘basal’ group as speculated before (Huber 2000); (2) *petropolis* group (no species was known in 2000); a group of litter-dwelling species restricted to the Brazilian Atlantic Forest (Huber et al. 2005b); (3) *bifida* group (group “1” in Huber 2000); this South American group includes the type species *M.bifida* Simon, 1893; all species included share a sclerotized epigynum and (except for the ‘basal’ undescribed species “G062”) a distinctively bifid abdomen; (4) *potiguar* group (not recognized in Huber 2000); this group includes cave dwelling species in Brazil (*M.potiguar* Ferreira et al. 2011) and Jamaica (*M.jamaica* Gertsch, 1986) and the aberrant litter-dwelling *M.globulosa* Huber, 2000 (which was misplaced in group “5” in Huber 2000); *rica* group (group “4” in Huber 2000); a mainly North and Central American group, possibly ranging into South America, but not including all Caribbean and Central American species as speculated previously (Huber 2000; see previous group); (5) *furcata* group (group “5” in Huber 2000, together with *M.globulosa*); includes only *M.furcata* Huber, 2000 and the undescribed species “Br09-55”; as suspected previously (Huber 2000), it is close to (sister of) the next group; (6) *delicata* group (group “2” in Huber 2000); this group is composed of very small species and ranges from Mexico to northern Argentina.

### Pholcinae ‘group 3’

Taxon classificationAnimaliaAraneaePholcidae

Figs 9 , 10 , 11 , 12

#### Remarks.

A sister-group relationship between the African genus *Quamtana* and the *Pholcus* group of genera (Figure 9) is recovered in all our analyses. Support values are low, but a morphological cladistic analysis has partly suggested the same relationship (based on a distinct sclerite connecting the genital bulb to the palpal tarsus; Huber 2003c). The monophyly of *Quamtana* is highly supported in all analyses (except for the 4+ genes analysis). It was also supported by morphological data when using character weighting (but not in the equal weights analysis; Huber 2003c).

Within *Quamtana* (Figure 9), our data suggest that there is no simple geographic pattern with respect to South African species (the large majority) versus species from other parts of Africa (marked in Figure 9). By contrast, three species groups with reasonable to full support include species from both South Africa and other regions: the South African *Q.filmeri* Huber, 2003 is sister to the Madagascan undescribed species “CAS5”; the South African *Q.vidal* Huber, 2003 and *Q.umzinto* Huber, 2003 are placed in a group with species from East and Central Africa (*Q.kabale* Huber, 2003, “Cam117”); and the group including the South African *Q.embuleni* Huber, 2003 and *Q.bonamanzi* Huber, 2003 also includes species from East and Central Africa (*Q.kitahurira* Huber, 2003, *Q.oku* Huber, 2003). We suspect that *Quamtana* was once widely distributed throughout Africa but largely replaced by more modern taxa in humid regions and extinguished in northern Africa. The Paris amber fossil *Quamtanahuberi* Penney, 2007 supports this view, but its generic assignment is uncertain (Penney 2007).

All remaining clades together (Figs 10–12) represent the *Pholcus* group of genera *sensu*Huber (2011a). This clade was first proposed in Huber & Fleckenstein (2009) based on the distinctive simplified shape of the tarsus IV comb-hairs, and later supported in a cladistic morphological analysis by an additional character (female epigynal ‘knob’) (Huber 2011a). All our analyses fully support this clade. The previous morphological analysis (Huber 2011a) identified two major problems within this clade: (1) relationships among genera were basically unresolved, resulting in large polytomies; and (2) several species groups assigned to *Pholcus* appeared more closely related to other genera. The present analysis strongly supports the polyphyly of *Pholcus* in its previous composition, and it provides for the first time a reasonable framework to redefine generic limits in this large group (currently 501 species).

The first major clade within the *Pholcus* group of genera (Figure 10) is composed of three Southeast Asian genera (*Calapnita*, *Panjange*, *Uthina* Simon, 1893) as well as several Southeast Asian and Sri Lankan species groups that were originally tentatively assigned to *Pholcus* (Huber 2011a; Huber et al. 2016a, 2016b, Huber and Dimitrov 2014). We informally call it the ‘*Calapnita*-*Panjange* clade’ because many species in this group are leaf-dwellers, and representatives of *Calapnita* and *Panjange* are particularly strongly adapted to life on green leaves. Remarkably, even some of the species collected in the leaf litter (under large dead leaves on the ground) look like leaf-dwellers rather than litter dwellers (i.e., they have long abdomens, long legs, light colouration; e.g., *Kintaqasatun* (Huber, 2011) and *K.schwendingeri* (Huber, 2011); and Malaysian representatives of *Tissahamia*, previously the *Pholcusethagala* group). Ancestral character state reconstruction suggests that the ancestor of the entire clade was leaf-dwelling (Eberle et al. 2018).

The present analyses reject the monophyly of *Calapnita* (Figure 10). A recent cladistic analysis of morphological data (Huber 2017) resolved *Calapnita* as monophyletic but with low support (< 50 using Jackknifing). On the other hand, support for the two subgroups, previously called *phyllicola* group and *vermiformis* group, is full in all analyses. The two species groups have been identified long ago (Deeleman-Reinhold 1986b), and have been supported by cladistic analysis (Huber 2017). Our analyses strongly suggest that the *vermiformis* group is closer to species previously in *Pholcus* than to the *phyllicola* group (see below). The *phyllicola* group is thus elevated to genus rank (Nipisa; see Taxonomy section below).

Within *Nipisa* (Figure 10), the internal relationships proposed previously (Huber 2017) are mostly supported even though data gaps are severe in this genus (several species with only two genes): (1) *N.lehi* (Huber, 2017) [but not *N.kubah* (Huber, 2017)] is a ‘basal’ species, i.e., sister to all other species (reasonable to high support); (2) a clade including the species with egg-sacs that have all eggs aligned in a single row (weak support, possibly because *N.kubah* is included, which is contradicted by morphology and egg-sac shape); (3) a clade including *N.semengoh* (Huber, 2011) and its sister group, characterized by the position of the tarsal organ on a turret, a serrate embolus, and the shape of the pore-plates (full support).

The relationships within ‘true’ *Calapnita* (previously *vermiformis* group) proposed in Huber (2017) are only partly supported: (1) a clade with a continuous connection between epigynal plate and ‘knob’ (all species in the present analysis except *C.bario* Huber, 2017 and *C.saluang* Huber, 2011; high support); (2) within the previous clade, a clade characterized by a prolateral process at the tip of the procursus (in the present analysis: *C.nunezae* Huber, 2017 and *C.dinagat* Huber, 2017; full support).

The present analyses also reject the monophyly of *Panjange* (Figure 10). They split the genus into two unrelated lineages, one of which is equivalent to what was previously called the *nigrifrons* group; the other is equivalent to the previous *vermiformis* + *cavicola* groups (Deeleman-Reinhold and Platnick 1986, Huber and Nuñeza 2015). Our analyses place each group with reasonable to full support in clades together with species previously assigned to *Pholcus*. A morphological cladistic analysis has recently supported the monophyly of *Panjange* based on the presence of parallel ridges ventrally on the procursus and on the reduction of the bulbal uncus (Huber and Nuñeza 2015). However, the monophyly was lost when using specific weighting parameters (implied weighting with K = 1 and K = 2), and some morphological characters do in fact support the split of *Panjange*: (1) the loss of distal cheliceral apophyses in ‘true’ *Panjange* and its closest relatives according to the present analyses; (2) the loss of an uncus in ‘true’ *Panjange* and its closest relatives according to the present analyses. The *nigrifrons* group is thus elevated to genus rank (Apokayana; see Taxonomy section below).

*Apokayana* is recovered with full support. This is remarkable considering the fact that in the morphological analysis its equivalent (the *Panjangenigrifrons* group) was supported by a single homoplastic character only (Huber and Nuñeza 2015). Within the genus, our analyses identify two subgroups with full support each. These groups do not correspond to the relationships suggested in Huber and Nuñeza (2015). In that analysis, each node was based on a single character, some of them not particularly convincing. We thus tend to prefer the present grouping even though our matrix is particularly incomplete in this genus (we did not manage to get 28S and CO1 sequences for any of the six species included).

The monophyly of ‘true’ *Panjange* (*vermiformis* + *cavicola* groups) is supported by several morphological characters (Huber and Nuñeza 2015), and receives high support in our present analyses (except for the 4+ genes analysis). The *cavicola* group (including also the two undescribed species “Ind103” and “Ind109”) was recovered as paraphyletic in Huber and Nuñeza (2015) but is here resolved as monophyletic. By contrast, the *lanthana* group which was supported by two morphological characters, one of them considered particularly strong (the unique direction of the embolus, pointing in the opposite direction of the appendix) is resolved as monophyletic only in the RogueNaRok tree; in the IQ-TREE and RAxML trees it is paraphyletic with respect to the *cavicola* group (actually, these trees suggest a basal trichotomy). Within the *lanthana* group, three species (*P.malagos* Huber, 2015; *P.casaroro* Huber, 2015; *P.camiguin* Huber, 2015) share asymmetric male pedipalps, a character that is extremely rare in spiders (Huber et al. 2007, Huber and Nuñeza 2015). This group is not recovered in any of the present analyses, where it consistently includes the symmetric *P.lanthana* Deeleman-Reinhold & Deeleman, 1983 (requiring a regain of symmetry or two origins of asymmetry). Only the sister group relationship between *P.dinagat* Huber, 2015 and *P.marilog* Huber, 2015 is strongly supported by both morphology and molecules. In conclusion, alternative topologies within the *lanthana* group are supported by seemingly strong molecular and morphological data, respectively.

Ten species groups previously assigned to *Pholcus* (in Huber 2011a, Huber et al. 2016a, 2016b) are representatives of the ‘*Calapnita*-*Panjange* clade’ (Figure 10). Of these, nine are entirely Southeast Asian; only the *ethagala* group (now *Tissahamia*) has representatives in Southeast Asia and Sri Lanka. For some of these species groups, our data provide strong evidence about the sister-group or close relatives. All of these groups are here transferred from *Pholcus* to new genera (see Taxonomy section below).

A sister-group relationship between *Kelabita* (previously the *Pholcusandulau* group) and *Apokayana* (previously the *Panjangenigrifrons* group) is fully supported in all our analyses (except for the 4+ genes tree where *Kelabita* is not represented). Both genera are restricted to Borneo and share habitus, colouration, web structure, and microhabitat (Huber and Leh Moi Ung 2016, Huber et al. 2016a).

The western Indonesian ‘*Pholcuskerinci* group’ (Huber 2011a) and the Philippine ‘*Pholcusdomingo* group’ (Huber et al. 2016b) are both fully supported, as is their sister-group relationship (Figure 10). They are joined in the new genus *Teranga* (see Taxonomy section below). Together with *Tissahamia* (previously the *Pholcusethagala* group) and with ‘true’ *Panjange* they form a clade that is recovered in all analyses with reasonable support. This clade was supported in almost exactly the same composition by morphological cladistic analysis (Huber 2011a) except that the ‘*Panjange*’ *nigrifrons*group was also included (the ‘*Pholcusdomingo* group’ was not yet known in 2011). The clade is supported by the loss of the bulbal uncus and by the loss of distal male cheliceral apophyses [in Huber 2011a, the latter character supports a more inclusive taxon (including *Leptopholcus* Simon, 1893) that is strongly rejected by the present molecular data]. *Tissahamia* consists of two subgroups, a Sri Lankan subgroup and a Southeast Asian (Malaysian Peninsula, Sumatra) subgroup. The subgroups are consistently recovered in all our analyses with modest to reasonable support, but the monophyly of the entire group is only recovered in the IQ-TREE analysis (low support). Morphological analysis recovered the group, but with varying support depending on weighting regime (Huber 2011a).

Three genera composed of species that were previously assigned to *Pholcus* are consistently placed in a highly supported clade together with ‘true’ *Calapnita* (Figure 10): *Paiwana* (previously not assigned to a group), *Muruta* (previously the *Pholcustambunan* group; Huber et al. 2016b), and *Meraha* (previously the *Pholcuskrabi* group; Huber et al. 2016b). We know of no convincing morphological synapomorphy for this group but note two interesting similarities: representatives of ‘true’ *Calapnita* and of *Meraha* share the loss of piriform gland spigots on the anterior lateral spinnerets (Huber 2011a, 2017, Huber et al. 2016b); representatives of ‘true’ *Calapnita* and of *Muruta* and *Paiwana* share the distinctive shape of the epigynum (roughly triangular, with ‘knob’ directed towards anterior; Huber et al. 2016b, Huber 2017; Huber and Dimitrov 2014).

For two further genera composed of species previously assigned to *Pholcus* the present analysis supports the monophyly but gives not clear indication about their closest relatives within the ‘*Calapnita*-*Panjange* clade’ (Figure 10): *Pribumia* (previously the *Pholcusminang* group; Huber 2011a) and *Kintaqa* (previously the *Pholcusbuatong* group; Huber et al. 2016a). All analyses except the IQ-TREE analysis place *Kintaqa* as sister to *Uthina*, but with low support. We know of no potential morphological synapomorphy that links these two groups. *Pribumia* is in our analysis represented by four species. Of these, *P.diopsis* (Simon, 1901) is never placed within the group; together with *P.atrigularis* (Simon, 1901) it is detected as a rogue taxon and excluded in the RogueNaRok tree. External relationships of *Pribumia* remain dubious. The hypothesis that the genus might be close to *Tissahamia* (previously the ‘*Pholcusethagala* group’; Huber 2011a) is supported by numerous distinctive morphological similarities but it is not supported by the present data. However, note that in our analysis *Pribumia* suffers seriously from missing data (we were not able to sequence 28S for any of the four species).

The second major clade within the *Pholcus* group of genera (Figure 11) is composed of four ‘old’ genera (*Micropholcus*; *Leptopholcus*; *Micromerys* Bradley, 1877; *Pehrforsskalia* Deeleman-Reinhold & van Harten, 2001) and *Cantikus* (previously the *Pholcushalabala* group; Huber 2011a, Huber et al. 2016a). Except for one clade of Neotropical *Micropholcus*, all representatives are Old World taxa. We informally call it the ‘*MicropholcusLeptopholcus* clade’. This clade receives full support in all our analyses, and major internal relationships are also well resolved. Three subclades are fully supported each: *Micropholcus*; *Cantikus*; and a subclade including *Leptopholcus*, *Micromerys*, and *Pehrforsskalia*. All analyses put *Micropholcus* as sister to *Cantikus*, but with modest support.

*Micropholcus* is ecologically diverse, including ground-dwelling as well as rock- and leaf-dwelling species, and together with *Pholcus* it is also the only genus with autochthonous species in both the New and Old World. Our analysis rejects the previous idea that *Micropholcus* is ‘basal’ in the *Pholcus* group of genera (i.e., in a basal trichotomy, with *Sihala* occupying the second branch and all other taxa the third branch; Huber 2011a). Within *Micropholcus*, our analyses all support a monophyletic New World clade, but with low support values (reasonable support in the 4+ genes analysis). Within the New World clade, a Caribbean clade is fully supported. A remarkable sister-group relationship that is highly supported by the present data is between the Moroccan ‘*Pholcus*’ *agadir* (now transferred to Micropholcus; see Taxonomy section below) and the undescribed Philippine species “Phi114”. Both have very limited distributions; only one further species of *Micropholcus* (other than the pantropical *M.fauroti*) is known from between Morocco and the Philippines: *M.jacominae* Deeleman-Reinhold & van Harten, 2001 from Yemen. We suspect that *Micropholcus* in the Old World has a relict distribution, just as it has been hypothesized for South American *Micropholcus* (Huber et al. 2005a, 2014).

*Cantikus* was recently revised (as ‘*Pholcus*’ *halabala* group; Huber et al. 2016a) and divided into a ‘core group’ that was supported by numerous morphological and behavioral similarities, and a group of species that were assigned to the group tentatively. This tentative assignment was based mainly on preliminary results from the present molecular analysis; a putative morphological synapomorphy for the entire genus *Cantikus* was and is not known. The present analyses fully support both the entire genus and the core group; the genus includes *C.quinquenotatus* (Thorell, 1878), making the *quinquenotatus* group proposed in Huber (2011a) obsolete; and it highly to fully supports the sister group relationship between the two rock-dwelling species *C.kuhapimuk* Huber, 2016 and *C.khaolek* Huber, 2016.

The clade including *Leptopholcus*, *Micromerys*, and *Pehrforsskalia* (Figure 11) was only partly supported in a previous cladistic analysis of morphological data (Huber 2011a): while *Leptopholcus* and *Micromerys* were consistently seen as sister taxa (with a mono- or paraphyletic *Leptopholcus*), the position of *Pehrforsskalia* varied widely. The characters supporting a close relationship among the three genera are the distal position of the lateral apophyses on the male chelicerae, and the absence of frontal cheliceral apophyses (Huber 2011a). The present analyses fully support this clade. Within the clade, ‘basal’ relationships are unresolved, essentially resulting in a tetrachotomy: (1) ‘*Leptopholcus*’ *podophthalmus* (Simon, 1893) is not clearly included in ‘true’ *Leptopholcus*. (2) The Australasian *Micromerys* receives full support in all analyses. (3) The African *Pehrforsskalia* is only represented by its type species. (4) ‘True’ *Leptopholcus* receives reasonable to full support and includes both African and Asian representatives but not the Asian *L.podophthalmus* (and its putative close relative *L.tanikawai* Irie, 1999 that is not included in our analyses). Within *Leptopholcus*, our data provide little resolution, but an Asian clade (represented by *L.borneensis* Deeleman-Reinhold, 1986 and *L.kandy* Huber, 2011) receives reasonable support. Among these four clades, *Pehrforsskalia* is the only one that does not share the distinctively serrated tip of the male palpal trochanter apophysis (Huber 2011b), suggesting that it may be sister to the other three clades.

The third and last major clade within the *Pholcus* group of genera is ‘true’ *Pholcus* (Figure 12). Support for this group is very low in the IQ-TREE analysis, which reflects the fact that one of the two basal subclades (including the *phungiformes* and *bidentatus* groups and *P.mentawir* Huber, 2011) is closer to the *Micropholcus*-*Leptopholcus* clade than to ‘true’ *Pholcus* in some analyses (RAxML, RogueNaRok). By contrast, the 4+ genes analysis recovers the monophyly of ‘true’ *Pholcus* with reasonable support, suggesting that the poor support or non-monophyly of ‘true’ *Pholcus* in some analyses may result from the many missing data in our full matrix.

Even after removing the eleven species groups that are here placed in the *CalapnitaPanjange* clade and in the *MicropholcusLeptopholcus* clade, *Pholcus* continues to be the most species rich genus in Pholcidae. It now contains 321 species, most of which are distributed in tropical and subtropical Old World regions. The only exception is the *kingi* group with ten species in the southeastern USA (Huber 2011a). Most species of *Pholcus* resemble the synanthropic type species *P.phalangioides* in being relatively large, long-legged, brown, and in having a cylindrical abdomen; most or all of these species build their webs in large sheltered spaces. However, the genus is ecologically diverse and includes small litter dwellers with relatively short legs, rock- and ground dwellers with oval abdomens, and pale leaf-dwellers with worm-shaped abdomens.

In a first effort to structure the known diversity of *Pholcus*, the genus was divided into 29 operational species groups (Huber 2011a), including 25 species groups in the ‘core group’, i.e., in ‘true’ *Pholcus*. Even though the aim was to identify monophyla, some groups were explicitly proposed as ‘waste baskets groups’ (e.g., the *bamboutos* group) or as “probably not monophyletic” (e.g., the *circularis* group). The present analysis clarifies a number of relationships, it supports several of the species groups and rejects others, and it confirms the non-monophyly of some groups as suspected. However, we acknowledge that internal relationships in *Pholcus* remain highly uncertain and need considerably more work. Our data seem to suffer from two main problems that result in variable topologies among different types of analyses: (1) Even though *Pholcus* is in our analyses represented by more species than any other genus (59), our sample is still highly incomplete, including only 18% of the described species and entirely missing seven of the previously suggested species groups (*alticeps*, *nenjukovi*, *ponticus*, *zham*, *yichengicus*, *taishan*, and *nagasakiensis* groups). (2) The percentage of missing sequences is high in *Pholcus*, partly due to the fact that we identified paralogs for 28S and 18S that we excluded, partly due to other unidentified problems.

Of the 25 operational species groups within ‘true’ *Pholcus* proposed previously (Huber 2011a), ten are supported by the present data: *phungiformes* group, *bidentatus* group, *calligaster* group, Macaronesian group, *gracillimus* group (excl. *P.mentawir*), *bicornutus* group, *chappuisi* group, *lamperti* group, *debilis* group (incl. *P.nkoetye* Huber, 2011 and *P.kribi* Huber, 2011), and *guineensis* group. Four groups are represented by single species (*taarab* group, *ancoralis* group, *phalangioides* group, *kingi* group). Seven groups are missing in the analyses (see above). For the remaining four groups, the present analyses reject the monophyly: (1) The *bamboutos* group is polyphyletic as expected and the six species in our analyses split into four parts; of these *P.kribi* is moved to the *debilis* group; *P.bamboutos* Huber, 2011 is close to the *guineensis* group; the affinities of the other four species are unclear. (2) The *circularis* group is represented by three species; of these, *P.nkoetye* is moved to the *debilis* group; *P.leruthi* Lessert, 1935 and *P.rawiriae* Huber, 2014 are sister species and close to the *guineensis* group. (3/4) The *opilionoides* and *crypticolens* groups are both rejected, but together with the North American *kingi* group they form a monophylum with reasonable to high support (except for the 4+ genes analysis) but with unknown affinities with other groups.

The present analysis identifies two major clades within ‘true’ *Pholcus* that are remarkable even though support values are low to modest. (1) A clade combining the *ancoralis*, *gracillimus*, and *bicornutus* groups is composed of large dark Southeast Asian and Australasian species; a close relationship between the *ancoralis* group and the *bicornutus* group has been suspected before, based on male ocular area modifications (Huber 2011a: 314). (2) A large clade including all Subsaharan African taxa. This clade has low bootstrap support but SH values range from 81 to 96, so we consider this a first tentative indication that tropical African *Pholcus* might form a large monophylum. The two species that disrupt this picture were both identified as rogue taxa: *P.taarab* Huber, 2011 (which is not included in the clade but is African), and *P.phalangioides* (which is included but is most probably not originally African). On the other hand, the inclusion of the Sri Lankan genus *Sihala* Huber, 2011 in this clade is plausible, even though weakly supported. Our data highly support the inclusion of *Sihala* in ‘true’ *Pholcus*, but neither morphology nor molecules seem to give an indication about its sister taxon.

##### Notes on genera not included in the present analyses

*Aucana* Huber, 2000. This Chilean genus (four species; formally including the mysterious New Caledonian *A.kaala* Huber, 2000) was previously thought to be a member of Ninetinae (Huber 2000, 2011b). However, the procursus (dorsal apophysis and corresponding ventral pocket) suggests a placement in Arteminae. Within Arteminae, it shares an exposed tarsal organ with *Chisosa* and *Nita* (Huber 2000, 2011b).

*Blancoa* Huber, 2000. A small Venezuelan genus (two species), probably member of Modisiminae (Huber 2000), but the sister group remains entirely obscure.

*Canaima* Huber, 2000. Also probably member of Modisiminae, with only two species restricted to Trinidad and Venezuela (Huber 2000). The shape of the ventral apophysis on the male palpal femur is reminiscent of the Venezuelan clade including *Mecolaesthus*, *Stenosfemuraia*, *Systenita*, and ‘true’ *Coryssocnemis*.

*Cenemus* Saaristo, 2001. A small Seychellois genus (three species), member of Smeringopinae; a morphological cladistic analysis (Huber 2012) suggested a placement in the ‘northern clade’ of Smeringopinae even though the Seychelles are geographically much closer to the ‘southern clade’.

*Enetea* Huber, 2000. A monotypic Bolivian genus, member of Ninetinae (Huber 2000); the sister group remains entirely obscure.

*Galapa* Huber, 2000. A small genus (two species) restricted to the Galapagos Islands, member of Ninetinae (Huber 2000); the sister group remains entirely obscure.

*Ossinissa* Dimitrov & Ribera, 2005. A monotypic genus from the Canary Islands, member of the *Pholcus* group of genera (Huber 2011a); the sister group is dubious, but we suspect a close relationship with other Canary Island cavernicole species in ‘true’ *Pholcus* (*P.baldiosensis* Wunderlich, 1992; *P.corniger* Dimitrov & Ribera, 2006).

*Pomboa* Huber, 2000. Member of Modisiminae, with currently four species restricted to Colombia. The vertical hairs in high density on the leg tibiae suggest an affinity to *Pisaboa* and *Waunana* (Huber 2000).

*Queliceria* González-Sponga, 2003. A monotypic Venezuelan genus, probably member of Modisiminae; the sister group remains entirely obscure.

*Tibetia* Zhang, Zhu & Song, 2006. A monotypic Chinese (Tibetan) genus, probably member of Arteminae; the sister group remains entirely obscure.

*Tolteca* Huber, 2000. A small Mexican genus (two species), member of Ninetinae. We predict that *Tolteca* is member of the North and Central American & Caribbean clade (Figure 2), together with *Pholcophora* and *Papiamenta*. The frontal humps on the male sternum and the shape of the procursus are reminiscent of *Pholcophora* (Huber 2000).

## Taxonomy

The present data suggest a large number of new undescribed genera. Twelve of them are composed entirely of undescribed new species; these will be described separately: three in Ninetinae (in our analyses: “Br15-159”, “Om6”, Ven01”); two in Arteminae (“Geneve59”, “Ind82”–“Ind96”); five in Modisiminae (“Br16-44”, MACN270”, “Br16-178” + “Br16-50”, “Br16-196”, “Br15-45”); and two in Pholcinae (“CAS13”, “Ind206”).

Other new genera will result from splitting of known genera. Of these, several receive high support but taxonomic changes will not be implemented here for various reasons:

(1) taxonomic work on these taxa is currently in progress and the formal taxonomic changes will be published in that context [‘*Holocneminus*’ *huangdi*, South American ‘*Psilochorus*’, *Holocnemuscaudatus* (Dufour, 1820)].

(2) The included species need to be restudied in order to assess the scope of the new genera and to formulate diagnoses (Central American ‘*Coryssocnemis*’).

Some potentially new genera are suggested by the present data but with low support values and/or without clear support from morphology. We suggest that these cases should be re-evaluated in detail in separate studies.

(1) For the southern clade of *Mesabolivar*, our analyses suggest two options: either to synonymize *Mesabolivar* and *Otavaloa* with *Litoporus* (resulting in a huge, very heterogeneous group), or to split *Mesabolivar*. The latter would preserve the names *Litoporus*, *Mesabolivar*, and *Otavaloa*, and possibly revalidate the name *Kaliana* Huber, 2000 (synonymized with *Mesabolivar* in Astrin et al. 2007), but possibly result in a morphologically non-diagnosable genus for the southern clade of *Mesabolivar*; *Teuia* would be an available name for this group.

(2) The *Smeringopuschogoria* and *rubrotinctus* groups together could either form a new genus, stay in *Smeringopus*, or be moved to *Smeringopina*.

(3) *Leptopholcuspodophthalmus* (and its close relative *L.tanikawai*) may or may not represent a separate genus.

For *Spermophora*, our data strongly suggest the polyphyly of the genus and possibly five or more new genera: for four African taxa (‘*Spermophora*’ *kyambura*, *tonkoui* group, *awalai* group, East African ‘*Spermophora*’) and for East Asian ‘*Spermophora*’. However, several important species groups are missing in our analyses, such as South African, Madagascan, and Middle Eastern representatives. We strongly suggest including at least those groups before deciding on how to split *Spermophora*.

For *Calapnita* and *Panjange*, morphological cladistic analyses have weakly supported the monophyly of each genus, but also the existence of two distinctive subgroups in each (Huber and Nuñeza 2015, Huber 2017). The present analyses reject the monophyly of each of the two genera. Since the present analyses also strongly support the two subgroups in each genus, we feel that the pros of splitting (monophyletic genera in the most complete available analysis of Pholcidae relationships) outweighs the cons (weak morphological support of monophyly; the two subgroups of *Calapnita* are largely indistinguishable in the field).

Finally, our data strongly support the splitting of *Pholcus*, and this is largely in agreement with previous morphological cladistic analysis (Huber 2011a). The species groups that are here formally described as new genera have all been revised recently, and the diagnosis for *Pholcus* in Huber (2011a) that was explicitly valid for the ‘core group’ only, finally applies to the entire genus.

The present data also suggest a number of synonymies and new combinations, some of which are not formalized here.

(1) *Anopsicus* appears nested within *Modisimus*. However, neither the type species of *Anopsicus* is included in our analyses nor is a potential close relative; we conclude that the monophyly and position of *Anopsicus* both remain dubious.

(2) Our data suggest that *Coryssocnemis* and *Systenita* may both be synonyms of *Mecolaesthus*, but our taxon sampling is weak, the topology is unstable (see above), and several internal nodes in the clade have low support. The morphologically very diverse genus *Mecolaesthus* and its closest relatives clearly need more work.

(2) In most of our analyses, *Hantu* is nested within *Belisana*. For reasons detailed above we strongly doubt this result.

For other synonyms and transfers, we consider the available data strong enough to justify formal changes:

(1) The Cuban endemic genus *Platnicknia* Özdikmen & Demir, 2009 is newly synonymized with *Modisimus* Simon, 1893, syn. n. Our analyses do not include the type species *P.coxana* (Bryant, 1940) but two very similar undescribed species from near the type locality of *P.coxana* (“Cu12-99” and “Cu12-100”). Our analyses strongly support a sister group relationship of *Platnicknia* with a Hispaniolan group of leaf-dwelling *Modisimus*. Both together are deeply nested within other groups of *Modisimus* (Figure 3).

(2) The Moroccan *Pholcusagadir* is nested within *Micropholcus*. This placement receives high support in our analyses, while the previous assignment to *Pholcus* (Huber 2011a) was tentative; *Micropholcusagadir* (Huber, 2011), comb. n.

(3) The southern Indian/Sri Lankan genus *Sihala* Huber, 2011 is synonymized with *Pholcus* Walckenaer, 1805, syn. n. The position of *Pholcusceylonicus* O. Pickard-Cambridge, 1869 (comb. re-established) in *Pholcus* had long been doubted (e.g., Brignoli 1972). The male genitalia of the two formally described species [*P.ceylonicus* and *P.alagarkoil* (Huber, 2011) comb. n.] are dramatically different from ‘usual’ *Pholcus* (shapes of trochanter apophysis and of femur; small and simple procursus without ventral ‘knee’; bulb without uncus, with large massive appendix; Huber and Benjamin 2005, Huber 2011a). It was thus no surprise when a morphological cladistic analysis suggested a placement far away from the core group of *Pholcus* (Huber 2011a). However, our present analyses include three species of *Sihala*, two of them without missing genes, and *Sihala* was consistently placed in ‘true’ *Pholcus*.

Finally, the two changes at the level of subfamily suggested by all or some of our analyses are not implemented:

(1) All our analyses suggest that *Artema* is an isolated genus and single representative of Arteminae and that ‘other Arteminae’ should receive a new subfamily name. For reasons detailed above we consider the position of *Artema* in our analyses dubious and do not propose a new subfamily for ‘other Arteminae’.

(2) Some of our analyses suggest an isolated position of the Andean genus *Priscula*: it may be either a ‘basal’ representative of Modisiminae or a separate subfamily. Since the relevant nodes in our analyses all receive low support values, we prefer to keep *Priscula* in Modisiminae until more convincing data become available.

### Subfamily *Pholcinae* C.L. Koch, 1850

#### Nipisa

Taxon classificationAnimaliaAraneaePholcidae

Huber gen. n.

http://zoobank.org/EB3A11CC-FE6C-4451-AA9A-8E582242C441

Calapnita phyllicola group: Deeleman-Reinhold 1986b: 212. Huber 2011a: 43. Huber 2017: 7.

##### Type species.

*Calapnitaphyllicola* Deeleman-Reinhold, 1986.

##### Etymology.

The name is derived from the Malay word *nipis* (thin), and refers to the long and thin abdomen. Gender feminine.

##### Diagnosis

(adapted from Huber 2017). Leaf-dwelling, pale whitish, long-legged pholcids with six eyes and long cylindrical abdomen (Huber 2017: figs 3-19). Distinguished from *Calapnita* by (1) tibia 2/ tibia 4 length >1.05 (*vs.* <0.95 in *Calapnita*); (2) ALS with eight spigots each (*vs.* two in *Calapnita*) (Huber 2017: figs 31, 41, 78); (3) proximal lateral processes on male chelicerae in ‘usual’ proximal position (*vs.* distal in *Calapnita*) (Huber 2017: figs 23, 35); (4) simple apophysis on male palpal trochanter (*vs.* hooked and sclerotized in *Calapnita*) (Huber 2017: figs 21, 34); (5) male palpal femur barely modified (*vs.* with series of three ventral sclerotized processes in *Calapnita*) (Huber 2017: figs 21, 34); (6) epigynum roughly rectangular or trapezoidal with folded cuticle and posterior ‘knob’ (*vs.* triangular with anterior ‘knob’ in *Calapnita*) (Huber 2017: figs 24, 32, 36, 43). For characters distinguishing *Nipisa* from similar species in other genera see Diagnosis of *Calapnita* in Huber (2017).

##### Distribution.

Southeast Asia (Huber 2017: figs 281 and 282).

##### Composition.

Ten species, all newly transferred from *Calapnita*: *N.anai* (Huber, 2017); *N.bankirai* (Huber, 2017); *N.bidayuh* (Huber, 2017); *N.deelemanae* (Huber, 2011); *N.kubah* (Huber, 2017); *N.lehi* (Huber, 2017); *N.phasmoides* (Deeleman-Reinhold, 1986); *N.phyllicola* (Deeleman-Reinhold, 1986); *N.semengoh* (Huber, 2017); *N.subphyllicola* (Deeleman-Reinhold, 1986).

#### Apokayana

Taxon classificationAnimaliaAraneaePholcidae

Huber gen. n.

http://zoobank.org/3C3E969C-F000-4596-86B9-60F6C600C2FE

Panjange nigrifrons group: Deeleman-Reinhold and Platnick 1986: 561. Huber 2011a: 109. Huber and Leh Moi Ung 2016: 3.

##### Type species.

*Panjangekapit* Huber, 2011.

##### Etymology.

Named for the Apo Kayan people, one of the Dayak people groups that are spread throughout Sarawak, East Kalimantan, and North Kalimantan. Gender feminine.

##### Diagnosis

(adapted from Huber and Leh Moi Ung 2016). Easily distinguished from *Panjange* by presence of distal male cheliceral apophyses (Huber and Leh Moi Ung 2016: fig. 18) and by ventral apophysis on male palpal femur (Huber and Leh Moi Ung 2016: fig. 30); also by absent or short epigynal scape. From representatives of *Pribumia* by ridges ventrally on procursus (Huber and Leh Moi Ung 2016: figs 17, 26, 34, 49; absent in *A.tahai*), by ventral apophysis on male palpal femur, and possibly by wide opening of palpal tarsal organ (Huber 2011a: figs 481, 686). From other similar genera on Borneo (*Calapnita*, *Leptopholcus*, *Kelabita*) by combination of: male colouration (Huber and Leh Moi Ung 2016: figs 8, 61; male ocular area and palps black; similar only in *Kelabita*), by bipartite distal apophyses on male chelicerae (Huber and Leh Moi Ung 2016: figs 35, 47; similar only in some *Calapnita*), by cylindrical rather than worm-shaped abdomen (Huber and Leh Moi Ung 2016: figs 8–15, 61–68; in contrast to *Calapnita* and *Leptopholcus*).

##### Distribution.

Borneo (Huber and Leh Moi Ung 2016: fig. 1).

##### Composition.

Ten species, all newly transferred from *Panjange*: *A.bako* (Huber, 2011); *A.iban* (Huber, 2011); *A.kapit* (Huber, 2016); *A.kubah* (Huber, 2016); *A.niah* (Huber, 2016); *A.nigrifrons* (Deeleman-Reinhold & Deeleman, 1983); *A.pueh* (Huber, 2016); *A.sedgwicki* (Deeleman-Reinhold & Platnick, 1986); *A.seowi* (Huber, 2016); *A.tahai* (Huber, 2011).

#### Pribumia

Taxon classificationAnimaliaAraneaePholcidae

Huber gen. n.

http://zoobank.org/8BC96288-E983-4EAC-8015-7A6A26F5D729

Pholcus minang group: Huber 2011a: 144.

##### Type species.

*Pholcussingalang* Huber, 2011.

##### Etymology.

The name is derived from Pribumi, a name for native Indonesians. Gender feminine.

##### Diagnosis

(adapted from Huber 2011a). Distinguished from other genera in Pholcinae by the combination of the following characters: elongate abdomen, six eyes, eye triads on stalks, male chelicerae with proximal and distal apophyses, distal apophyses ‘divided’ (consisting of two parts; Huber 2011a: figs. 640, 664), male palpal femur proximo-ventrally enlarged (Huber 2011a: figs. 628, 650), bulb with uncus, with complex sclerotized embolus, without appendix, epigynum weakly sclerotized, with small ‘knob’.

##### Distribution.

Malay Peninsula and Sumatra (Huber 2011a: fig. 626; note that *Pholcustahai* in that figure is now in *Apokayana*).

##### Composition.

The *Pholcusminang* group originally included seven species. Of these, *Pholcustahai* is now in *Apokayana* (see above); the six others are newly transferred from *Pholcus*: *P.minang* (Huber, 2011); *P.singalang* (Huber, 2011); *P.hurau*(Huber, 2011); *P.bohorok* (Huber, 2011); *P.atrigularis* (Simon, 1901); assigned tentatively: *P.diopsis* (Simon, 1901).

#### Tissahamia

Taxon classificationAnimaliaAraneaePholcidae

Huber gen. n.

http://zoobank.org/14CB661D-B5F3-4DA8-B624-F02FF0AFC21E

Pholcus ethagala group: Huber 2011a: 171.

##### Type species.

*Pholcusethagala* Huber, 2011.

##### Etymology.

Named for Wanniyalaeto chief Uru Warige Tissahami (1903–1996), who struggled (without success) against the government to keep the land of his ancestors. Gender feminine.

##### Diagnosis

(adapted from Huber 2011a). Distinguished from other genera in Pholcinae by the combination of the following characters: elongate abdomen that is slightly pointed or elevated dorso-posteriorly, six eyes, eye triads on stalks, male chelicerae with proximal apophyses in frontal position, without distal apophyses (Huber 2011a: figs. 795, 811, 816), male palpal trochanter with short retrolateral and longer ventral apophyses, palpal tarsus with dorsal elongation (except *T.phui*), bulb with large and complex appendix and weakly sclerotized embolus, without uncus, procursus highly complex, epigynum weakly sclerotized, with ‘knob’.

##### Distribution.

Sri Lanka, Malay Peninsula, and Sumatra (Huber 2011a: fig. 718 – note that *Pholcusschwendingeri* in that figure is now in *Kintaqa*; Huber et al. 2016a: fig. 1).

##### Composition.

The *Pholcusethagala* group originally included seven species. Of these, *Pholcusschwendingeri* is now in *Kintaqa* (see below); five species have been added recently, resulting in eleven species, all newly transferred from *Pholcus*: *T.ethagala* (Huber, 2011); *T.kottawagamaensis* (Yao & Li, 2016); *T.maturata* (Huber, 2011). Assigned tentatively: *T.barisan* (Huber, 2016); *T.bukittimah* (Huber, 2016); *T.gombak* (Huber, 2011); *T.ledang* (Huber, 2011); *T.phui* (Huber, 2011); *T.tanahrata* (Huber, 2016); *T.uludong* (Huber, 2016); *T.vescula* (Simon, 1901).

#### Teranga

Taxon classificationAnimaliaAraneaePholcidae

Huber gen. n.

http://zoobank.org/E67A0726-CF19-4CE3-ADFE-2D903D2778CB

Pholcus kerinci group: Huber 2011a: 166.
Pholcus domingo group: Huber et al. 2016b: 34.

##### Type species.

*Pholcuskerinci* Huber, 2011.

##### Etymology.

The name is derived from the Malay word *terang* (bright, light), and refers to the light colouration of the spiders. Gender feminine.

##### Diagnosis.

Medium-sized, long-legged spiders (body length ~3.5–4.5, leg 1:~30–40) with slender elongate abdomen that is slightly elevated posteriorly (Huber 2011a: figs 606-609, Huber et al. 2016b: figs 131–139). Easily distinguished from similar relatives in other genera (*Panjange*, *Tissahamia*, *Apokayana*, *Paiwana*) by unmodified or barely modified male chelicerae Huber 2011a: fig. 722; Huber et al. 2016b: fig. 142); also by combination of: eight eyes, triads only slightly elevated; male palpal trochanter with long ventral apophysis (Huber 2011a: figs 720, 737; Huber et al. 2016b: figs 141, 158); male genital bulb without uncus but with massive appendix (Huber 2011a: figs 719, 736; Huber et al. 2016b: figs 140, 150, 157); epigynum weakly sclerotized, with numerous transversal folds, with ‘knob’ (Huber 2011a: figs 733, 739; Huber et al. 2016b: figs 143, 156, 159).

##### Distribution.

Known from Indonesia (Sumatra, Java) and the Philippines (Mindanao) (Huber 2011a: fig. 718, Huber et al. 2016b: fig. 1).

##### Composition.

The genus includes the four species originally described in the *Pholcuskerinci* and *domingo* groups. They are all newly transferred from *Pholcus*: *T.cibodas* (Huber, 2011); *T.domingo* (Huber, 2016), *T.kerinci* (Huber, 2011); *T.matutum* (Huber, 2016).

#### Paiwana

Taxon classificationAnimaliaAraneaePholcidae

Huber gen. n.

http://zoobank.org/BAFDACD1-7142-4909-98E0-359BDC812698

##### Type species.

*Pholcuspingtung* Huber & Dimitrov, 2014.

##### Etymology.

Named for the Paiwan, an indigenous people of Taiwan. Gender feminine.

##### Diagnosis.

Large, long-legged spiders with six eyes and cylindrical abdomen (Huber and Dimitrov 2014: figs 1–4). Easily distinguished from similar species in other genera (*Teranga*, *Pholcus*, *Muruta*) by unique modifications of male chelicerae (pair of weakly sclerotized lateral apophyses and two pairs of distinctive frontal apophyses: proximal pair flat and pointed, distal pair finger-shaped, both without modified hairs; Huber and Dimitrov 2014: fig. 19); from most genera (except *Muruta*, *Calapnita*) also by shape of epigynum (roughly triangular plate, ‘knob’ directed towards anterior) (Huber and Dimitrov 2014: fig. 20).

##### Distribution.

Taiwan (Huber and Dimitrov 2014: fig. 34).

##### Composition.

Only two species newly transferred from *Pholcus*: *P.chengpoi* (Huber & Dimitrov, 2014); *P.pingtung* (Huber & Dimitrov, 2014).

#### Muruta

Taxon classificationAnimaliaAraneaePholcidae

Huber gen. n.

http://zoobank.org/E06DDC46-92E6-4740-BFCA-B2AD7807B9D5

Pholcus tambunan group: Huber et al. 2016b: 25.

##### Type species.

*Pholcustambunan* Huber, 2016.

##### Etymology.

Named for the Murut, an indigenous ethnic group inhabiting northern inland regions of Borneo. Gender feminine.

##### Diagnosis

(adapted from Huber et al. 2016b). The two species included in this genus are medium-sized, long-legged spiders (body length ~4, male leg 1 length: ~35–40), distinguished from other genera in Pholcinae by the combination of the following characters: elongate abdomen angular dorso-posteriorly (Huber et al. 2016b: figs 94, 98); six eyes; male chelicerae with distinctive distal apophyses (flat sclerites without modified hairs; Huber et al. 2016b: figs 103, 108, 120); most palpal structures unusually long (in particular genital bulb; Huber et al. 2016b: figs 101, 121); male bulb without uncus; epigynum weakly sclerotized, scape directed towards anterior with terminal ‘knob’ (Huber et al. 2016b: figs 104, 123); female internal genitalia with pair of highly distinctive three-layered telescopic tubes (Huber et al. 2016b: figs 105, 124).

##### Distribution.

Northern Borneo (Huber et al. 2016b: fig. 1).

##### Composition.

Only two species newly transferred from *Pholcus*: *M.tambunan* (Huber, 2016); *M.bario* (Huber, 2016).

#### Meraha

Taxon classificationAnimaliaAraneaePholcidae

Huber gen. n.

http://zoobank.org/14C88D64-C69D-4AB6-810B-45D93560B816

Pholcus krabi group: Huber et al. 2016a: 30.

##### Type species.

*Pholcuskrabi* Huber, 2016.

##### Etymology.

The name is derived from the Malay word *merah* (red), and refers to the red or orange colour of the male pedipalps. Gender feminine.

##### Diagnosis.

Medium size, light coloured pholcids with long legs and cylindrical abdomen (Huber et al. 2016a: figs 102-109); distinguished from similar species in other genera (*Kelabita*, *Apokayana*, *Teranga*, *Muruta*) by combination of: six eyes; absence of modified hairs on distal male cheliceral apophyses (Huber et al. 2016a: fig. 118); reduction of ALS spigots to two (Huber 2011a: fig. 566, Huber et al. 2016a: fig. 122); reddish to orange male palps (Huber et al. 2016a: figs 102, 106, 108). In the field they can be distinguished from most other genera (except *Kelabita*) by their domed webs relatively high among the vegetation (0.5–2 m above the ground), usually with the apex of the dome attached to the underside of a leaf.

##### Distribution.

Mainland Southeast Asia and Borneo (Huber et al. 2016a: fig. 110).

##### Composition.

Seven species newly transferred from *Pholcus*: *M.chiangdao* (Huber, 2011); *M.khene* (Huber, 2011); *M.kinabalu* (Huber, 2011); *M.kipungit* (Huber, 2016); *M.krabi* (Huber, 2016); *M.narathiwat* (Huber, 2016); *M.shuye* (Yao & Li, 2017).

#### Kelabita

Taxon classificationAnimaliaAraneaePholcidae

Huber gen. n.

http://zoobank.org/D88513D8-B25E-4CE6-94D6-B7BF215B2AA7

Pholcus andulau group: Huber et al. 2016a: 47.

##### Type species.

*Pholcusandulau* Huber, 2011.

##### Etymology.

Named for the Kelabit, an indigenous Dayak people of the Sarawak/North Kalimantan highlands of Borneo with a minority in the neighboring state of Brunei. Gender feminine.

##### Diagnosis.

Medium size, light coloured pholcids with long legs, six eyes, cylindrical abdomen (Huber et al. 2016a: figs 193–196). Distinguished from similar species in other genera (*Meraha*, *Apokayana*, *Teranga*, *Muruta*) by unique, partly sclerotized embolus with strong sclerotized pointed processes (Huber 2011a: fig. 570; Huber et al. 2016a: figs 200, 210); also by combination of: male chelicerae with pair of pointed apophyses close to median line and directed toward each other (Huber 2011a: fig. 572; Huber et al. 2016a: fig. 202); ALS with eight spigots each (Huber 2016a: figs 217, 218); male palps not reddish or orange; large unsclerotized ‘knob’ on posterior edge of female external genitalia, directed toward anterior (Huber 2011a: fig. 573; Huber et al. 2016a: figs 203, 213). In the field they can be distinguished from most other genera (except *Meraha*) by their domed webs among the vegetation (up to 2 m above the ground), usually with the apex of the dome attached to the underside of a leaf.

##### Distribution.

Northern Borneo (Huber et al. 2016a: fig. 153).

##### Composition.

Only two species newly transferred from *Pholcus*: *K.andulau* (Huber, 2011); *K.lambir* (Huber, 2016).

#### Kintaqa

Taxon classificationAnimaliaAraneaePholcidae

Huber gen. n.

http://zoobank.org/F4C48066-1FEC-4E3B-958F-359242174F1B

Pholcus buatong group: Huber et al. 2016a: 38.

##### Type species.

*Pholcusbuatong* Huber, 2011.

##### Etymology.

The name honours the Kintaq, a Mon-Khmer ethnic group in Thailand. Gender feminine.

##### Diagnosis.

Medium size, light coloured pholcids with long legs, six or eight eyes, and cylindrical abdomen (Huber et al. 2016a: figs 143–152). Distinguished from similar species in other genera (*Tissahamia*, *Cantikus*, *Pribumia*) by distinctive dorsal bulging of male palpal patella (Huber 2011a: figs 581, 823; Huber et al. 2016a: fig. 155) and by epigynum with large, heavily sclerotized ‘knob’ (Huber et al. 2016a: figs 184, 187, 190); also by combination of: complete reduction of distal anterior apophyses on male chelicerae (Huber 2011a: figs 582, 825; Huber et al. 2016a: fig. 156); ALS with eight spigots each (Huber et al. 2016a: figs 166, 183); male palps not reddish or orange.

##### Distribution.

Southern Thailand and northern mainland Malaysia (Huber et al. 2016a: fig. 153).

##### Composition.

Five species, all newly transferred from *Pholcus*: *K.buatong* (Huber, 2016); *K.fuza* (Yao & Li, 2017); *K.mueangensis* (Yao & Li, 2017); *K.satun* (Huber, 2011); *K.schwendingeri* (Huber, 2011).

#### Cantikus

Taxon classificationAnimaliaAraneaePholcidae

Huber gen. n.

http://zoobank.org/A71947B6-1279-4F84-8DB7-9B037D1BC70B

Pholcus halabala group: Huber 2011a: 126. Huber et al. 2016a: 3.
Pholcus quinquenotatus group: Huber 2011a: 290.

##### Type species.

*Pholcushalabala* Huber, 2016.

##### Etymology.

The name is derived from the Malay word *cantik* (beautiful), and refers to the colour patterns on the abdomen of several species. Gender masculine.

##### Diagnosis

(adapted from Huber et al. 2016a): The core group of eight species (see below) includes medium-sized, long-legged spiders (body length ~3–4, male leg 1 length ~30–40); distinguished from other genera in Pholcinae by the combination of the following characters: elongate abdomen pointed dorso-posteriorly, with distinctive dorsal pattern of black and whitish or yellowish marks in life specimens (Huber et al. 2016a: figs 1–16); eight eyes; male ocular area with conspicuous modified hairs (setae), which may appear as stiff bristles or stout curved spines, or both (Huber et al. 2016a: figs 19, 23, 43); male chelicerae with proximal and distal apophyses, distal apophyses with two cone-shaped teeth (modified hairs) each (Huber et al. 2016a: fig. 28); male bulb with uncus and appendix; procursus with distinctive dorsal flap (Huber et al. 2016a: fig. 35; absent in *C.erawan*); epigynum weakly sclerotized, with ‘knob’.

##### Distribution.

Widely distributed in Southeast Asia, from Myanmar and southern China to Sumatra, Borneo, and Bali.

##### Composition.

27 species, all newly transferred from *Pholcus*: *C.anaiensis* (Yao & Li, 2016); *C.erawan* (Huber, 2011); *C.halabala* (Huber, 2011); *C.lintang* (Huber, 2016); *C.sabah* (Huber, 2011); *C.sepaku* (Huber, 2011); *C.ubin* (Huber, 2016); *C.zhuchuandiani* (Yao & Li, 2016).

Assigned tentatively. *C.ballarini* (Yao & Li, 2016); *C.cheni* (Yao & Li, 2017); *C.chiangmaiensis* (Yao & Li, 2016); *C.elongatus* (Yin & Wang, 1981); *C.exceptus* (Tong & Li, 2009); *C.gou* (Yao & Li, 2016); *C.khaolek* (Huber, 2016); *C.kuhapimuk* (Huber, 2016); *C.namou* (Huber, 2011); *C.pakse* (Huber, 2011); *C.phami* (Yao, Pham & Li, 2015); *C.pyu* (Huber, 2011); *C.quinquenotatus* (Thorell, 1878); *C.subwan* (Yao & Li, 2017); *C.sudhami* (Huber, 2011); *C.taptaoensis* (Yao & Li, 2016); *C.tharnlodensis* (Yao & Li, 2016); *C.wan* (Yao & Li, 2016); *C.youngae* (Huber, 2011).

## Outlook

Even though the present tree of Pholcidae is a significant step forward in terms of comprehensiveness and resolution, we have identified above many weak points and aspects that need further study. Here we list a subjective ‘top-ten’ selection of projects that in our view might fill the most obvious gaps and provide the most valuable next steps.

1. Ninetinae external and internal relationships. The poorly known Ninetinae seem to differ from ‘typical’ pholcids in many respects, including body size and proportions, diversity, ecological requirements, and probably also biology. Ninetinae might be sister to all other pholcids and might have retained ancestral character states. Resolving external and internal relationships of Ninetinae is thus of particular interest but will probably require a genome-scale phylogenetic approach.

2. Position of *Artema*. Our analyses suggest an isolated position of *Artema*, not within or as sister to other Arteminae. We question this result but cannot explain it. Resolving the position of *Artema* will probably need a genome-scale phylogenetic approach.

3. Position of *Priscula*. The mysterious Andean genus *Priscula* is similar to *Artema* in including some of the largest pholcids and in defying placement in the phylogeny. As for *Artema*, a genome-scale phylogenetic approach will probably be necessary to resolve its position.

4. Andean Modisiminae. Most Pholcidae from anywhere in the world can now be quickly and reliably assigned to an existing genus. The only major exception is Modisiminae from northwestern South America, in particular Peru, Ecuador, Colombia, and Venezuela. Our analyses include a minimal sample of species from this megadiverse region that is still relatively poorly explored even at generic level.

5. Monophyly and position of *Anopsicus*. Our analyses suggest that *Anopsicus* might just be a group of dwarfed ground-dwelling *Modisimus*. However, our sample includes only three species of *Anopsicus* and none of them appears close to the type species. A much larger sample of this species-rich genus will thus be necessary to evaluate its monophyly and phylogenetic position.

6. *Holocnemus*. The type species of *Holocnemus*, *H.pluchei*, was excluded from our dataset because its position was drastically unstable in preliminary analyses. The two other species of *Holocnemus* are both included but do not group together. We suggest a genome-scale phylogenetic approach, including the three species of *Holocnemus* together with representatives of *Hoplopholcus*, *Stygopholcus*, and *Crossopriza* to solve this problem.

7. *Spermophora*. Even though many species originally described as *Spermophora* have been transferred to other or new genera, the genus continues to be polyphyletic. Our analyses suggest that five or more genera may need to be created to account for the relationships among the included species. A reanalysis of *Spermophora* should focus on including South African and Madagascan taxa as well as Middle Eastern taxa that we predict are the closest relatives of the type species *S.senoculata.*

8. *Belisana*. *Belisana* is particularly interesting for including representatives in different microhabitats and with different types of webs. However, our sample of species is limited, web data are available for relatively few species, and several nodes in our tree have low support. Thus, a much denser sampling combined with field observations will be necessary to reconstruct microhabitat shifts and the evolution of web designs within *Belisana*.

9. *Pholcus*. Our sample includes only 18% of the described species of *Pholcus* and several species groups are entirely missing. As a result, internal relationships of this largest genus in the family remain highly uncertain and need considerably more study.

10. Missing genera. The eleven described genera that are missing from our analyses contain a total of only 24 known species, but some of them are of particular interest and should be added in future analyses. (1) *Aucana*, originally described as a Ninetinae genus, is predicted to be a member of Arteminae. (2) *Cenemus* is geographically closer to the ‘southern clade’ of Smeringopinae, but predicted to be a member of the ‘northern clade’. (3) *Ossinissa*, possibly a close relative of cavernicole ‘true’ Macaronesian *Pholcus*, and thus a generic synonym. (4) *Tibetia*, probably member of Arteminae, possibly a dwarfed *Artema*. (5) *Tolteca*, predicted to be a member of the North and Central American and Caribbean clade of Ninetinae.

## Supplementary Material

XML Treatment for Ninetinae

XML Treatment for Arteminae

XML Treatment for Modisiminae

XML Treatment for Smeringopinae

XML Treatment for Pholcinae

XML Treatment for Pholcinae ‘group 1’

XML Treatment for Pholcinae ‘group 2’

XML Treatment for Pholcinae ‘group 3’

XML Treatment for Nipisa

XML Treatment for Apokayana

XML Treatment for Pribumia

XML Treatment for Tissahamia

XML Treatment for Teranga

XML Treatment for Paiwana

XML Treatment for Muruta

XML Treatment for Meraha

XML Treatment for Kelabita

XML Treatment for Kintaqa

XML Treatment for Cantikus

## Appendix 1. Summary of formal taxonomic acts, in alphabetical order.

*Apokayana* Huber, gen. n.; all species newly transferred from *Panjange*

*Apokayanabako* (Huber, 2011), comb. n.

*Apokayanaiban* (Huber, 2011), comb. n.

*Apokayanakapit* (Huber, 2016), comb. n.

*Apokayanakubah* (Huber, 2016), comb. n.

*Apokayananiah* (Huber, 2016), comb. n.

*Apokayananigrifrons* (Deeleman-Reinhold & Deeleman, 1983), comb. n.

*Apokayanapueh* (Huber, 2016), comb. n.

*Apokayanasedgwicki* (Deeleman-Reinhold & Platnick, 1986), comb. n.

*Apokayanaseowi* (Huber, 2016), comb. n.

*Apokayanatahai* (Huber, 2011), comb. n.

*Cantikus* Huber, gen. n.; all species newly transferred from *Pholcus*

*Cantikusanaiensis* (Yao & Li, 2016), comb. n.

*Cantikusballarini* (Yao & Li, 2016), comb. n.

*Cantikuscheni* (Yao & Li, 2017), comb. n.

*Cantikuschiangmaiensis* (Yao & Li, 2016), comb. n.

*Cantikuselongatus* (Yin & Wang, 1981), comb. n.

*Cantikuserawan* (Huber, 2011), comb. n.

*Cantikusexceptus* (Tong & Li, 2009), comb. n.

*Cantikusgou* (Yao & Li, 2016), comb. n.

*Cantikushalabala* (Huber, 2011), comb. n.

*Cantikuskhaolek* (Huber, 2016), comb. n.

*Cantikuskuhapimuk* (Huber, 2016), comb. n.

*Cantikuslintang* (Huber, 2016), comb. n.

*Cantikusnamou* (Huber, 2011), comb. n.

*Cantikuspakse* (Huber, 2011), comb. n.

*Cantikusphami* (Yao, Pham & Li, 2015), comb. n.

*Cantikuspyu* (Huber, 2011), comb. n.

*Cantikusquinquenotatus* (Thorell, 1878), comb. n.

*Cantikussabah* (Huber, 2011), comb. n.

*Cantikussepaku* (Huber, 2011), comb. n.

*Cantikussubwan* (Yao & Li, 2017), comb. n.

*Cantikussudhami* (Huber, 2011), comb. n.

*Cantikustaptaoensis* (Yao & Li, 2016), comb. n.

*Cantikustharnlodensis* (Yao & Li, 2016), comb. n.

*Cantikusubin* (Huber, 2016), comb. n.

*Cantikuswan* (Yao & Li, 2016), comb. n.

*Cantikusyoungae* (Huber, 2011), comb. n.

*Cantikuszhuchuandiani* (Yao & Li, 2016), comb. n.

*Kelabita* Huber, gen. n.; all species newly transferred from *Pholcus*

*Kelabitaandulau* (Huber, 2011), comb. n.

*Kelabitalambir* (Huber, 2016), comb. n.

*Kintaqa* Huber, gen. n.; all species newly transferred from *Pholcus*

*Kintaqabuatong* (Huber, 2016), comb. n.

*Kintaqafuza* (Yao & Li, 2017), comb. n.

*Kintaqamueangensis* (Yao & Li, 2017), comb. n.

*Kintaqasatun* (Huber, 2011), comb. n.

*Kintaqaschwendingeri* (Huber, 2011), comb. n.

*Meraha* Huber, gen. n.; all species newly transferred from *Pholcus*

*Merahachiangdao* (Huber, 2011), comb. n.

*Merahakhene* (Huber, 2011), comb. n.

*Merahakinabalu* (Huber, 2011), comb. n.

*Merahakipungit* (Huber, 2016), comb. n.

*Merahakrabi* (Huber, 2016), comb. n.

*Merahanarathiwat* (Huber, 2016), comb. n.

*Merahashuye* (Yao & Li, 2017), comb. n.

*Micropholcusagadir* (Huber, 2011), comb. n., transferred from *Pholcus*

*Modisimuscoxanus* (Bryant, 1940), comb. n., newly transferred from *Platnicknia*

*Modisimusincertus* (Bryant, 1940), comb. n., newly transferred from *Platnicknia*

*Muruta* Huber, gen. n.; all species newly transferred from *Pholcus*

*Murutabario* (Huber, 2016), comb. n.

*Murutatambunan* (Huber, 2016), comb. n.

*Nipisa* Huber, gen. n.; all species newly transferred from *Calapnita*

*Nipisaanai* (Huber, 2017), comb. n.

*Nipisabankirai* (Huber, 2017), comb. n.

*Nipisabidayuh* (Huber, 2017), comb. n.

*Nipisadeelemanae* (Huber, 2011), comb. n.

*Nipisakubah* (Huber, 2017), comb. n.

*Nipisalehi* (Huber, 2017), comb. n.

*Nipisaphasmoides* (Deeleman-Reinhold, 1986), comb. n.

*Nipisaphyllicola* (Deeleman-Reinhold, 1986), comb. n.

*Nipisasemengoh* (Huber, 2017), comb. n.

*Nipisasubphyllicola* (Deeleman-Reinhold, 1986), comb. n.

*Paiwana* Huber gen. n.; all species newly transferred from *Pholcus*

*Paiwanachengpoi* (Huber & Dimitrov, 2014), comb. n.

*Paiwanapingtung* (Huber & Dimitrov, 2014), comb. n.

*Pholcusalagarkoil* (Huber, 2011), comb. n., newly transferred from *Sihala*

*Pholcusceylonicus* O. Pickard-Cambridge, 1869, comb. re-established, transferred from *Sihala*

*Platnicknia* Özdikmen & Demir, 2009 = *Modisimus* Simon, 1893, syn. n.

*Pribumia* Huber, gen. n.; all species newly transferred from *Pholcus*

*Pribumiaatrigularis* (Simon, 1901), comb. n.

*Pribumiabohorok* (Huber, 2011), comb. n.

*Pribumiadiopsis* (Simon, 1901), comb. n.

*Pribumiahurau* (Huber, 2011), comb. n.

*Pribumiaminang* (Huber, 2011), comb. n.

*Pribumiasingalang* (Huber, 2011), comb. n.

*Sihala* Huber, 2011 = *Pholcus* Walckenaer, 1805, syn. n.

*Teranga* Huber gen. n.; all species newly transferred from *Pholcus*

*Terangacibodas* (Huber, 2011), comb. n.

*Terangadomingo* (Huber, 2016), comb. n.

*Terangakerinci* (Huber, 2011), comb. n.

*Terangamatutum* (Huber, 2016), comb. n.

*Tissahamia* Huber gen. n.; all species newly transferred from *Pholcus*

*Tissahamiabarisan* (Huber, 2016), comb. n.

*Tissahamiabukittimah* (Huber, 2016), comb. n.

*Tissahamiaethagala* (Huber, 2011), comb. n.

*Tissahamiagombak* (Huber, 2011), comb. n.

*Tissahamiakottawagamaensis* (Yao & Li, 2016), comb. n.

*Tissahamialedang* (Huber, 2011), comb. n.

*Tissahamiamaturata* (Huber, 2011), comb. n.

*Tissahamiaphui* (Huber, 2011), comb. n.

*Tissahamiatanahrata* (Huber, 2016), comb. n.

*Tissahamiauludong* (Huber, 2016), comb. n.

*Tissahamiavescula* (Simon, 1901), comb. n.

## Supplementary materials

Supplementary material 1 Figure S1. Maximum-likelihood tree of the complete set of taxa inferred with IQ-TREE Data type: molecular data Explanation note: This tree is identical to the one shown in Figs 2–12 except that it includes all outgroups and all support values are shown (in the sequence SBS / RBS / SH-like aLRT). This is an open access article distributed under the terms of the Creative Commons Attribution License (CC BY 4.0), which permits unrestricted use, distribution, and reproduction in any medium, provided the original author and source are credited.Bernhard A. Huber, Jonas Eberle, Dimitar Dimitrov

Supplementary material 2 Figure S2. Maximum-likelihood tree of the complete set of taxa inferred with RAxML Data type: molecular data Explanation note: Support values as in Figure S1. This is an open access article distributed under the terms of the Creative Commons Attribution License (CC BY 4.0), which permits unrestricted use, distribution, and reproduction in any medium, provided the original author and source are credited.Bernhard A. Huber, Jonas Eberle, Dimitar Dimitrov

Supplementary material 3 Figure S3. Maximum-likelihood tree (RAxML) of a reduced set of taxa (excluding rogue taxa with RogueNaRok) Data type: molecular data Explanation note: In the text we refer to this tree as ‘RogueNaRok tree’. Support values: RBS. This is an open access article distributed under the terms of the Creative Commons Attribution License (CC BY 4.0), which permits unrestricted use, distribution, and reproduction in any medium, provided the original author and source are credited.Bernhard A. Huber, Jonas Eberle, Dimitar Dimitrov

Supplementary material 4 Figure S4. Maximum-likelihood tree (RAxML) of a reduced set of taxa (excluding taxa for which less than four genes were available) Data type: molecular data Explanation note: In the text we refer to this tree as ‘4+ genes tree’. Support values as in Figure S1. This is an open access article distributed under the terms of the Creative Commons Attribution License (CC BY 4.0), which permits unrestricted use, distribution, and reproduction in any medium, provided the original author and source are credited.Bernhard A. Huber, Jonas Eberle, Dimitar Dimitrov

## References

[B1] Aberer AJ Krompass D Stamatakis A (2013) Pruning rogue taxa improves phylogenetic accuracy: an efficient algorithm and webservice. Systematic Biology 62: 162–166. 10.1093/sysbio/sys078 22962004 PMC3526802

[B2] Aharon S Huber BA Gavish-Regev E (2017) Daddy-long-leg giants: Revision of the spider genus *Artema* Walckenaer, 1837 (Araneae, Pholcidae). European Journal of Taxonomy 376: 1–57. 10.5852/ejt.2017.376

[B3] Anisimova M Gil M Dufayard JF Dessimoz C Gascuel O (2011) Survey of branch support methods demonstrates accuracy, power, and robustness of fast likelihood-based approximation schemes. Systematic Biology 60: 685–699. 10.1093/sysbio/syr041 21540409 PMC3158332

[B4] Astrin JJ Misof B Huber BA (2007) The pitfalls of exaggeration: molecular and morphological evidence suggests *Kaliana* is a synonym of *Mesabolivar* (Araneae: Pholcidae). Zootaxa 1646: 17–30.

[B5] Brignoli PM (1971) Beitrag zur Kenntnis der mediterranen Pholcidae (Arachnida, Araneae). Mitteilungen des Zoologischen Museums Berlin 47(2): 255–267. 10.1002/mmnz.19710470203

[B6] Brignoli PM (1972) Ragni di Ceylon I. Missione biospeleologica Aellen-Strinati (1970) (Arachnida, Araneae). Revue suisse de Zoologie 79(2): 907–929. 10.5962/bhl.part.97143

[B7] Brignoli PM (1976) Ragni di Grecia IX. Specie nuove o interessanti delle famiglie Leptonetidae, Dysderidae, Pholcidae ed Agelenidae (Araneae). Revue suisse de Zoologie 83(3): 539–578.

[B8] Brignoli PM (1979) Spiders from Lebanon, V. On *Hoplopholcuscecconii* Kulczynski, 1908 (Pholcidae). Bulletin of the British arachnological Society 4(8): 350–352.

[B9] Brignoli PM (1981) Studies on the Pholcidae, I. Notes on the genera *Artema* and *Physocyclus* (Araneae). Bulletin of the American Museum of Natural History 170(1): 90–100

[B10] Bruvo-Mađarić B Huber BA Steinacher A Pass G (2005) Phylogeny of pholcid spiders (Araneae: Pholcidae): combined analysis using morphology and molecules. Molecular Phylogenetics and Evolution 37: 661–673. 10.1016/j.ympev.2005.08.016 16242969

[B11] Deeleman-Reinhold CL (1986a) Leaf-dwelling Pholcidae in Indo-Australian rain forests. In: Eberhard WG Lubin YD Robinson BC (Eds) Proceedings of the Ninth International Congress of Arachnology (Panama), 1983.Smithsonian Tropical Research Institute, Balboa, Republic of Panama, 45–48.

[B12] Deeleman-Reinhold CL (1986b) Studies on tropical Pholcidae II. Redescription of *Micromerysgracilis* Bradley and *Calapnitavermiformis* Simon (Araneae, Pholcidae) and description of some related new species. Memoirs of the Queensland Museum 22(2): 205–224.

[B13] Deeleman-Reinhold CL Platnick NI (1986) A new *Panjange* from northern Borneo (Araneae, Pholcidae). Journal of the New York Entomological Society 94(4): 559–561.

[B14] Dimitrov D Astrin JJ Huber BA (2013) Pholcid spider molecular systematics revisited, with new insights into the biogeography and the evolution of the group. Cladistics 29: 132–146. 10.1111/j.1096-0031.2012.00419.x 34814376

[B15] Eberle J Dimitrov D Valdez-Mondragón A Huber BA (2018) Microhabitat change drives diversification in pholcid spiders. BMC Evolutionary Biology 18: 141. 10.1186/s12862-018-1244-8 PMC6145181 30231864

[B16] Gertsch WJ (1971) A report on some Mexican cave spiders. Association for Mexican Cave Studies, Bulletin 4: 47–111.

[B17] Gertsch WJ (1982) The spider genera *Pholcophora* and *Anopsicus* (Araneae, Pholcidae) in North America, Central America and the West Indies. Association for Mexican Cave Studies, Bulletin 8: 95–144 / Texas Memorial Museum, Bulletin 28: 95–144.

[B18] Graybeal A (1998) Is it better to add taxa or characters to a difficult phylogenetic problem? Systematic Biology 47: 9–17. 10.1080/106351598260996 12064243

[B19] Guindon S Dufayard J-F Lefort V Anisimova M Hordijk W Gascuel O (2010) New algorithms and methods to estimate maximum-likelihood phylogenies: assessing the performance of PhyML 3.0. Systematic Biology 59: 307–321. 10.1093/sysbio/syq010 20525638

[B20] Heath T Hedtke S Hillis D (2008) Taxon sampling and the accuracy of phylogenetic analyses. Journal of Systematics and Evolution 46: 239–57. 10.3724/SP.J.1002.2008.08016

[B21] Huber BA (1995) Copulatory mechanism in *Holocnemuspluchei* and *Pholcusopilionoides*, with notes on male cheliceral apophyses and stridulatory organs in Pholcidae (Araneae). Acta Zoologica, Stockholm 76(4): 291–300. 10.1111/j.1463-6395.1995.tb01001.x

[B22] Huber BA (1998) Genital mechanics in some neotropical pholcid spiders (Araneae: Pholcidae), with implications for systematics. Journal of Zoology, London 244: 587–599. 10.1111/j.1469-7998.1998.tb00063.x

[B23] Huber BA (2000) New World pholcid spiders (Araneae: Pholcidae): a revision at generic level. Bulletin of the American Museum of Natural History 254: 1–348. 10.1206/0003-0090(2000)254%3C0001:NWPSAP%3E2.0.CO;2

[B24] Huber BA (2001) The pholcids of Australia (Araneae; Pholcidae): taxonomy, biogeography, and relationships. Bulletin of the American Museum of Natural History 260: 1–144. 10.1206/0003-0090(2001)260%3C0001:TPOAAP%3E2.0.CO;2

[B25] Huber BA (2003a) Cladistic analysis of Malagasy pholcid spiders reveals generic level endemism: Revision of *Zatavua* n. gen. and *Paramicromerys* Millot (Pholcidae, Araneae). Zoological Journal of the Linnean Society 137: 261–318. 10.1046/j.1096-3642.2003.00046.x

[B26] Huber BA (2003b) High species diversity in one of the dominant groups of spiders in East African montane forests (Araneae: Pholcidae: *Buitinga* n. gen., *Spermophora* Hentz). Zoological Journal of the Linnean Society 137: 555–619. 10.1046/j.1096-3642.2003.00053.x

[B27] Huber BA (2003c) Southern African pholcid spiders: revision and cladistic analysis of *Quamtana* gen. nov. and *Spermophora* Hentz (Araneae: Pholcidae), with notes on male-female covariation. Zoological Journal of the Linnean Society 139: 477–527. 10.1046/j.0024-4082.2003.00082.x

[B28] Huber BA (2005a) Revision of the genus *Spermophora* Hentz in Southeast Asia and on the Pacific Islands, with descriptions of three new genera (Araneae: Pholcidae). Zoologische Mededelingen 79/2(4): 61–172.

[B29] Huber BA (2005b) High species diversity, male-female coevolution, and metaphyly in Southeast Asian pholcid spiders: the case of *Belisana* Thorell 1898 (Araneae, Pholcidae). Zoologica 155: 1–126.

[B30] Huber BA (2007) Two new genera of small, six-eyed pholcid spiders from West Africa, and first record of *Spermophorides* for mainland Africa (Araneae: Pholcidae). Zootaxa 1635: 23–43.

[B31] Huber BA (2011a) Revision and cladistic analysis of *Pholcus* and closely related taxa (Araneae, Pholcidae). Bonner zoologische Monographien 58: 1–509.

[B32] Huber BA (2011b) Phylogeny and classification of Pholcidae (Araneae): an update. Journal of Arachnology 39: 211–222. 10.1636/CA10-57.1

[B33] Huber BA (2012) Revision and cladistic analysis of the Afrotropical endemic genus *Smeringopus* Simon, 1890 (Araneae: Pholcidae). Zootaxa 3461: 1–138.

[B34] Huber BA (2013) Revision and cladistic analysis of the Guineo-Congolian spider genus *Smeringopina* Kraus (Araneae, Pholcidae). Zootaxa 3713: 1–160. 10.11646/zootaxa.3713.1.1 25320770

[B35] Huber BA (2015) Small scale endemism in Brazil's Atlantic Forest: 14 new species of *Mesabolivar* (Araneae, Pholcidae), each known from a single locality. Zootaxa 3942: 1–60. 10.11646/zootaxa.3942.1.1 25947536

[B36] Huber BA (2016) Spider diversity and endemism in a South American hotspot: 20 new species of *Carapoia* (Araneae: Pholcidae) from Brazil’s Atlantic Forest. Zootaxa 4177: 1–69. 10.11646/zootaxa.4177.1.1 27811735

[B37] Huber BA (2017) Revision and cladistic analysis of the Southeast Asian leaf-dwelling spider genus *Calapnita* (Araneae, Pholcidae). Zootaxa 4219: 1–63. 10.11646/zootaxa.4219.1.1 28187681

[B38] Huber BA (2018) The South American spider genera *Mesabolivar* and *Carapoia* (Araneae, Pholcidae): new species and a framework for redrawing generic limits. Zootaxa 4395: 1–178. 10.11646/zootaxa.4395.1.1 29690343

[B39] Huber BA Astrin JJ (2009) Increased sampling blurs morphological and molecular species limits: revision of the Hispaniolan endemic spider genus *Tainonia* (Araneae: Pholcidae). Invertebrate Systematics 23: 281–300. 10.1071/IS09017

[B40] Huber BA Benjamin S (2005) The pholcid spiders from Sri Lanka: redescription of *Pholcusceylonicus* and description of *Wanniyala* new genus (Araneae: Pholcidae). Journal of Natural History 39(37): 3305–3319. 10.1080/00222930500145123

[B41] Huber BA Brescovit AD (2003) *Ibotyporanga* Mello-Leitão: tropical spiders in Brazilian semi-arid habitats (Araneae: Pholcidae). Insect Systematics and Evolution 34: 15–20. 10.1163/187631203788964926

[B42] Huber BA Dimitrov D (2014) Slow genital and genetic but rapid non-genital and ecological differentiation in a pair of spider species (Araneae, Pholcidae). Zoologischer Anzeiger 253: 394–403. 10.1016/j.jcz.2014.04.001

[B43] Huber BA Eberhard WG (1997) Courtship, copulation, and genital mechanics in *Physocyclusglobosus* (Araneae, Pholcidae). Canadian Journal of Zoology 74: 905–918. 10.1139/z97-109

[B44] Huber BA Fleckenstein N (2008) Comb-hairs on the fourth tarsi in pholcid spiders (Araneae, Pholcidae). Journal of Arachnology 36: 232–240. 10.1636/CSh07-71.1

[B45] Huber BA El Hennawy H (2007) On Old World ninetine spiders (Araneae: Pholcidae), with a new genus and species and the first record for Madagascar. Zootaxa 1635: 45–53.

[B46] Huber BA Kwapong P (2013) West African pholcid spiders: an overview, with descriptions of five new species (Araneae, Pholcidae). European Journal of Taxonomy 59: 1–44. 10.5852/ejt.2013.59

[B47] Huber BA Leh Moi Ung C (2016) The *Panjangenigrifrons* group in Borneo (Araneae: Pholcidae): high diversity in Sarawak, apparent absence in Sabah. European Journal of Taxonomy 184: 1–32. 10.5852/ejt.2016.184

[B48] Huber BA Nuñeza OM (2015) Evolution of genital asymmetry, exaggerated eye stalks, and extreme palpal elongation in *Panjange* spiders (Araneae: Pholcidae). European Journal of Taxonomy 169: 1–46. 10.5852/ejt.2015.169

[B49] Huber BA Rheims CA (2011) Diversity and endemism of pholcid spiders in Brazil’s Atlantic Forest, with descriptions of four new species of the Atlantic Forest endemic genus *Tupigea* (Araneae: Pholcidae). Journal of Natural History 45: 275–301. 10.1080/00222933.2010.524319

[B50] Huber BA Pérez-G A Baptista RLC (2005a) *Leptopholcus* (Araneae: Pholcidae) in continental America: rare relicts in low precipitation areas. Bonner zoologische Beiträge 53(1/2): 99–107.

[B51] Huber BA Rheims CA Brescovit AD (2005b) Two new species of litter-dwelling *Metagonia* spiders (Araneae, Pholcidae) document both rapid and slow genital evolution. Acta Zoologica (Stockholm) 86: 33–40. 10.1111/j.0001-7272.2005.00184

[B52] Huber BA Sinclair B Schmitt M (2007) The evolution of asymmetric genitalia in spiders and insects. Biological Reviews 82: 647–698. 10.1111/j.1469-185X.2007.00029.x 17944621

[B53] Huber BA Fischer N Astrin JJ (2010) High level of endemism in Haiti's last remaining forests: a revision of *Modisimus* (Araneae: Pholcidae) on Hispaniola, using morphology and molecules. Zoological Journal of the Linnean Society 158: 244–299. 10.1111/j.1096-3642.2009.00559.x

[B54] Huber BA Pérez-González A Astrin JJ Blume C Baptista R (2013) *Litoporusiguassuensis* Mello-Leitão, 1918 (Araneae, Pholcidae): camouflaged retreat, sexual dimorphism, female color polymorphism, intra-specific genital variation, and description of the male. Zoologischer Anzeiger 252: 511–521. 10.1016/j.jcz.2012.12.001

[B55] Huber BA Carvalho LS Benjamin SP (2014) On the New World spiders previously misplaced in *Leptopholcus*: molecular and morphological analyses and descriptions of four new species (Araneae, Pholcidae). Invertebrate Systematics 28: 432–450. 10.1071/IS13050

[B56] Huber BA Nuñeza OM Leh Moi Ung C (2015) Revision, phylogeny, and microhabitat shifts in the Southeast Asian spider genus *Aetana* (Araneae, Pholcidae). European Journal of Taxonomy 162: 1–78. 10.5852/ejt.2015.162

[B57] Huber BA Petcharad B Leh Moi Ung C Koh JKH Ghazali ARM (2016a) The Southeast Asian *Pholcushalabala* species group (Araneae, Pholcidae), new data from field observations and ultrastructure. European Journal of Taxonomy 190: 1–55. 10.5852/ejt.2016.190

[B58] Huber BA Koh JKH Ghazali ARM Nuñeza O Leh Moi Ung C Petcharad B (2016b) New leaf- and litter-dwelling species of the genus *Pholcus* from Southeast Asia (Araneae, Pholcidae). European Journal of Taxonomy 200: 1–45. 10.5852/ejt.2016.200

[B59] Huber BA Nuñeza OM Leh Moi Ung C (2016c) The Philippine hair wax spiders and their relatives: Revision of the *Pholcusbicornutus* species group (Araneae, Pholcidae). European Journal of Taxonomy 225: 1–34. 10.5852/ejt.2016.225

[B60] Japyassú HF Macagnan CR (2004) Fishing for prey: the evolution of a new predatory tactic among spiders (Araneae, Pholcidae). Revista de Etologia 6(2): 79–94.

[B61] Nguyen LT Schmidt HA von Haeseler A Minh BQ (2015) IQ-TREE: A fast and effective stochastic algorithm for estimating maximum-likelihood phylogenies. Molecular Biology and Evolution 32: 268–274. 10.1093/molbev/msu300 25371430 PMC4271533

[B62] Penney D (2007) The oldest pholcid and selenopid spiders (Araneae) in lowermost Eocene amber from the Paris Basin, France. Journal of Arachnology 34: 592–598. 10.1636/H05-61.1

[B63] Sanderson MJ Shaffer HB (2002) Troubleshooting molecular phylogenetic analyses. Annual Review of Ecology and Systematics 33: 49–72. 10.1146/annurev.ecolsys.33.010802.150509

[B64] Senglet A (1971) Note sur les Pholcidae (Arachn.) de Grèce. Bulletin de la Société entomologique de Suisse 44(3/4): 345–359.

[B65] Senglet A (2001) Copulatory mechanisms in *Hoplopholcus*, *Stygopholcus* (revalidated), *Pholcus*, *Spermophora* and *Spermophorides* (Araneae, Pholcidae), with additional faunistic and taxonomic data. Mitteilungen der schweizerischen entomologischen Gesellschaft 74: 43–67.

[B66] Simon E (1890) Etudes arachnologiques. 22e Mémoire. XXXIV. Etude sur les arachnides de l'Yemen. Annales de la Société Entomologique de France (6) 10: 77–124.

[B67] Simon E (1893) Histoire Naturelle des Araignées, 2^eme^ ed. 1 (2): 256–488. Paris.

[B68] Sørensen LL Coddington JA Scharff N (2002) Inventorying and estimating subcanopy spider diversity using semiquantitative sampling methods in an Afromontane forest. Environmental Entomology 31(2): 319–330. 10.1603/0046-225X-31.2.319

[B69] Stamatakis A (2014) RAxML version 8: a tool for phylogenetic analysis and post-analysis of large phylogenies. Bioinformatics 30: 1312–1333. 10.1093/bioinformatics/btu033 24451623 PMC3998144

[B70] Stamatakis A Hoover P Rougemont J (2008) A rapid bootstrap algorithm for the RAxML web servers. Systematic Biology 57: 758–771. 10.1080/10635150802429642 18853362

[B71] Wilkinson M (1996) Majority-rule reduced consensus trees and their use in bootstrapping. Molecular Biology and Evolution 13: 437–444. 10.1093/oxfordjournals.molbev.a025604 8742632

[B72] World Spider Catalog (2018) World Spider Catalog. Natural History Museum Bern. http://wsc.nmbe.ch [version 19.0, accessed on 9 May 2018]

