# Supplementary figures and images for: The phylogeny of pholcid spiders: a critical evaluation of relationships suggested by molecular data (Araneae, Pholcidae)
Source: Zookeys. 2018 Oct 10;(789):51–101. doi: 10.3897/zookeys.789.22781 (PMC6193417; doi:10.3897/zookeys.789.22781)

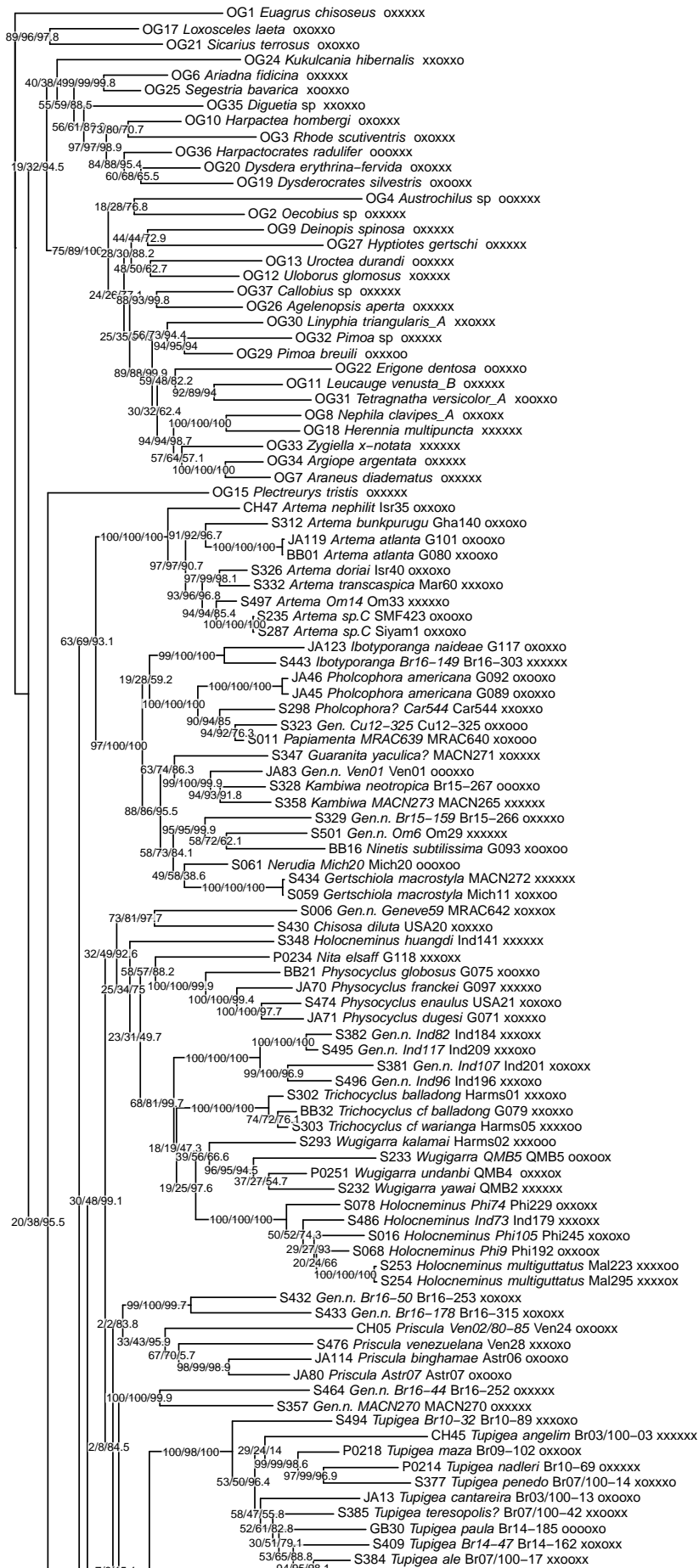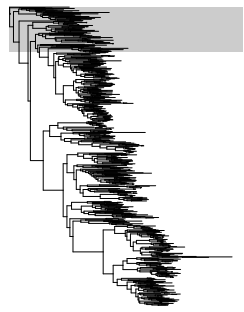

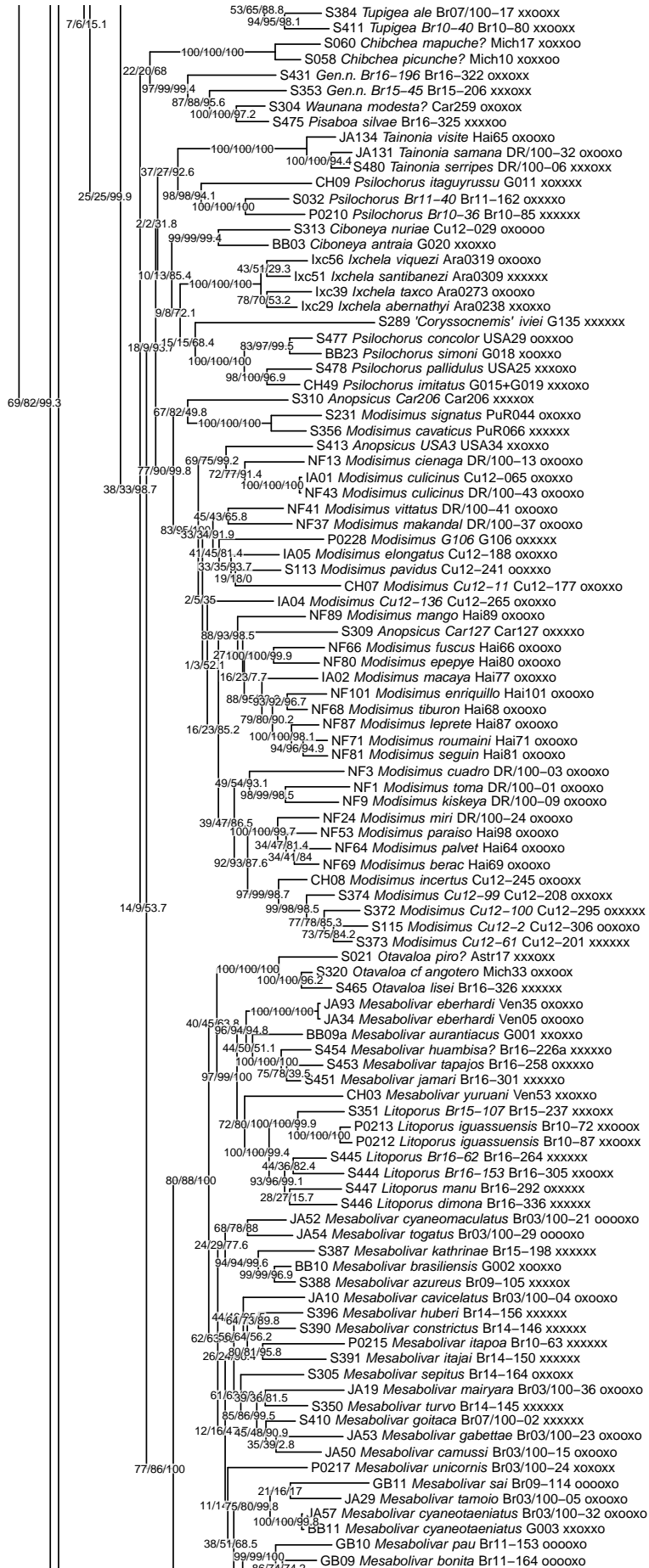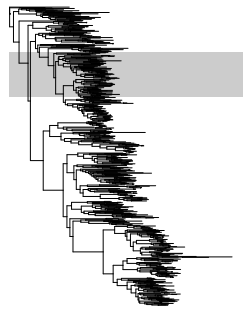

22/23/84.4

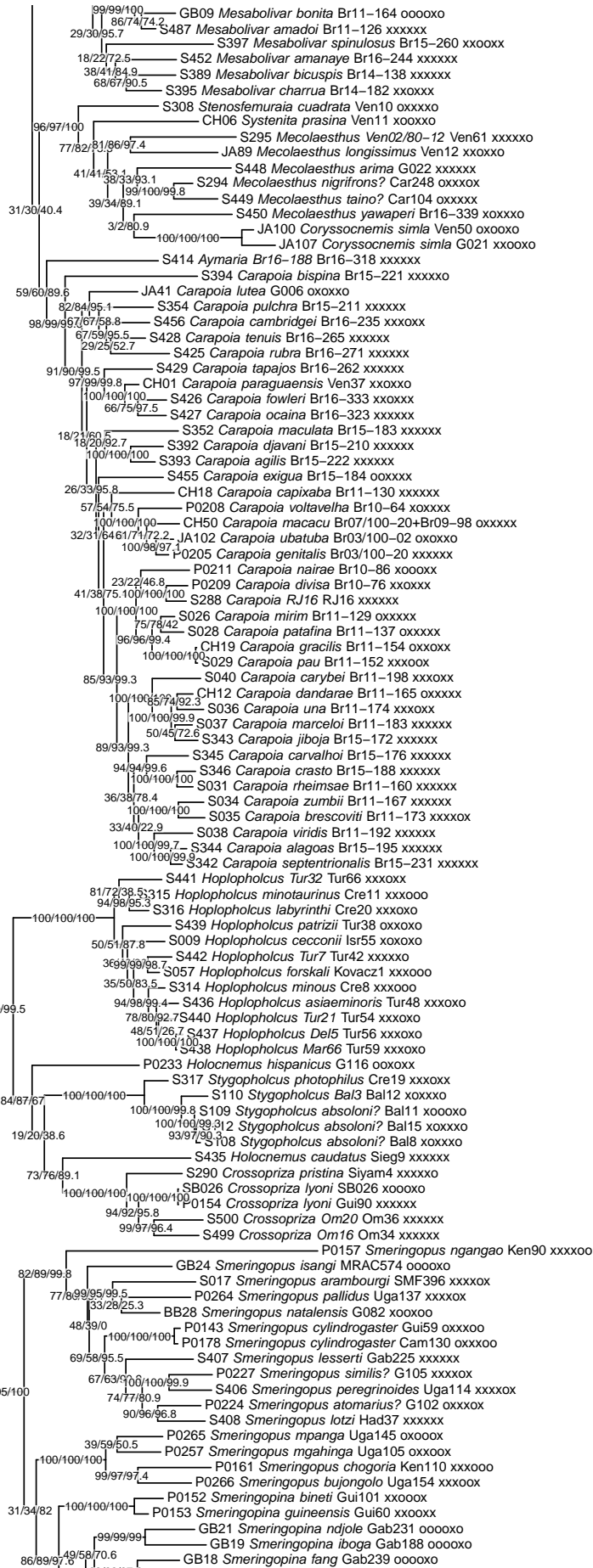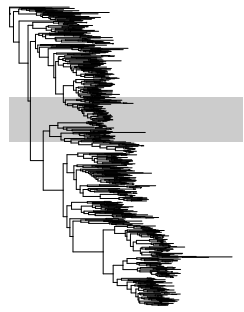

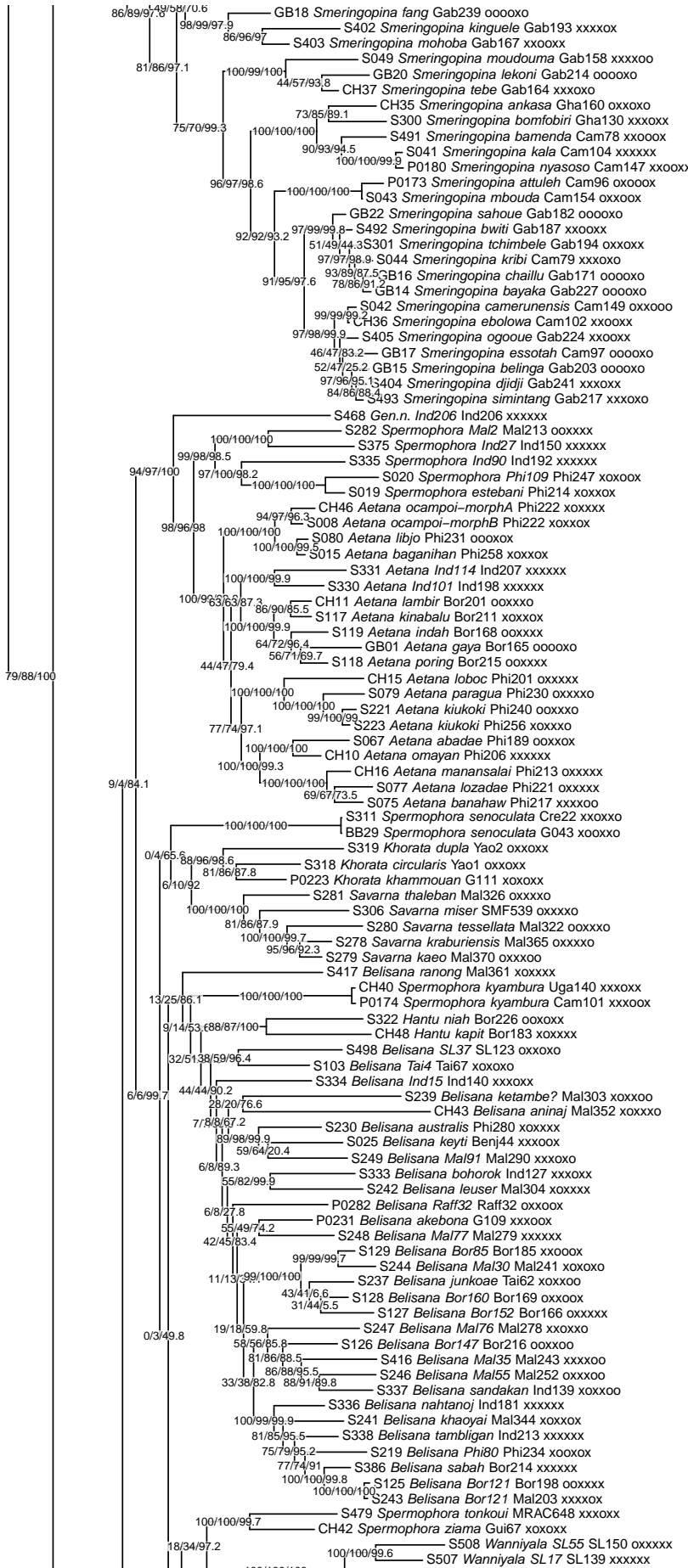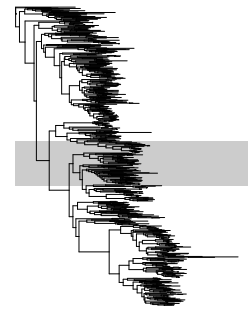

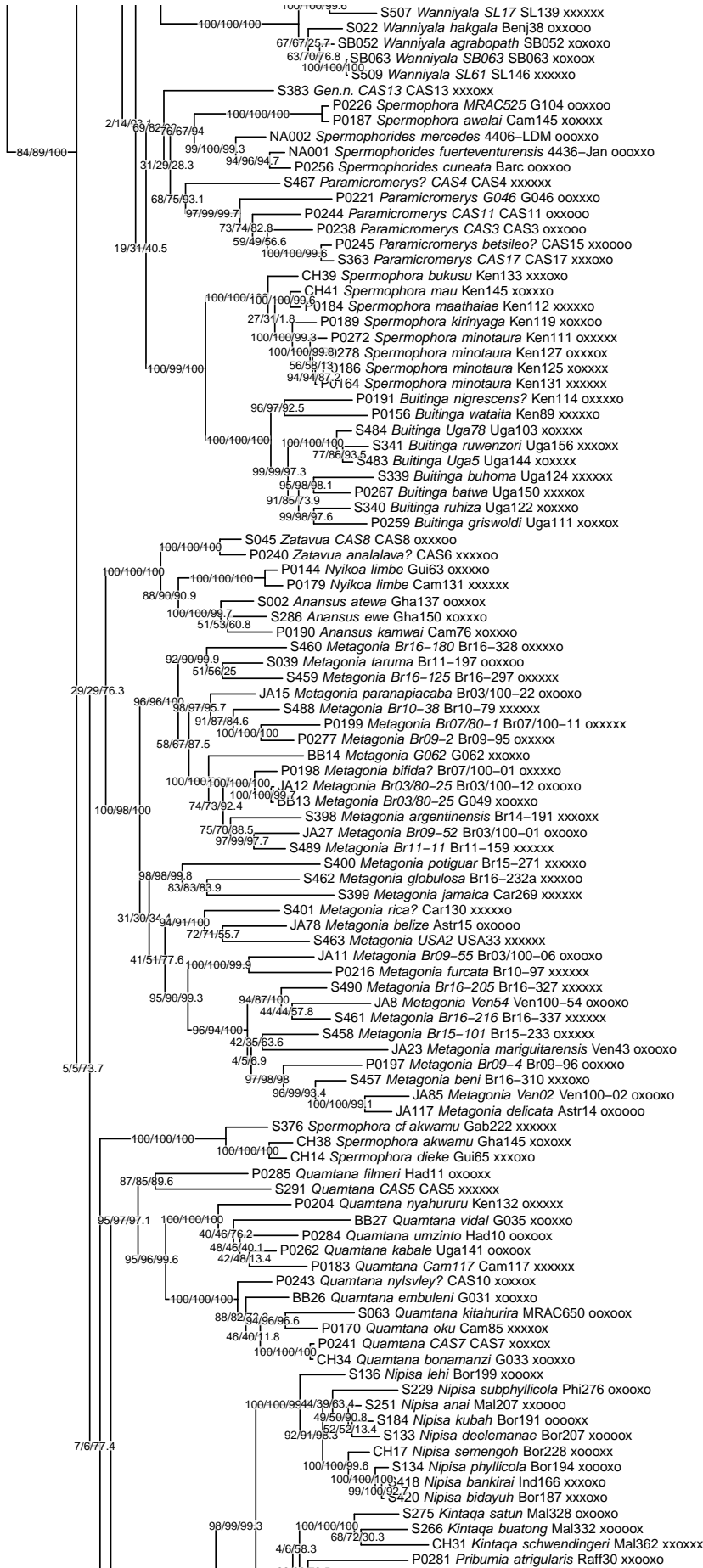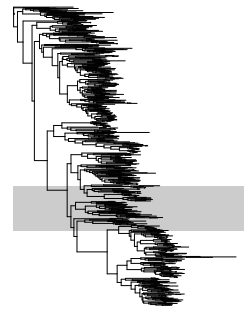

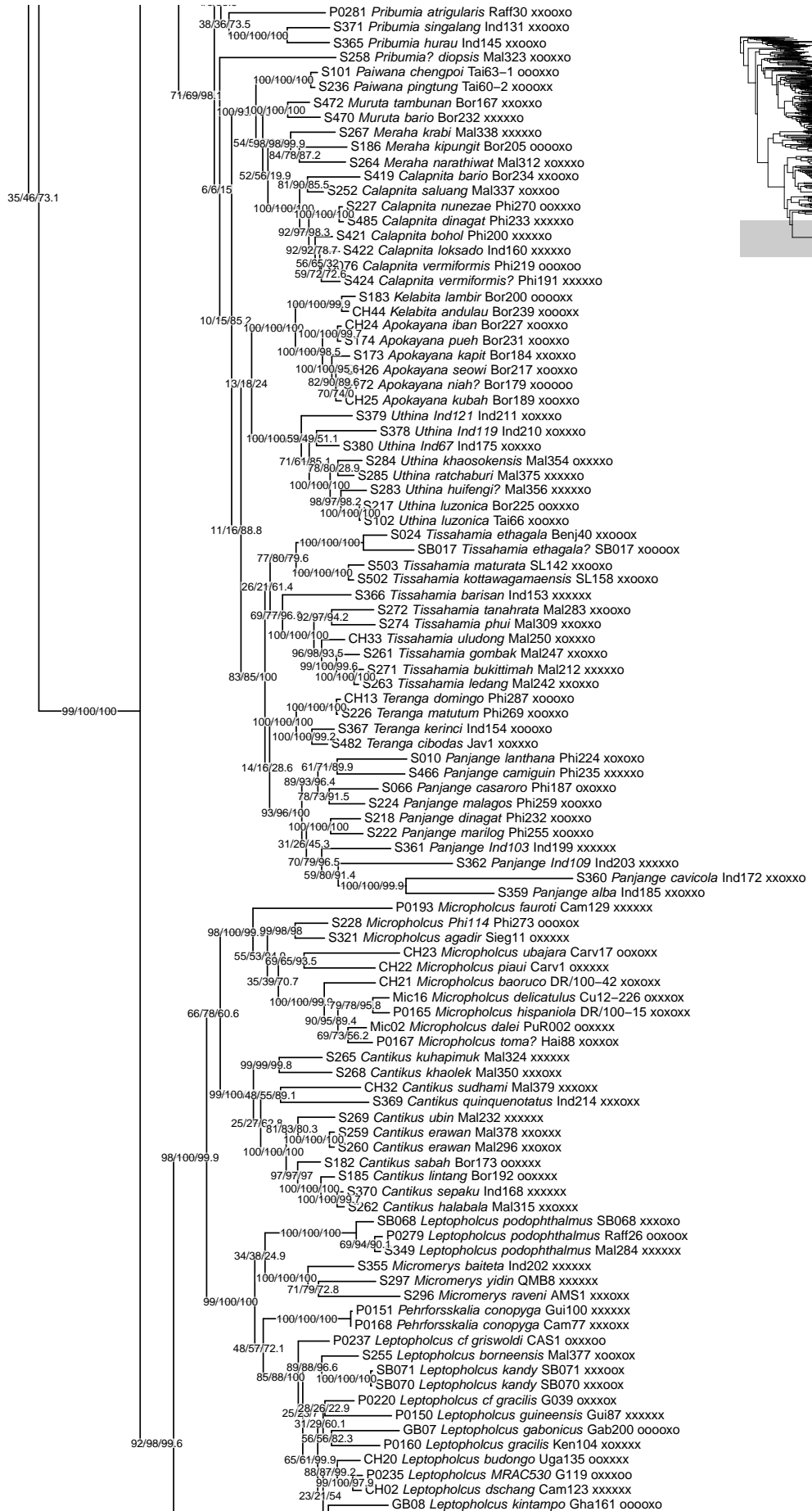

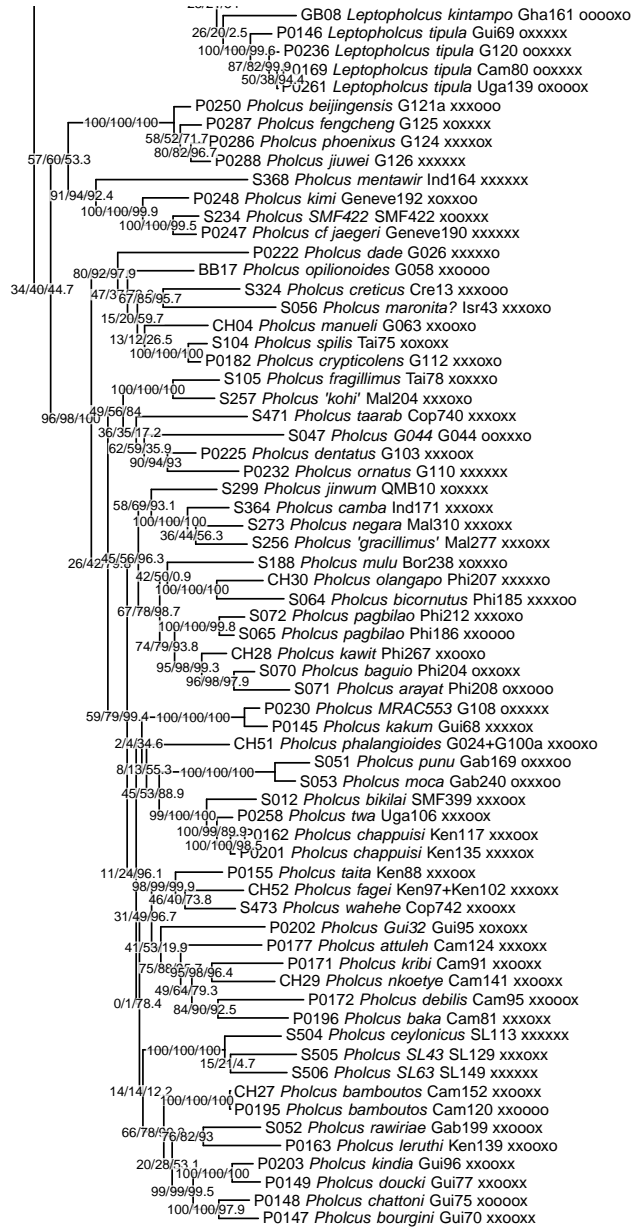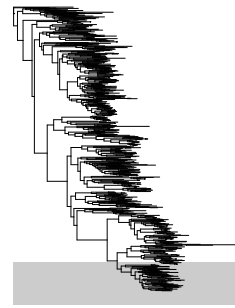

Supplement: Supplementary material 1 — Figure S1. Maximum-likelihood tree of the complete set of taxa inferred with IQ-TREE [file zookeys-789-051-s001.pdf]

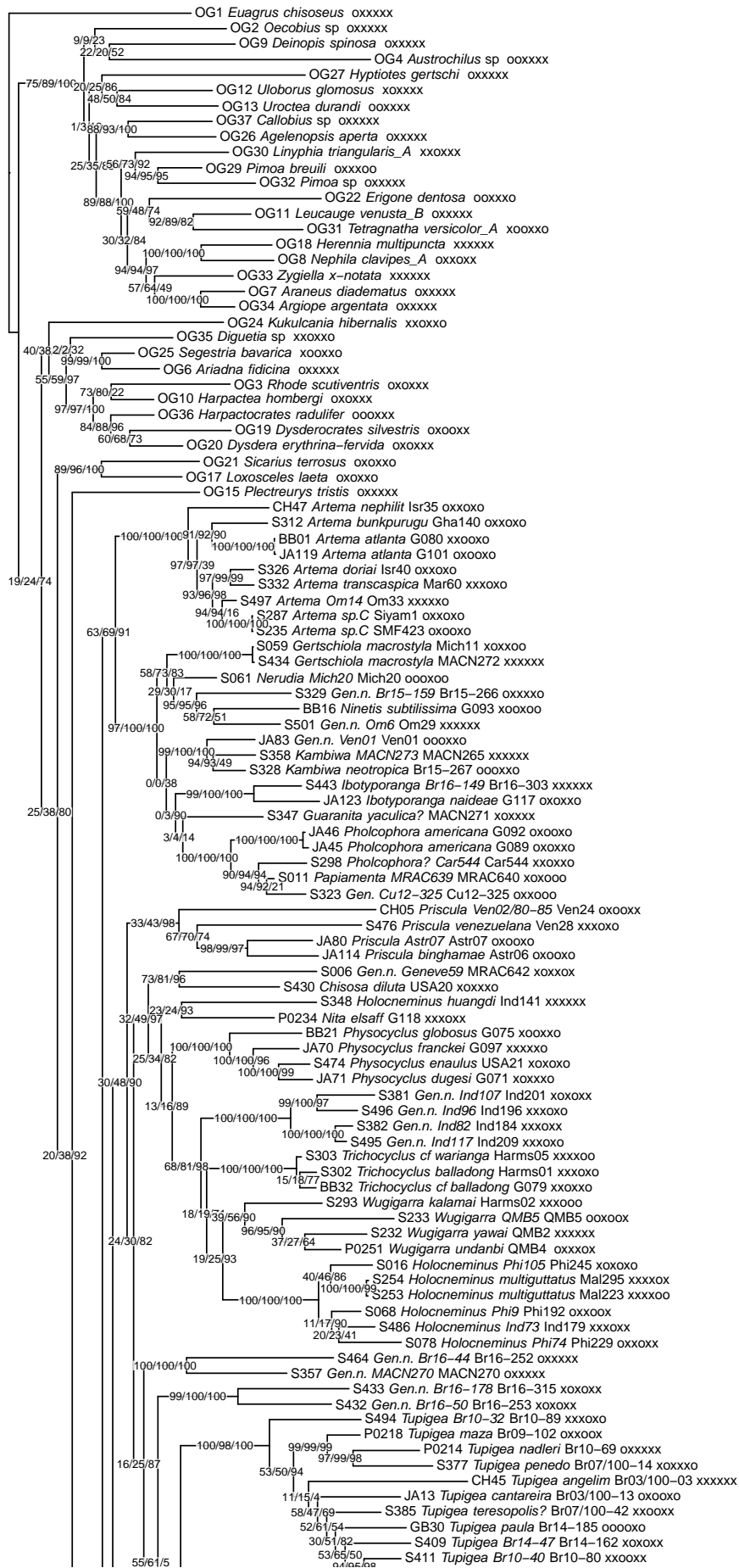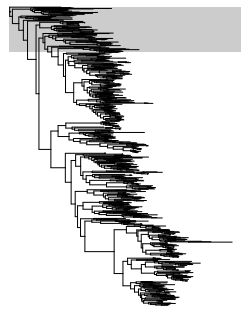

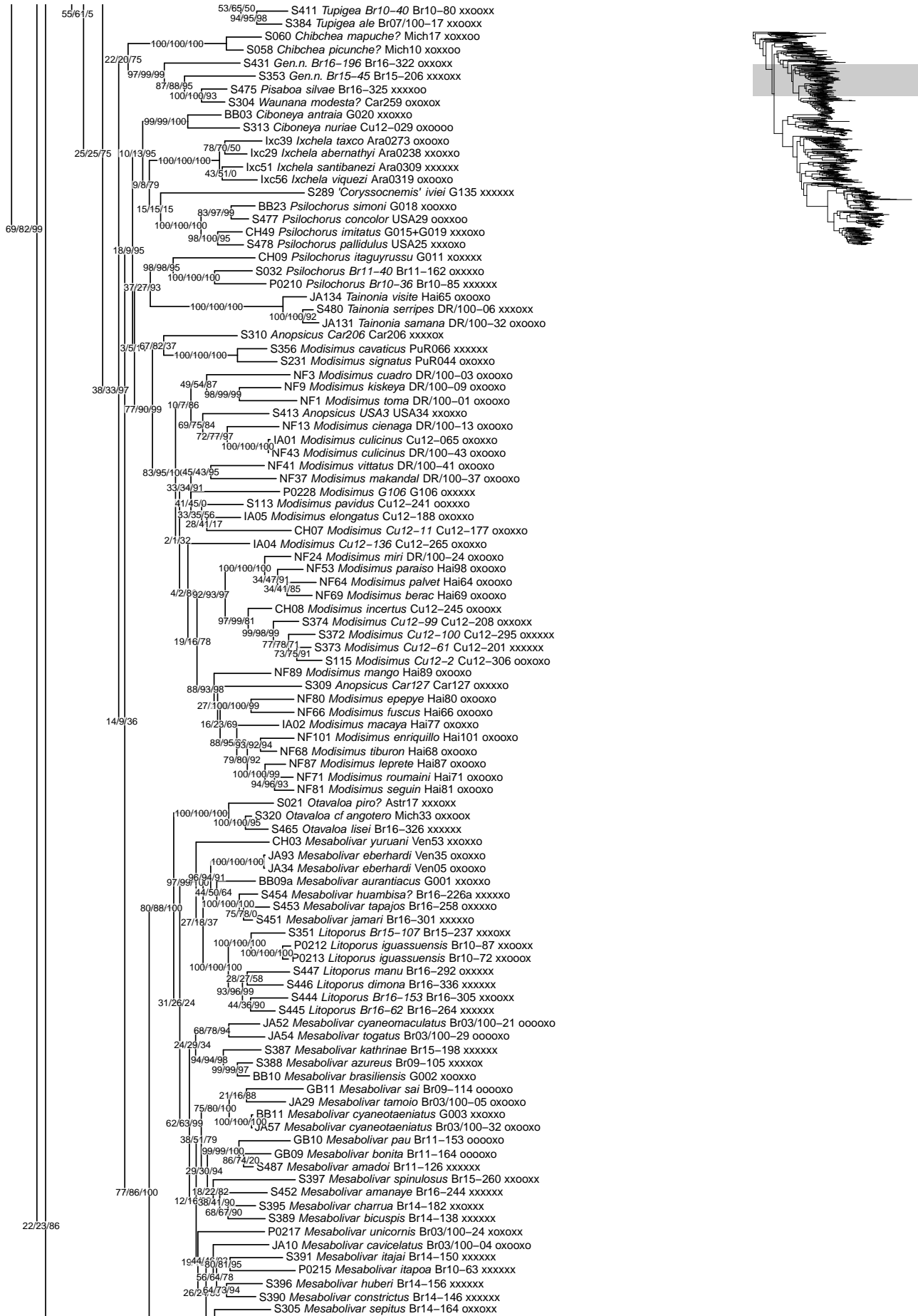

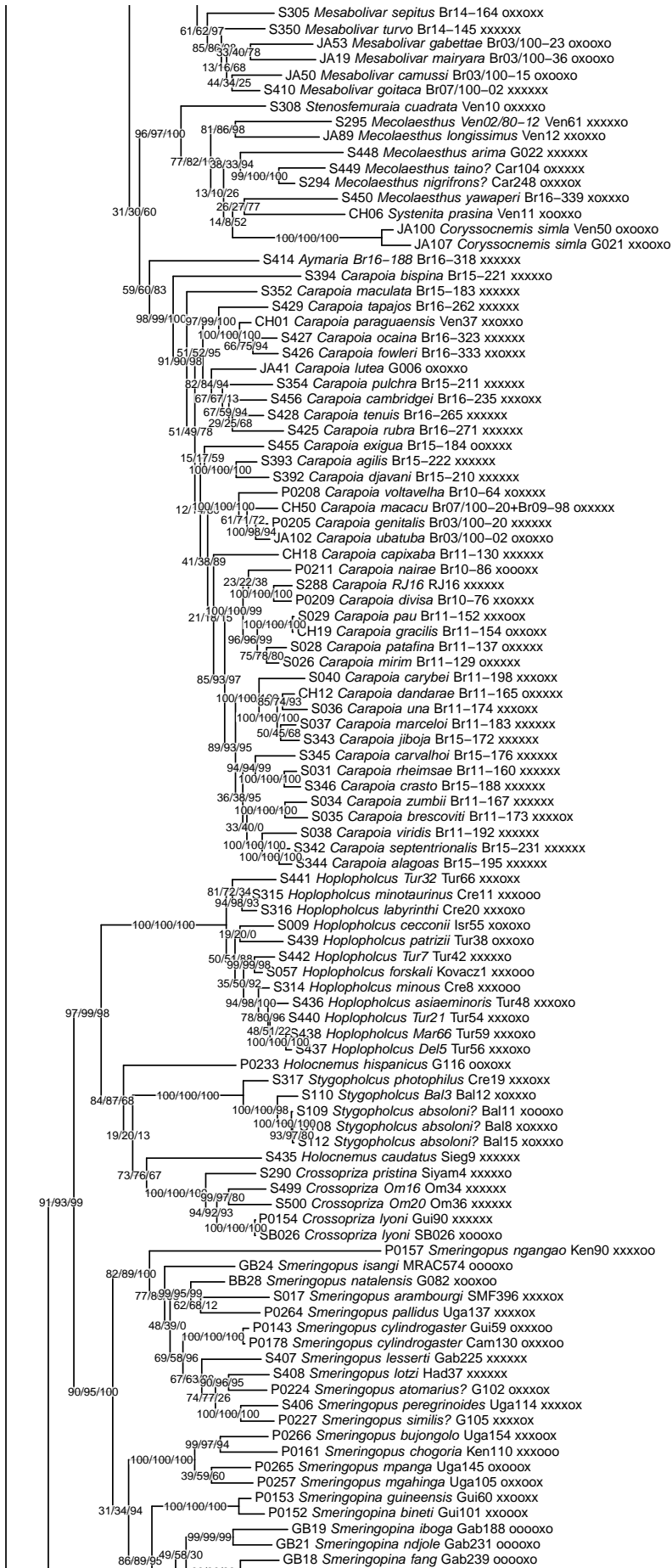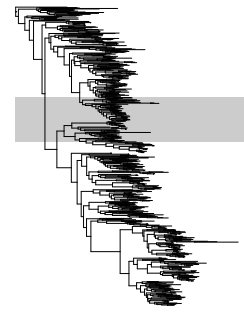

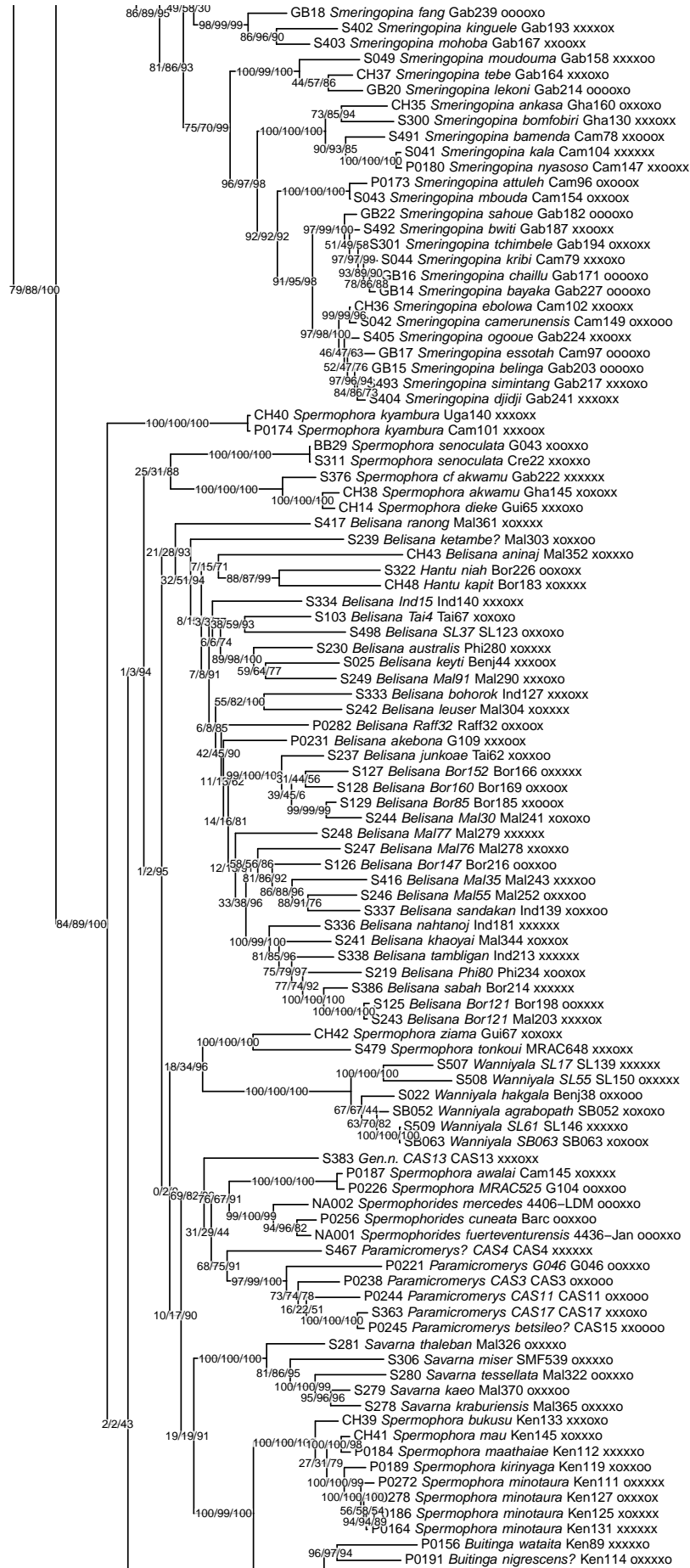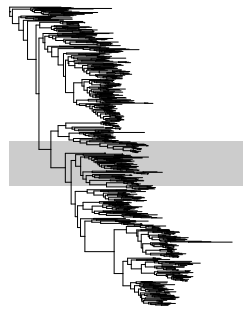

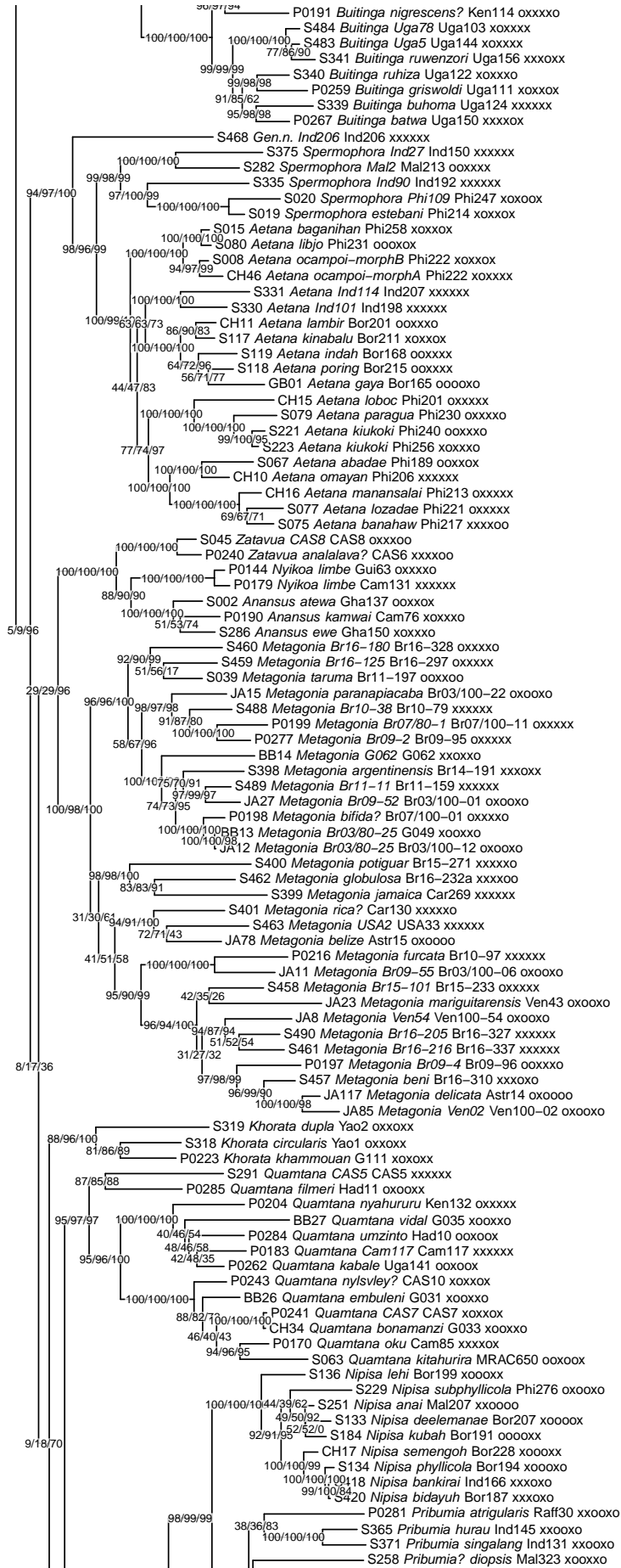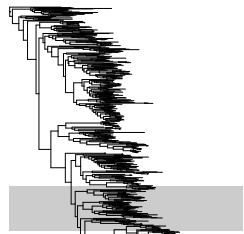

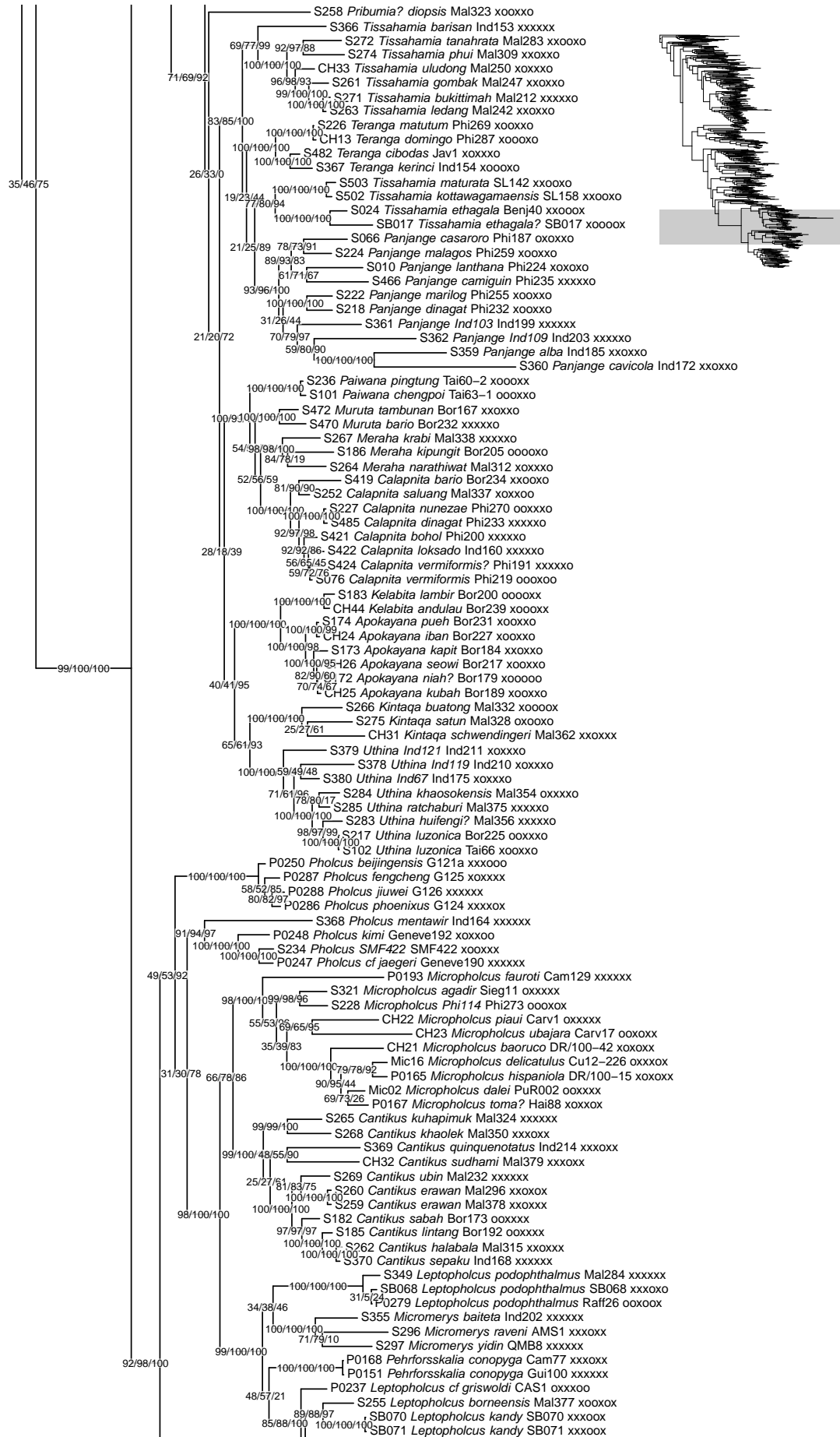

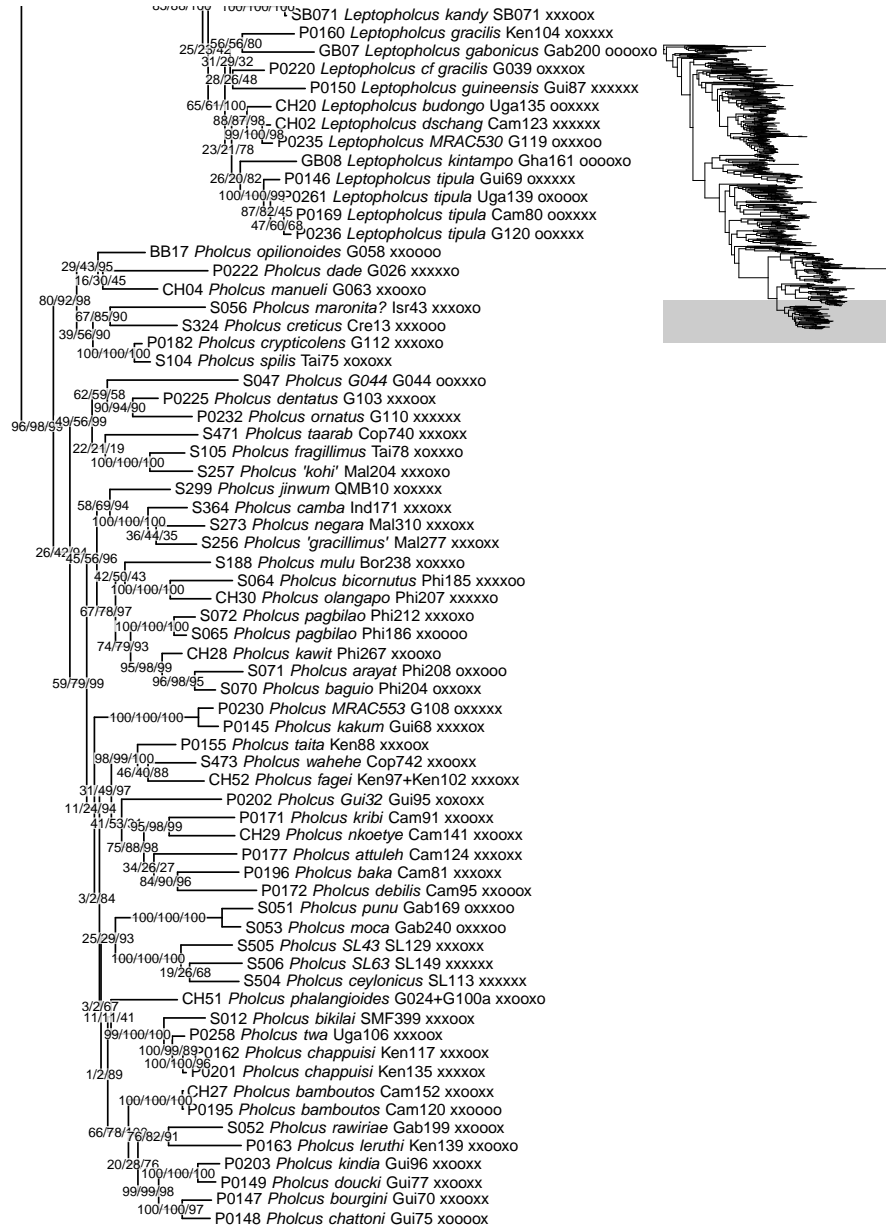

Supplement: Supplementary material 2 — Figure S2. Maximum-likelihood tree of the complete set of taxa inferred with RAxML [file zookeys-789-051-s002.pdf]

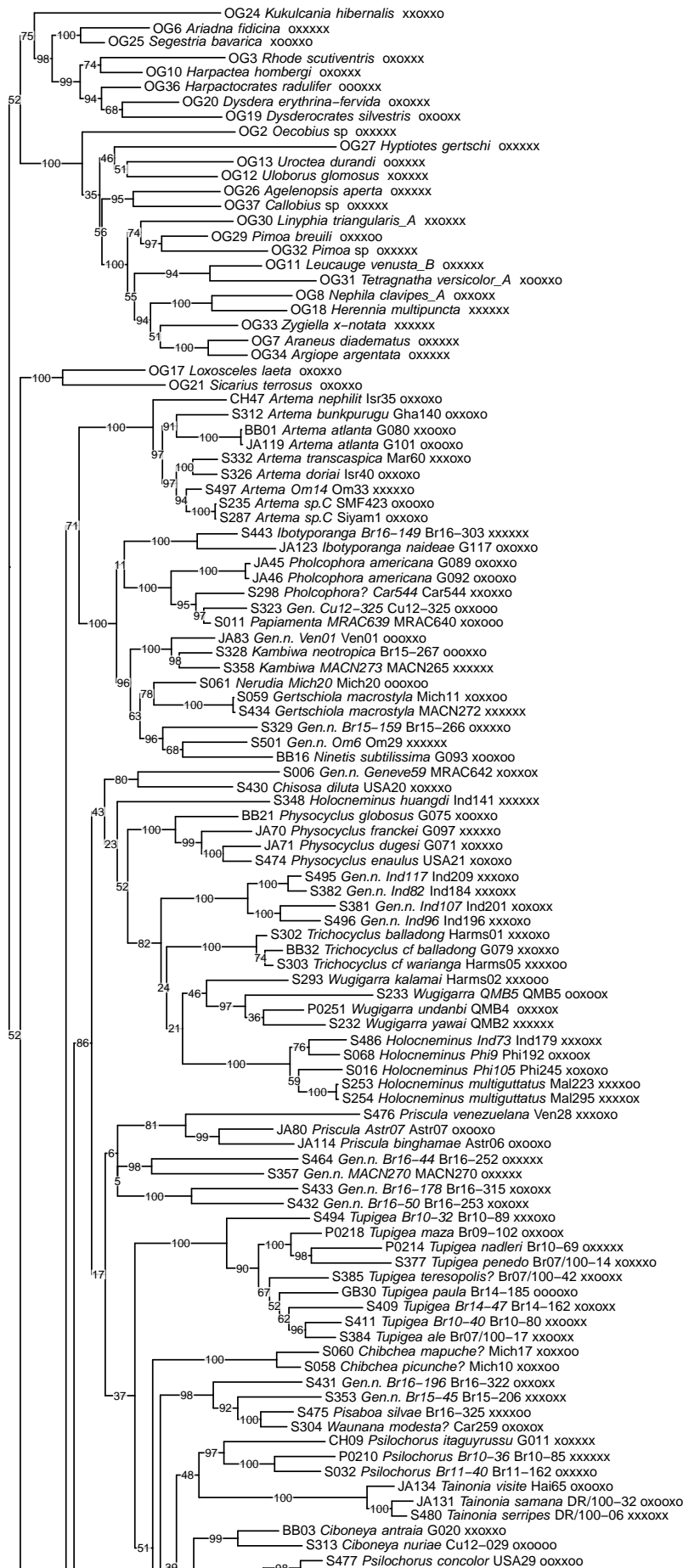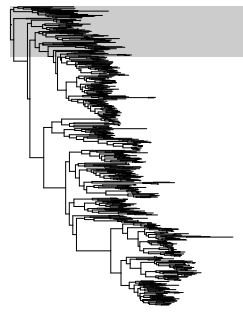

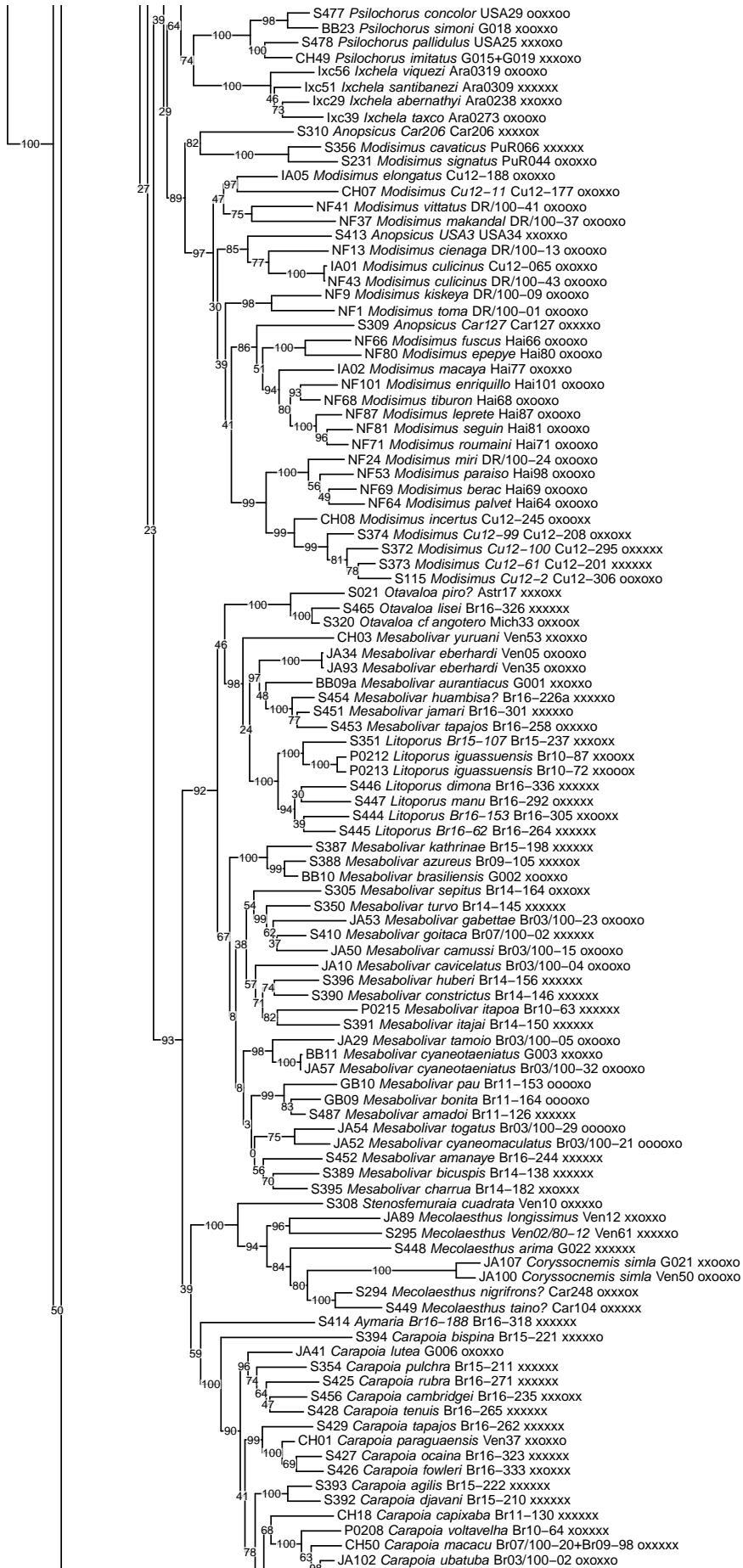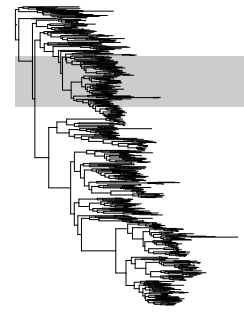

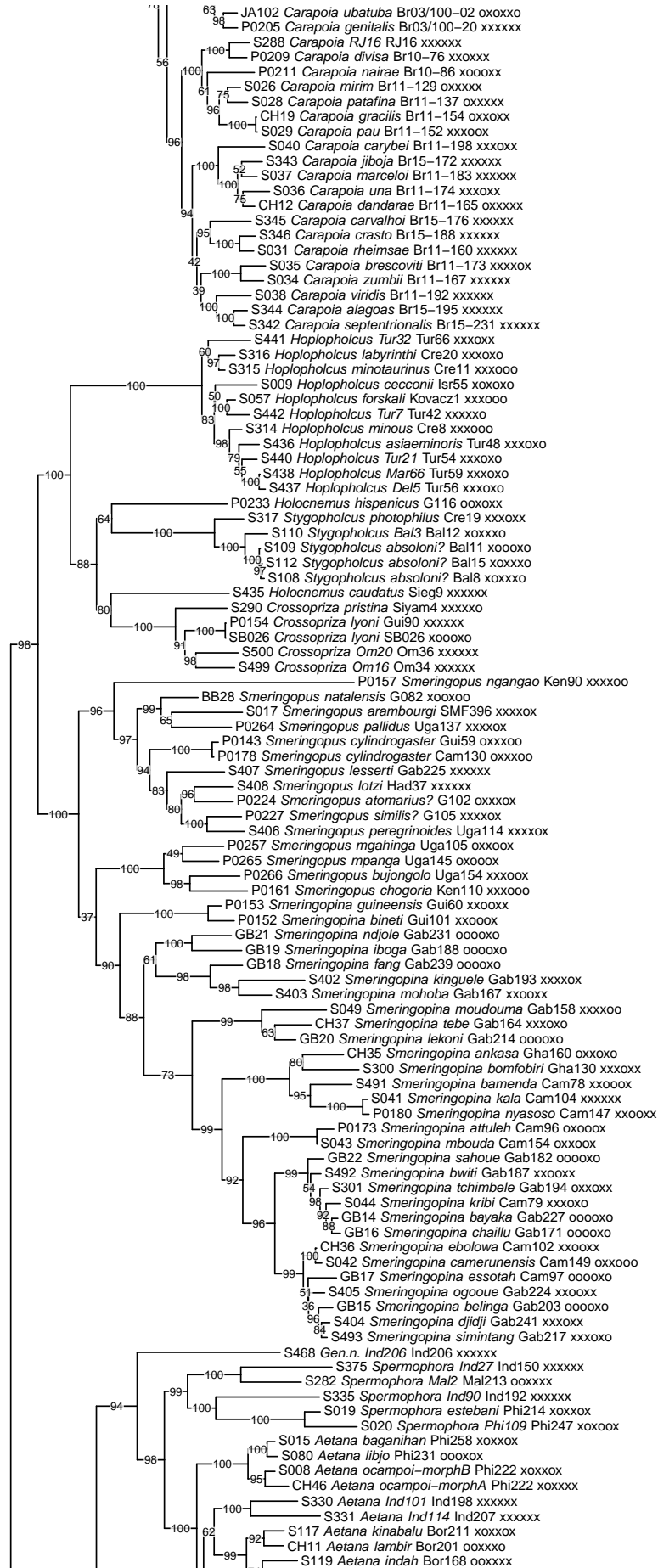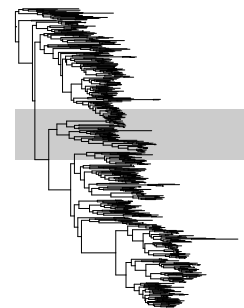

99

9

8

3

14

36

32

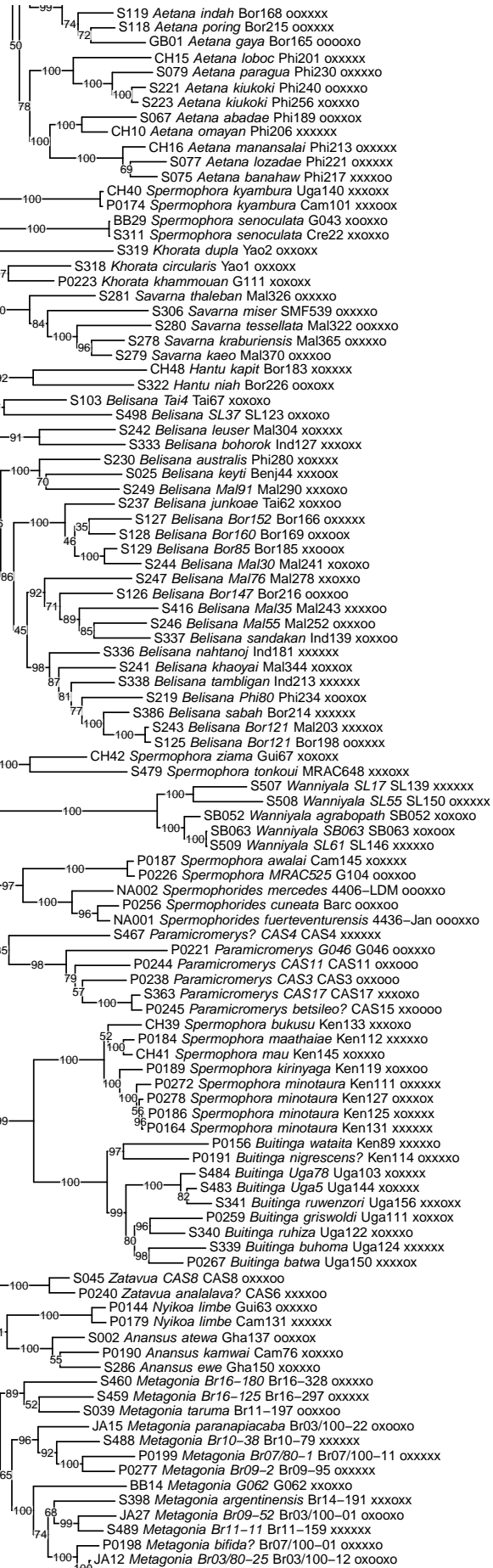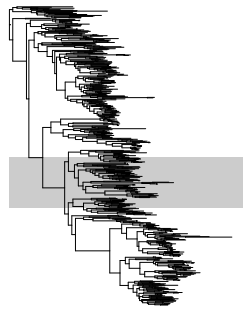

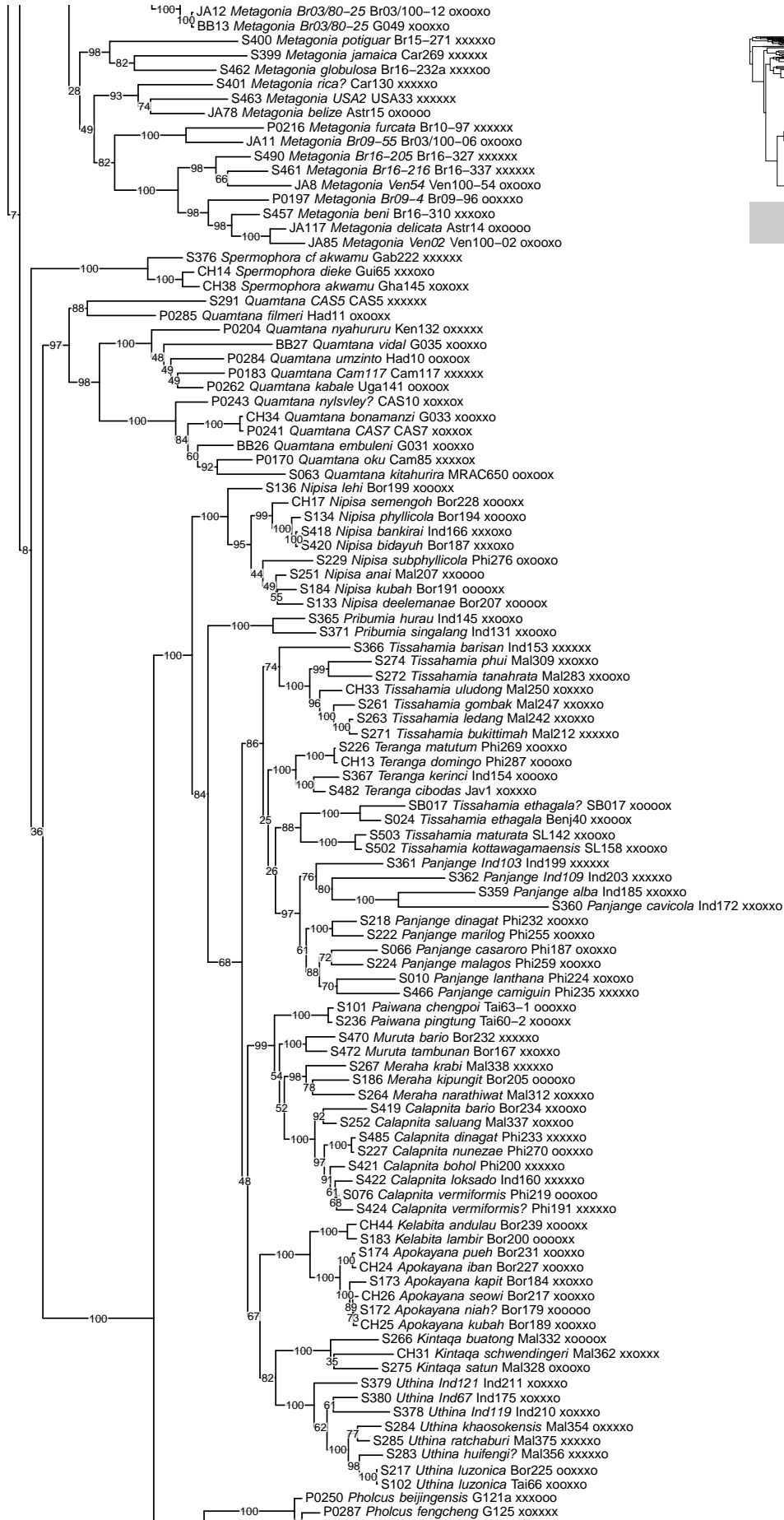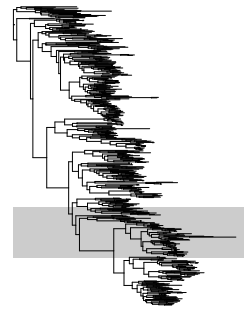

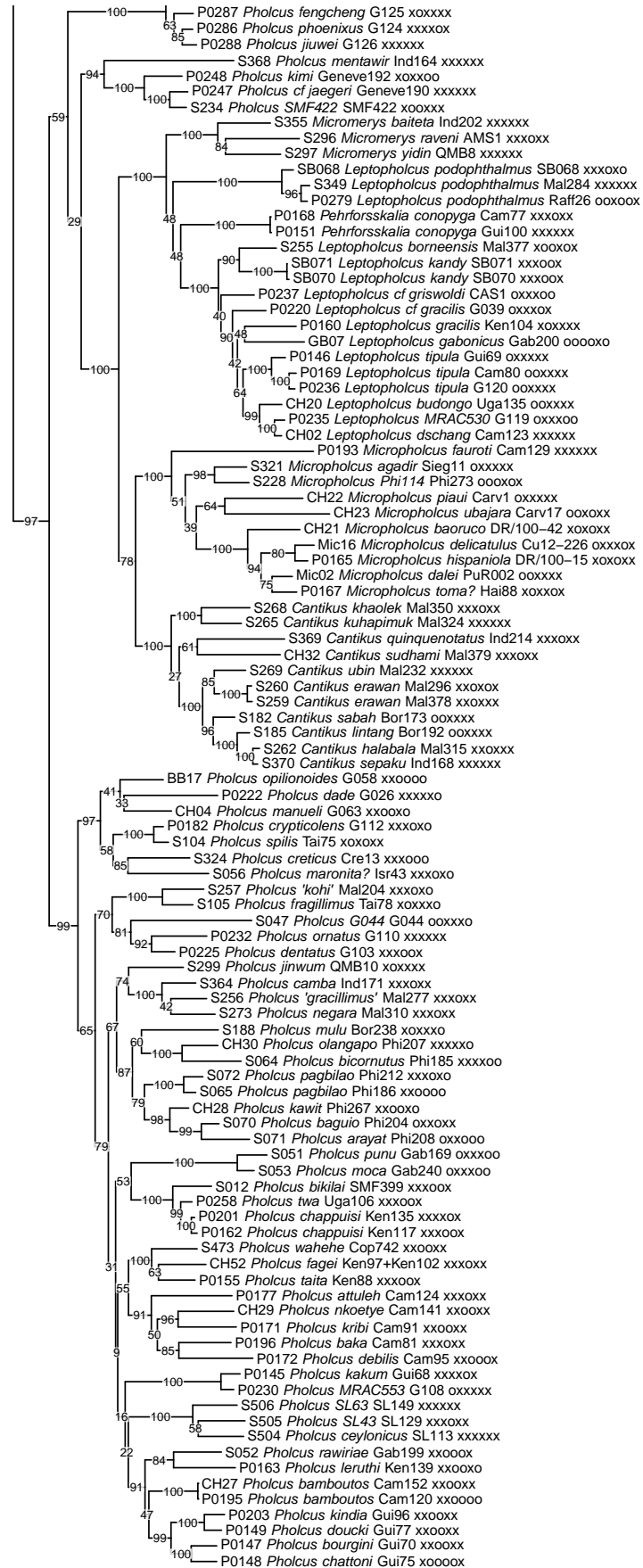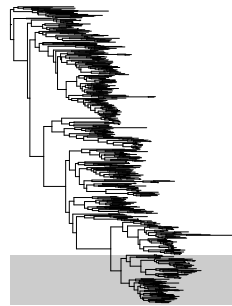

Supplement: Supplementary material 3 — Figure S3. Maximum-likelihood tree (RAxML) of a reduced set of taxa (excluding rogue taxa with RogueNaRok) [file zookeys-789-051-s003.pdf]

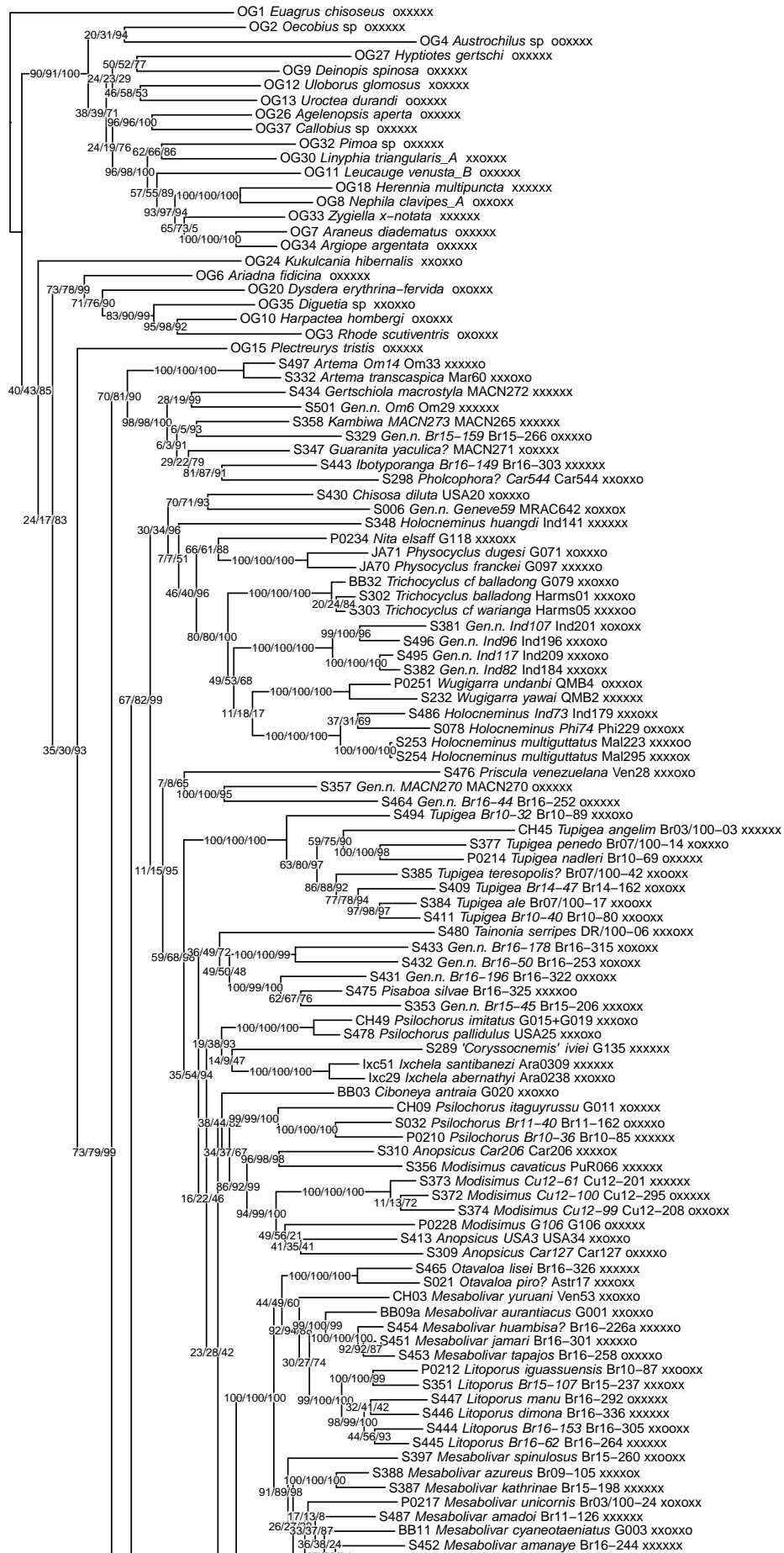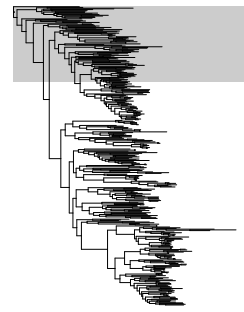

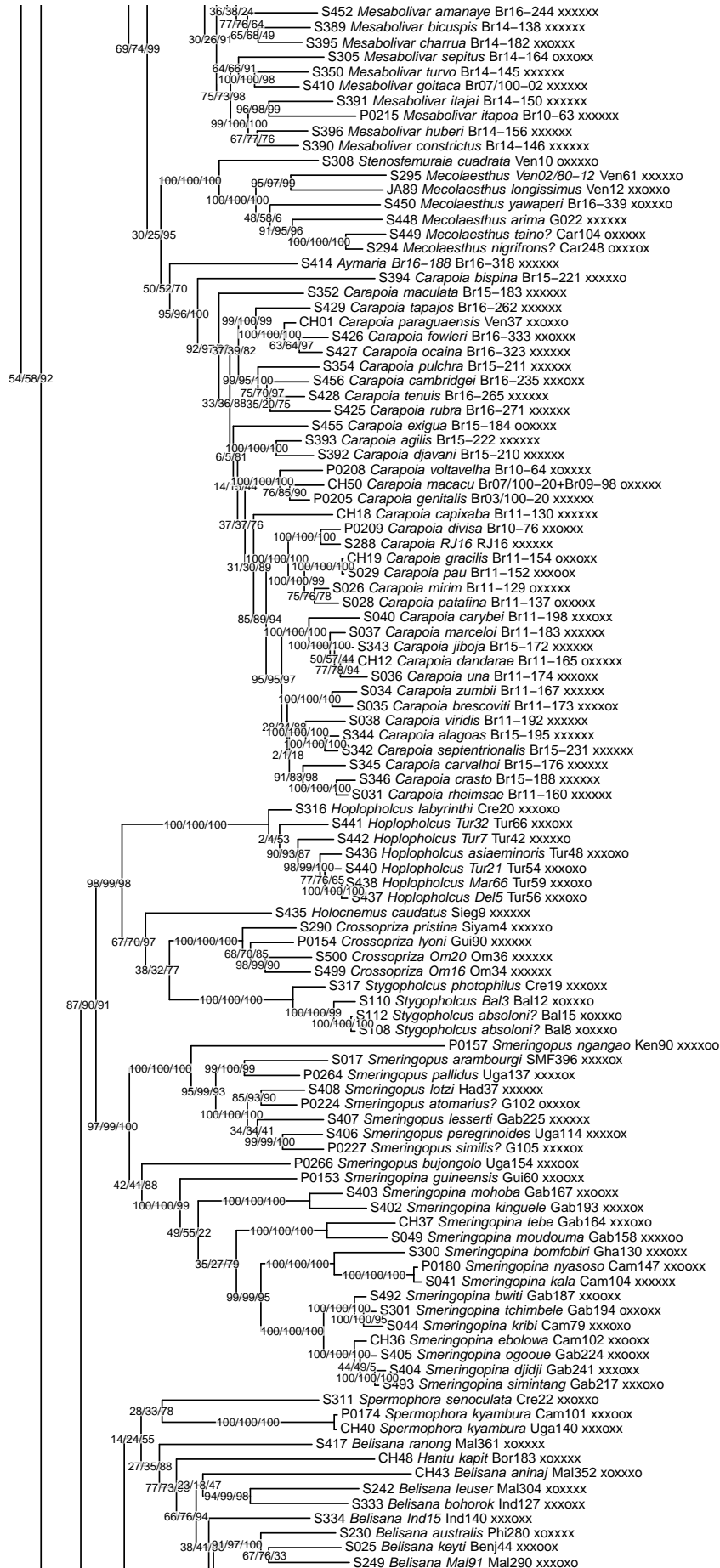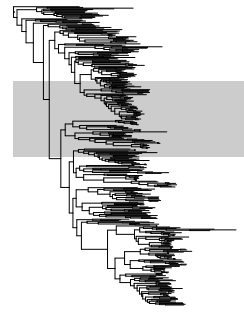

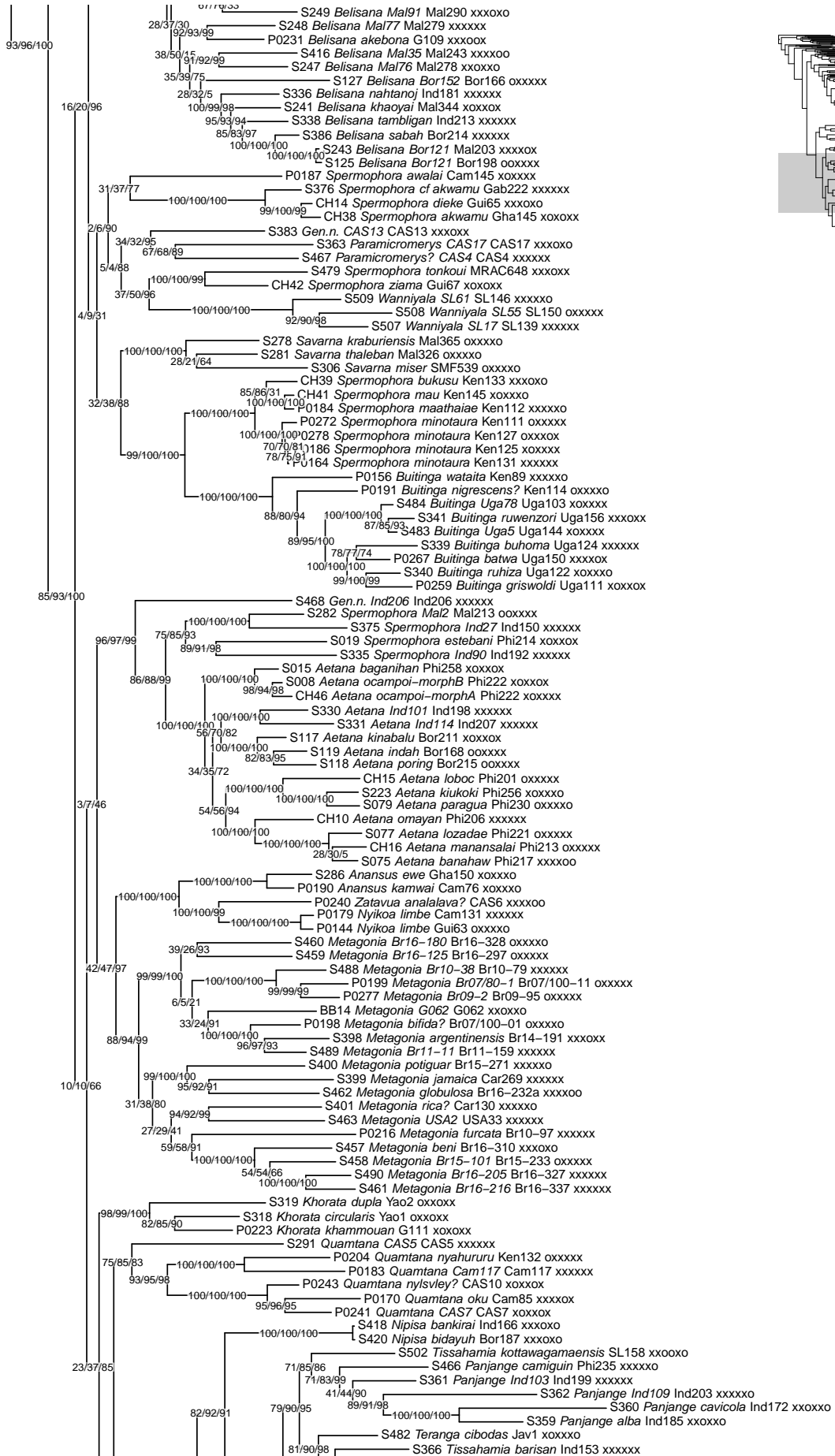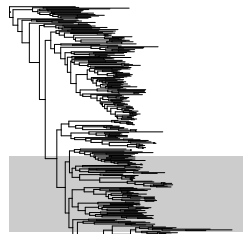

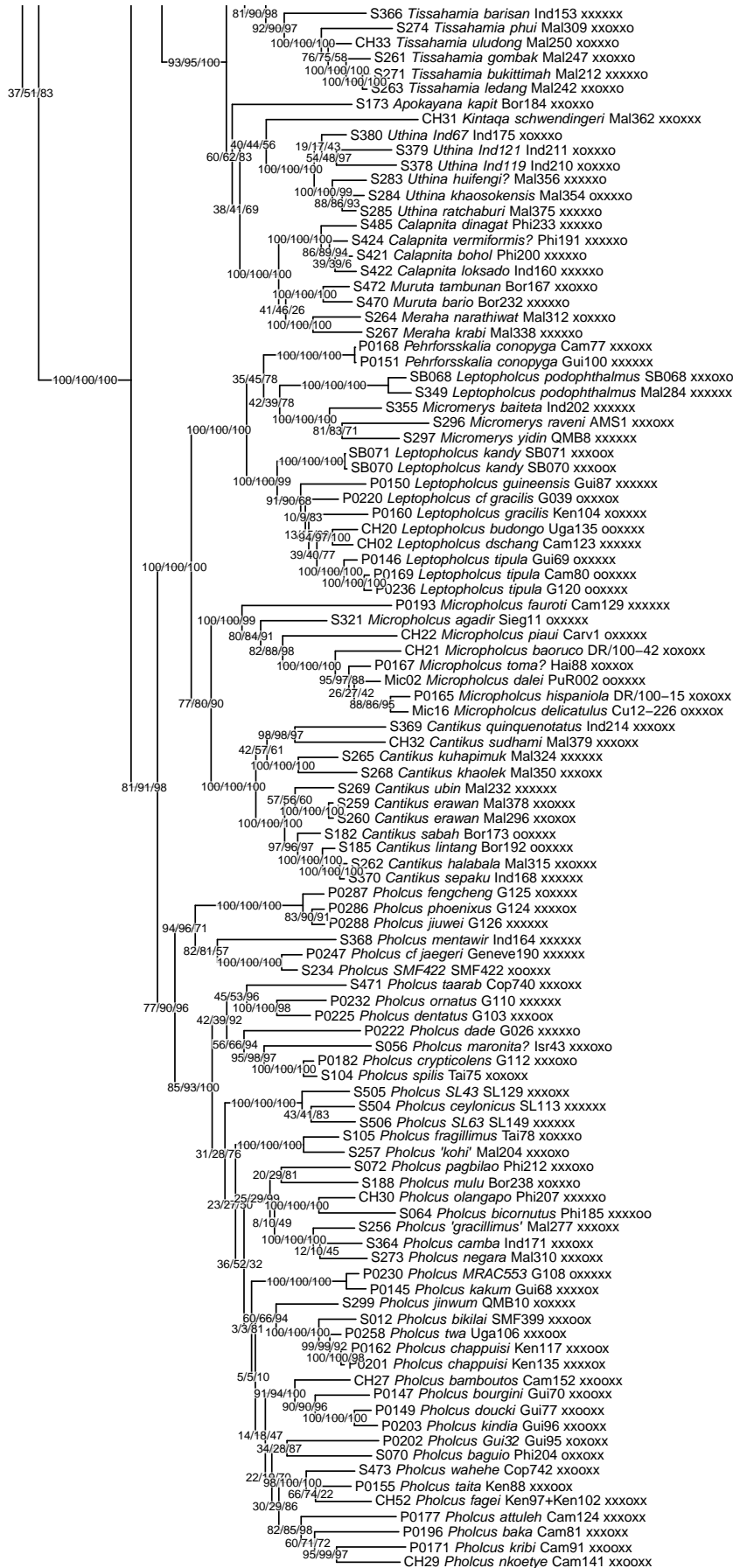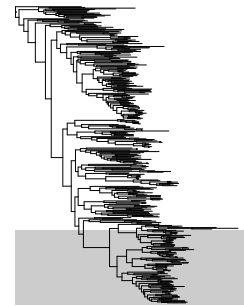

Supplement: Supplementary material 4 — Figure S4. Maximum-likelihood tree (RAxML) of a reduced set of taxa (excluding taxa for which less than four genes were available) [file zookeys-789-051-s004.pdf]
